# Supplementary material for: A Rapid Review on the Influence of COVID-19 Lockdown and Quarantine Measures on Modifiable Cardiovascular Risk Factors in the General Population
Source: Int J Environ Res Public Health. 2021 Aug 13;18(16):8567. doi: 10.3390/ijerph18168567 (PMC8393482; doi:10.3390/ijerph18168567)
Supplement: Supplementary file 1 [file ijerph-18-08567-s001.zip › ijerph-1300479-supplementary.pdf]

TABLE OF CONTENTS

Table of contents 1
S1 Methods 2
S1.1 Search strings 2
S2 Results 4
S2.1 Excluded studies 4
S2.1.1 Studies about patient cohorts (not reported within this paper) 4
S2.1.2 Excluded Fulltexts – Reason: Topically relevant study, but using non-probabilistic sampling 6
S2.1.3 Excluded Fulltexts – Reason: Study design 18
S2.1.4 Excluded Fulltexts – Reason: Missing reference values 22
S2.1.5 Excluded Fulltexts – Reason: Unclear recruitment procedure 25
S2.1.6 Excluded Fulltexts – Reason: Outcome 27
S2.1.7 Excluded Fulltexts – Reason: Language 29
S2.1.8 Excluded Fulltexts – Reason: Population 30
S2.1.9 Excluded Fulltexts – Reason: Double publication 31
S2.2 Results of data extraction 32

S1 METHODS

S1.1 SEARCH STRINGS

Table S1: Search string Pubmed

#	Search terms
**1**	(coronavirus OR “corona virus” OR coronavirinae OR coronaviridae OR betacoronavirus OR covid19 OR “covid 19” OR nCoV OR “CoV 2” OR CoV2 OR sarscov2 OR 2019nCoV OR “novel CoV” OR “wuhan virus”) OR ((wuhan OR hubei OR huanan) AND (“severe acute respiratory” OR pneumonia) AND (outbreak)) OR “Coronavirus”[Mesh] OR “Coronavirus Infections”[Mesh] OR “COVID-19” [Supplementary Concept] OR “severe acute respiratory syndrome coronavirus 2” [Supplementary Concept] OR “Betacoronavirus”[Mesh]
**2**	“social isolation”[tw] OR “physical isolation”[tw] OR self-isolation[tw] OR ”patient isolation”[tw] OR "social isolation"[mh] OR quarantine[tw] OR self-quarantine[tw] OR quarantine[mh] OR “social distancing”[tw] OR “physical distancing”[tw] OR lockdown[tw] OR “lock down”[tw] OR shutdown[tw] OR “shut down”[tw] OR “home confinement”[tw] OR “house arrest”[tw] OR loneliness[tw] OR loneliness[mh]
**3**	sedentary[tw] OR "sedentary behavior"[mh] OR “physical inactivity”[tw] OR "physical education and training"[mh] OR “physical training”[tw] OR “physical activity”[tw] OR “physical lifestyle”[tw] OR “physical education”[tw] OR exercis*[tw] OR exercise[mh] OR sport*[tw] OR sports[mh] OR fitness[tw] OR “physical fitness”[mh] OR exert*[tw] OR endur*[tw] OR aerobic*[tw] OR aliment*[tw]<br>OR<br>diet*[tw] OR diet[mh] OR eat[tw] OR eating[tw] OR eating[mh] OR fare[tw] OR feed*[tw] OR food*[tw] OR food[mh] OR nourishment*[tw] OR nutrit*[tw] OR "nutritional status"[mh]<br>OR<br>adipos*[tw] OR adiposity[mh] OR bodyweight[tw] OR “body weight”[tw] OR "body weight"[mh] OR obes*[tw] OR obesity[mh] OR overweight[tw] OR overweight[mh] OR weight*[tw]<br>OR<br>tobacco[tw] OR tobacco[mh] OR smok*[tw] OR smoking[mh] OR cigarette[tw] OR cigarettes[tw] OR “tobacco products”[mh] OR nicotine[tw] OR nicotine[tw]<br>OR<br>wine*[tw] OR liquor*[tw] OR spirits[tw] OR beer*[tw] OR alcohol*[tw] OR drink*[tw] OR "Alcohol-Related Disorders"[mh] OR “alcohol drinking”[mh] or “drinking behavior”[mh] OR “alcoholic beverages”[mh]<br>OR

	"cardiovascular diseases"[mh] OR cardiovascular[tw] OR cardia*[tw] OR heart*[tw] OR coronary*[tw] OR angina*[tw] OR ventric*[tw] OR myocard*[tw] OR pericard*[tw] OR ischem*[tw] OR ischaem*[tw] OR emboli*[tw] OR arrhythmi*[tw] OR thrombos*[tw] OR thromboc*[tw] OR thrombop*[tw] OR atrial fibrillat*[tw] OR tachycardia*[tw] OR endocardi*[tw] OR “sick sinus”[tw] OR "cerebrovascular disorders"[mh] OR stroke[tw] OR strokes[tw] OR cerebrovasc*[tw] OR “cerebral vascular”[tw] OR apoplexy[tw] OR “brain accident”[tw] OR ((brain*[tw] OR cerebral[tw] OR lacunar[tw]) AND infarct*[tw]) OR "hypertension"[mh] OR hypertensi*[tw] OR "peripheral arterial disease"[tw] OR ((high[tw] OR increased[tw] OR elevated[tw]) AND “blood pressure”[tw]) OR "hyperlipidemias"[mh] OR hyperlipid*[tw] OR hyperlipidemia*[tw] OR hyperlipidaemia*[tw] OR hypercholesterol*[tw] OR hypercholesterolemia*[tw] OR hyperlipoproteinemia*[tw] OR hypertriglyceridemia*[tw] OR "arteriosclerosis"[mh] OR "cholesterol"[mh] OR cholesterol[tw] OR “coronary risk factor”[tw] OR "blood pressure"[mh] OR "blood pressure"[tw]
**4**	#1 AND #2 AND #3

Table S2: Search string EMBASE

**#**	**Search terms**
**1**	(coronavir* OR corona virus* OR betacoronavir* OR covid19 OR covid 19 OR nCoV OR CoV 2 OR CoV2 OR sarscov2 OR 2019nCoV OR 2019 novel coronavirus* OR 2019 novel CoV OR wuhan virus*).mp. OR ((wuhan OR hubei OR huanan) AND (severe acute respiratory OR pneumonia*) AND outbreak*).mp.
**2**	social isolation.tw. or physical isolation.tw. or self-isolation.tw. or patient isolation.tw. or exp social isolation/ or exp patient isolation/ or exp isolation/ or exp isolation facility/ or quarantine.tw. or self-quarantine.tw. or exp quarantine/ or social distancing.tw. or physical distancing.tw. or lockdown.tw. or lock down.tw. or shutdown.tw. or shut down.tw. or home confinement.tw. or house arrest.tw. or loneliness.tw. or exp loneliness/
**3**	sedentary.tw. or exp sedentary lifestyle/ or physical inactivity.tw. or exp physical inactivity/ or physical training.tw. or exp training/ or physical activity.tw. or exp physical activity/ or physical lifestyle.tw. or physical education.tw. or exercis*.tw. or exp exercise/ or sport*.tw. or exp sport/ or fitness.tw. or exp fitness/ or exert*.tw. or endur*.tw. or exp endurance/ or aerobic*.tw.
**4**	aliment*.tw. or diet*.tw. or exp diet/ or eat*.tw. or exp eating/ or fare.tw. or feed*.tw. or food*.tw. or exp food/ or nourishment*.tw. or nutrit*.tw. or exp nutrition/
**5**	adipos*.tw. or bodyweight.tw. or body weight.tw or exp body weight/ or obes*.tw. or exp obesity/ or overweight.tw. or weight*.tw.
**6**	tobacco.tw. or exp tobacco/ or smok*.tw. or exp smoking/ or cigarette.tw. or exp cigarette/ or nicotine.tw. or exp nicotine/
**7**	wine*.tw. or liquor*.tw. or spirits.tw. or beer*.tw. or alcohol*.tw. or drink*.tw. or exp drinking behaviour/ or exp alcohol consumption/ or exp alcoholism/ or exp alcoholic beverage/
**8**	exp cardiovascular disease/ or cardio*.tw. or cardia*.tw. or heart*.tw. or coronary*.tw. or angina*.tw. or ventric*.tw. or myocard*.tw. or pericard*.tw. or isch?em*.tw. or emboli*.tw. or arrhythmi*.tw. or thrombo*.tw. or atrial fibrillat*.tw. or tachycardi*.tw. or endocardi*.tw. or (sick adj sinus).tw. or exp cerebrovascular disease/ or (stroke or strokes).tw. or cerebrovasc*.tw. or cerebral vascular.tw. or apoplexy.tw. or (brain adj2 accident*).tw. or ((brain* or cerebral or lacunar) adj2 infarct*).tw. or exp hypertension/ or hypertensi*.tw. or peripheral arter* disease*.tw. or ((high or increased or elevated) adj2 blood pressure).tw. or exp hyperlipidemia/ or hyperlipid*.tw. or hyperlip?emia*.tw. or hypercholesterol*.tw. or hypercholester?emia*.tw. or hyperlipoprotein?emia*.tw. or hypertriglycerid?emia*.tw. or exp Arteriosclerosis/ or exp Cholesterol/ or cholesterol.tw. or "coronary risk factor*".tw. or Blood Pressure/ or blood pressure.tw.
**9**	or/3-8
**10**	1 and 2 and 9

S2 Results

S2.1 Excluded studies

S2.1.1 Studies about patient cohorts (not reported within this paper)

Study	Reference
Ahmed, 2020	Ahmed HO. The impact of social distancing and self-isolation in the last corona COVID-19 outbreak on the body weight in Sulaimani governorate- Kurdistan/Iraq, a prospective case series study. Ann Med Surg (Lond). 2020 Nov;59:110-117. doi: 10.1016/j.amsu.2020.09.024.
Albert et al., 2021	Albert U, Losurdo P, Leschiutta A, Macchi S, Samardzic N, Casaganda B, de Manzini N, Palmisano S. Effect of SARS-CoV-2 (COVID-19) Pandemic and Lockdown on Body Weight, Maladaptive Eating Habits, Anxiety, and Depression in a Bariatric Surgery Waiting List Cohort. Obes Surg. 2021 May;31(5):1905-1911. doi: 10.1007/s11695-021-05257-5.
Berard et al., 2021	Berard M, Rattaz C, Peries M, Loubersac J, Munir K, Baghdadli A. Impact of containment and mitigation measures on children and youth with ASD during the COVID-19 pandemic: Report from the ELENA cohort. J Psychiatr Res. 2021 May;137:73-80. doi: 10.1016/j.jpsychires.2021.02.041.
Biancalana et al., 2021	Biancalana E, Parolini F, Mengozzi A, Solini A. Short-term impact of COVID-19 lockdown on metabolic control of patients with well-controlled type 2 diabetes: a single-centre observational study. Acta Diabetol. 2021 Apr;58(4):431-436. doi: 10.1007/s00592-020-01637-y.
Caruso et al., 2020	Caruso I, Di Molfetta S, Guarini F, Giordano F, Cignarelli A, Natalicchio A, Perrini S, Leonardini A, Giorgino F, Laviola L. Reduction of hypoglycaemia, lifestyle modifications and psychological distress during lockdown following SARS-CoV-2 outbreak in type 1 diabetes. Diabetes Metab Res Rev. 2020 Sep 11:e3404. doi: 10.1002/dmrr.3404.
Chagué et al., 2020	Chagué F, Boulin M, Eicher JC, Bichat F, Saint Jalmes M, Cransac-Miet A, Soudry-Faure A, Danchin N, Cottin Y, Zeller M. Impact of lockdown on patients with congestive heart failure during the coronavirus disease 2019 pandemic. ESC Heart Fail. 2020 Sep 30;7(6):4420–3. doi: 10.1002/ehf2.13016.
Christoforidis et al., 2020	Christoforidis A, Kavoura E, Nemtsa A, Pappa K, Dimitriadou M. Coronavirus lockdown effect on type 1 diabetes management on children wearing insulin pump equipped with continuous glucose monitoring system. Diabetes Res Clin Pract. 2020 Aug;166:108307. doi: 10.1016/j.diabres.2020.108307.
Cirillo et al., 2021	Cirillo M, Rizzello F, Badolato L, De Angelis D, Evangelisti P, Coccia ME, Fatini C. The effects of COVID-19 lockdown on lifestyle and emotional state in women undergoing assisted reproductive technology: Results of an Italian survey. J Gynecol Obstet Hum Reprod. 2021 Feb 2;50(8):102079. doi: 10.1016/j.jogoh.2021.102079.
Cognigi et al., 2021	Cognigni M, D'Agostin M, Schiulaz I, Giangreco M, Carletti C, Faleschini E, Barbi E, Tornese G. HbA1c and BMI after lockdown for COVID-19 in children and adolescents with type 1 diabetes mellitus. Acta Paediatr. 2021 Mar 11. doi: 10.1111/apa.15838.
Cransac-Miet et al., 2021	Cransac-Miet A, Zeller M, Chagué F, Faure AS, Bichat F, Danchin N, Boulin M, Cottin Y. Impact of COVID-19 lockdown on lifestyle adherence in stay-at-home patients with chronic coronary syndromes: Towards a time bomb. Int J Cardiol. 2021 Jan 15;323:285-287. doi: 10.1016/j.ijcard.2020.08.094.
Da Silva et al., 2021	Silva, Jean & Verlengia, Rozangela & Oliveira, José & Ribeiro, Anna & Barbosa, Carolina & Sbeguen Stotzer, Uliana & Crisp, Alex. (2020). Associations between sociodemographic factors and physical activity and sedentary behaviors in adults with chronic diseases during COVID-19 pandemic. Sports Medicine and Health Science. 2. 10.1016/j.smhs.2020.11.002.
De Luis et al., 2020	de Luis Román DA, Izaola O, Primo Martín D, Gómez Hoyos E, Torres Torres B, López Gómez JJ. Effect of lockdown for COVID-19 on self-reported body weight gain in a sample of obese patients. Nutr Hosp. 2020 Dec 16;37(6):1232-1237. English. doi: 10.20960/nh.03307.
De Luis et al., 2021	de Luis D, Izaola O, Primo D, Gómez E, Torres B, Gómez JJL, Pacheco D. Factors Related to Weight Gain in Subjects with Sleeve Gastrectomy During Lockdown by the COVID-19 Pandemic. Obes Surg. 2021 May;31(5):2197-2202. doi: 10.1007/s11695-021-05253-9.
Di Stefano et al., 2021	Di Stefano V, Battaglia G, Giustino V, Gagliardo A, D'Aleo M, Giannini O, Palma A, Brighina F. Significant reduction of physical activity in patients with neuromuscular disease during COVID-19 pandemic: the long-term consequences of quarantine. J Neurol. 2021 Jan;268(1):20-26. doi: 10.1007/s00415-020-10064-6.
Endstrasser et al., 2020	Endstrasser F, Braito M, Linser M, Spicher A, Wagner M, Brunner A. The negative impact of the COVID-19 lockdown on pain and physical function in patients with end-stage hip or knee osteoarthritis. Knee Surg Sports Traumatol Arthrosc. 2020 Aug;28(8):2435-2443. doi: 10.1007/s00167-020-06104-3.
Falcetta et al., 2021	Falcetta P, Aragona M, Ciccarone A, Bertolotto A, Campi F, Coppelli A, Dardano A, Giannarelli R, Bianchi C, Del Prato S. Impact of COVID-19 lockdown on glucose control of elderly people with type 2 diabetes in Italy. Diabetes Res Clin Pract. 2021 Mar 17;174:108750. doi: 10.1016/j.diabres.2021.108750.
Félix et al., 2021	Félix S, de Lourdes M, Ribeiro I, Cunha B, Ramalho S, Vaz AR, Machado PPP, Conceição E. A preliminary study on the psychosocial impact of COVID-19 lockdown in post-bariatric surgery women: the importance of eating behavior, health care access, and social support. Curr Psychol. 2021 Feb 28:1-7. doi: 10.1007/s12144-021-01529-6.
Ghesquière et al., 2021	Ghesquière L, Garabedian C, Drumez E, Lemaître M, Cazaubiel M, Bengler C, Vambergue A. Effects of COVID-19 pandemic lockdown on gestational diabetes mellitus: A retrospective study. Diabetes Metab. 2021 Mar;47(2):101201. doi: 10.1016/j.diabet.2020.09.008.

Study	Reference
Han et al., 2021	Han, Maggie & Preciado, Priscila & Thwin, Ohnmar & Tao, Xia & Tapia-Silva, Leticia & Fuentes, Lemuel & Hakim, Mohamad & Patel, Amrish & Tisdale, Lela & Zhang, Hanjie & Kotanko, Peter. (2021). Effect of Statewide Lockdown in Response to COVID-19 Pandemic on Physical Activity Levels of Hemodialysis Patients. Blood Purification. 1-8. 10.1159/000514935.
Jimenez et al. 2021	Jimenez A, de Hollanda A, Palou E, Ortega E, Andreu A, Molero J, Mestre C, Ibarzabal A, Obach A, Flores L, Cañizares S, Balibrea JM, Vidal J, Escarrabill J, Moize V. Psychoso-cial, Lifestyle, and Body Weight Impact of COVID-19-Related Lockdown in a Sample of Participants with Current or Past History of Obesity in Spain. Obes Surg. 2021 May;31(5):2115-2124. doi: 10.1007/s11695-021-05225-z.
Kang et al., 2021	Kang HM, Jeong DC, Suh BK, Ahn MB. The Impact of the Coronavirus Disease-2019 Pandemic on Childhood Obesity and Vitamin D Status. J Korean Med Sci. 2021 Jan 18;36(3):e21. doi: 10.3346/jkms.2021.36.e21.
Karatas et al., 2021	Karatas S, Yesim T, Beysel S. Impact of lockdown COVID-19 on metabolic control in type 2 diabetes mellitus and healthy people. Prim Care Diabetes. 2021 Jan 9:S1751-9918(21)00003-6. doi: 10.1016/j.pcd.2021.01.003.
Marchitelli et al., 2020	Marchitelli S, Mazza C, Lenzi A, Ricci E, Gnessi L, Roma P. Weight Gain in a Sample of Patients Affected by Overweight/Obesity with and without a Psychiatric Diagnosis during the Covid-19 Lockdown. Nutrients. 2020 Nov 16;12(11):3525. doi: 10.3390/nu12113525.
Mulugeta et al., 2021	Mulugeta, Wudeneh & Hoque, Laboni. (2021). Impact of the COVID-19 lockdown on weight status and associated factors for obesity among Children in Massachusetts. Obesity Medicine. 22. 100325. 10.1016/j.obmed.2021.100325.
Palladino et al., 2020	Palladino F, Merolla E, Solimeno M, de Leva MF, Lenta S, Di Mita O, Bonadies A, Striano P, Tipo V, Varone A. Is Covid-19 lockdown related to an increase of accesses for seizures in the emergency department? An observational analysis of a paediatric cohort in the Southern Italy. Neurol Sci. 2020 Dec;41(12):3475-3483. doi: 10.1007/s10072-020-04824-5.
Pellegrini et al., 2002	Pellegrini M, Ponzo V, Rosato R, Scumaci E, Goitre I, Benso A, Belcastro S, Crespi C, De Michieli F, Ghigo E, Broglio F, Bo S. Changes in Weight and Nutritional Habits in Adults with Obesity during the "Lockdown" Period Caused by the COVID-19 Virus Emergency. Nutrients. 2020 Jul 7;12(7):2016. doi: 10.3390/nu12072016.
Psoma et al., 2020	Psoma O, Papachristoforou E, Kountouri A, Balampanis K, Stergiou A, Lambadiari V, Liatis S, Tsimihodimos V. Effect of COVID-19-associated lockdown on the metabolic control of patients with type 2 diabetes. J Diabetes Complications. 2020 Dec;34(12):107756. doi: 10.1016/j.jdiacomp.2020.107756.
Ruissen et al., 2021	Ruissen MM, Regeer H, Landstra CP, Schroijen M, Jazet I, Nijhoff MF, Pijl H, Ballieux BEPB, Dekkers O, Huisman SD, de Koning EJP. Increased stress, weight gain and less exer-cise in relation to glycemic control in people with type 1 and type 2 diabetes during the COVID-19 pandemic. BMJ Open Diabetes Res Care. 2021 Jan;9(1):e002035. doi: 10.1136/bmjdrc-2020-002035.
Ruiz-Roso et al., 2020	Ruiz-Roso MB, Knott-Torcal C, Matilla-Escalante DC, Garcimartín A, Sampedro-Nuñez MA, Dávalos A, Marazuela M. COVID-19 Lockdown and Changes of the Dietary Pattern and Physical Activity Habits in a Cohort of Patients with Type 2 Diabetes Mellitus. Nutrients. 2020 Aug 4;12(8):2327. doi: 10.3390/nu12082327.
Sanchez-Larsen et al., 2020	Sanchez-Larsen A, Gonzalez-Villar E, Díaz-Maroto I, Layos-Romero A, Martínez-Martín Á, Alcahut-Rodriguez C, Grande-Martin A, Sopelana-Garay D. Influence of the COVID-19 outbreak in people with epilepsy: Analysis of a Spanish population (EPICOVID registry). Epilepsy Behav. 2020 Nov;112:107396. doi: 10.1016/j.yebeh.2020.107396.
Schirinzi et al., 2020	Schirinzi T, Di Lazzaro G, Salimei C, Cerroni R, Liguori C, Scalise S, Alwardat M, Mercuri NB, Pierantozzi M, Stefani A, Pisani A. Physical activity changes and correlate effects in patients with Parkinson's disease during COVID-19 lockdown. Mov Disord Clin Pract. 2020 Jul 17;7(7):797–802. doi: 10.1002/mdc3.13026.
Shah et al., 2020	Shah N, Karguppikar M, Bhor S, Ladkat D, Khadilkar V, Khadilkar A. Impact of lockdown for COVID-19 pandemic in Indian children and youth with type 1 diabetes from different socio-economic classes. J Pediatr Endocrinol Metab. 2020 Nov 13;34(2):217-223. doi: 10.1515/jpem-2020-0460.
Sharma et al., 2020	Sharma P, Ebbert JO, Rosedahl JK, Philpot LM. Changes in substance use among young adults during a respiratory disease pandemic. SAGE Open Med. 2020 Oct 14;8:2050312120965321. doi: 10.1177/2050312120965321.
Suzuki et al., 2020	Suzuki Y, Maeda N, Hirado D, Shirakawa T, Urabe Y. Physical Activity Changes and Its Risk Factors among Community-Dwelling Japanese Older Adults during the COVID-19 Epidemic: Associations with Subjective Well-Being and Health-Related Quality of Life. Int J Environ Res Public Health. 2020 Sep 10;17(18):6591. doi: 10.3390/ijerph17186591.
Tornese et al., 2020	Tornese G, Ceconi V, Monasta L, Carletti C, Faleschini E, Barbi E. Glycemic Control in Type 1 Diabetes Mellitus During COVID-19 Quarantine and the Role of In-Home Physical Activity. Diabetes Technol Ther. 2020 Jun;22(6):462-467. doi: 10.1089/dia.2020.0169.
Veeraiah et al., 2021	Veeraiah S, Sudhakar R, Tripathy JP, Sankar D, Usharani A, Ramakrishnan S, Selvam J, Nagarajan G, Prabhakar DS, Swaminathan R. Tobacco use and quitting behaviour during COVID-19 lockdown. Int J Tuberc Lung Dis. 2021 Mar 1;25(3):247-249. doi: 10.5588/ijtld.20.0728.

S2.1.2 EXCLUDED FULLTEXTS – REASON: TOPICALLY RELEVANT STUDY, BUT USING NON-PROBABILISTIC SAMPLING

Study	Reference
Adams et al., 2020	Adams EL, Caccavale LJ, Smith D, Bean MK. Food Insecurity, the Home Food Environment, and Parent Feeding Practices in the Era of COVID-19. Obesity (Silver Spring). 2020 Nov;28(11):2056-2063. doi: 10.1002/oby.22996.

Study	Reference
Adibelli & Sümen, 2020	Adıbelli D, Sümen A. The effect of the coronavirus (COVID-19) pandemic on health-related quality of life in children. Child Youth Serv Rev. 2020 Dec;119:105595. doi: 10.1016/j.childyouth.2020.105595.
Aegerter et al.,2021	Aegerter AM, Deforth M, Sjøgaard G, Johnston V, Volken T, Luomajoki H, Dratva J, Dressel H, Distler O, Melloh M, Elfering A; NEXpro Collaboration Group. No Evidence for a Decrease in Physical Activity Among Swiss Office Workers During COVID-19: A Longitudinal Study. Front Psychol. 2021 Feb 11;12:620307. doi: 10.3389/fpsyg.2021.620307.
Aguilar-Farias et al., 2020	Aguilar-Farias N, Toledo-Vargas M, Miranda-Marquez S, Cortinez-O'Ryan A, Cristi-Montero C, Rodriguez-Rodriguez F, Martino-Fuentealba P, Okely AD, Del Pozo Cruz B. Socio-demographic Predictors of Changes in Physical Activity, Screen Time, and Sleep among Toddlers and Preschoolers in Chile during the COVID-19 Pandemic. Int J Environ Res Public Health. 2020 Dec 29;18(1):176. doi: 10.3390/ijerph18010176.
Ahmed et al., 2020	Ahmed MZ, Ahmed O, Aibao Z, Hanbin S, Siyu L, Ahmad A. Epidemic of COVID-19 in China and associated Psychological Problems. Asian J Psychiatr. 2020 Jun;51:102092. doi: 10.1016/j.ajp.2020.102092.
Albalawi et al., 2021	Albalawi, A., Hambly, C. and Speakman, J.R. (2021), The impact of the novel coronavirus movement restrictions in the United Kingdom on food outlet usage and body mass index. Obes Sci Pract. https://doi.org/10.1002/osp4.477
Albertella et al., 2021	Albertella L, Rotaru K, Christensen E, Lowe A, Brierley ME, Richardson K, Chamberlain SR, Lee RSC, Kayayan E, Grant JE, Schluter-Hughes S, Ince C, Fontenelle LF, Segrave R, Yücel M. The Influence of Trait Compulsivity and Impulsivity on Addictive and Compulsive Behaviors During COVID-19. Front Psychiatry. 2021 Feb 23;12:634583. doi: 10.3389/fpsyt.2021.634583.
Al-Domi et al., 2021	Al-Domi H, Al-Dalaeen A, Al-Rosan S, Batarseh N, Nawaiseh H. Healthy nutritional behavior during COVID-19 lockdown: A cross-sectional study. Clin Nutr ESPEN. 2021 Apr;42:132-137. doi: 10.1016/j.clnesp.2021.02.003.
Alfawaz et al., 2021	Alfawaz H, Amer OE, Aljumah AA, Aldisi DA, Enani MA, Aljohani NJ, Alotaibi NH, Alshingetti N, Alomar SY, Khattak MNK, Sabico S, Al-Daghri NM. Effects of home quarantine during COVID-19 lockdown on physical activity and dietary habits of adults in Saudi Arabia. Sci Rep. 2021 Mar 15;11(1):5904. doi: 10.1038/s41598-021-85330-2.
Al Hourani et al., 2021	Al Hourani H, Alkhatib B, Abdullah M. Impact of COVID-19 lockdown on body weight, eating habits and physical activity of Jordanian children and adolescents. Disaster Med Public Health Prep. 2021 Feb 16:1-28. doi: 10.1017/dmp.2021.48.
Alhusseini & Alqahtani, 2020	Alhusseini N, Alqahtani A. COVID-19 pandemic's impact on eating habits in Saudi Arabia. J Public Health Res. 2020 Sep 16;9(3):1868. doi: 10.4081/jphr.2020.1868.
Ali et al., 2021	Ali A, Sohaib M, Iqbal S, Hayat K, Khan AU, Rasool MF. Evaluation of COVID-19 Disease Awareness and Its Relation to Mental Health, Dietary Habits, and Physical Activity: A Cross-Sectional Study from Pakistan. Am J Trop Med Hyg. 2021 Mar 9:tpmd201451. doi: 10.4269/ajtmh.20-1451.
Alomari et al., 2020	Alomari MA, Khabour OF, Alzoubi KH. Changes in Physical Activity and Sedentary Behavior Amid Confinement: The BKSQ-COVID-19 Project. Risk Manag Healthc Policy. 2020 Sep 25;13:1757-1764. doi: 10.2147/RMHP.S268320.
Ammar et al., 2020	Ammar A, Brach M, Trabelsi K, Chtourou H, Boukhris O, Masmoudi L, Bouaziz B, Bentlage E, How D, Ahmed M, Müller P, Müller N, Aloui A, Hammouda O, Paineiras-Domingos LL, Braakman-Jansen A, Wrede C, Bastoni S, Pernambuco CS, Mataruna L, Taheri M, Irandoust K, Khacharem A, Bragazzi NL, Chamari K, Glenn JM, Bott NT, Gargouri F, Chaari L, Batatia H, Ali GM, Abdelkarim O, Jarraya M, Abed KE, Souissi N, Van Gemert-Pijnen L, Riemann BL, Riemann L, Moalla W, Gómez-Raja J, Epstein M, Sanderman R, Schulz SV, Jerg A, Al-Horani R, Mansi T, Jmail M, Barbosa F, Ferreira-Santos F, Šimunič B, Pišot R, Gaggioli A, Bailey SJ, Steinacker JM, Driss T, Hoekelmann A. Effects of COVID-19 Home Confinement on Eating Behaviour and Physical Activity: Results of the ECLB-COVID19 International Online Survey. Nutrients. 2020 May 28;12(6):1583. doi: 10.3390/nu12061583.
Androutsos et al., 2021	Androutsos O, Perperidi M, Georgiou C, Chouliaras G. Lifestyle Changes and Determinants of Children's and Adolescents' Body Weight Increase during the First COVID-19 Lock-down in Greece: The COV-EAT Study. Nutrients. 2021 Mar 13;13(3):930. doi: 10.3390/nu13030930.
Antunes et al., 2020	Antunes R, Frontini R, Amaro N, Salvador R, Matos R, Morouço P, Rebelo-Gonçalves R. Exploring Lifestyle Habits, Physical Activity, Anxiety and Basic Psychological Needs in a Sample of Portuguese Adults during COVID-19. Int J Environ Res Public Health. 2020 Jun 18;17(12):4360. doi: 10.3390/ijerph17124360.
Arend et al., 2021	Arend AK, Blechert J, Pannicke B, Reichenberger J. Increased Screen Use on Days With Increased Perceived COVID-19-Related Confinements-A Day Level Ecological Momentary Assessment Study. Front Public Health. 2021 Feb 2;8:623205. doi: 10.3389/fpubh.2020.623205.
Asiamah et al., 2021	Asiamah N, Opuni FF, Mends-Brew E, Mensah SW, Mensah HK, Quansah F. Short-Term Changes in Behaviors Resulting from COVID-19-Related Social Isolation and Their Influ-ences on Mental Health in Ghana. Community Ment Health J. 2021 Jan;57(1):79-92. doi: 10.1007/s10597-020-00722-4.
Assaloni et al., 2020	Assaloni R, Pellino VC, Puci MV, Ferraro OE, Lovecchio N, Girelli A, Vandoni M. Coronavirus disease (Covid-19): How does the exercise practice in active people with type 1 diabe-tes change? A preliminary survey. Diabetes Res Clin Pract. 2020 Aug;166:108297. doi: 10.1016/j.diabres.2020.108297.
Badellino et al., 2021	Badellino H, Gobbo ME, Torres E, Aschieri ME, Biotti M, Alvarez V, Gigante C, Cachiarelli M. 'It's the economy, stupid': Lessons of a longitudinal study of depression in Argentina. Int J Soc Psychiatry. 2021 Mar 11:20764021999687. doi: 10.1177/0020764021999687.
Barone Gibbs et al., 2021	Barone Gibbs B, Kline CE, Huber KA, Paley JL, Perera S. Covid-19 shelter-at-home and work, lifestyle and well-being in desk workers. Occup Med (Lond). 2021 Apr 9;71(2):86-94. doi: 10.1093/occmed/kqab011.
Bartoszek et al., 2020	Bartoszek A, Walkowiak D, Bartoszek A, Kardas G. Mental Well-Being (Depression, Loneliness, Insomnia, Daily Life Fatigue) during COVID-19 Related Home-Confinement-A Study from Poland. Int J Environ Res Public Health. 2020 Oct 12;17(20):7417. doi: 10.3390/ijerph17207417.

Study	Reference
Barwais, 2020	Barwais, Faisal. (2020). Physical Activity at Home During the COVID-19 Pandemic in the Two Most-affected Cities in Saudi Arabia. The Open Public Health Journal. 13. 470-476. 10.2174/1874944502013010470.
Bar-Zeev et al., 2021	Bar-Zeev Y, Shauly M, Lee H, Neumark Y. Changes in Smoking Behaviour and Home-Smoking Rules during the Initial COVID-19 Lockdown Period in Israel. Int J Environ Res Public Health. 2021 Feb 17;18(4):1931. doi: 10.3390/ijerph18041931.
Belgen Kaygısız et al., 2020	Belgen Kaygısız B, Güçhan Topcu Z, Meriç A, Gözgen H, Çoban F. Determination of exercise habits, physical activity level and anxiety level of postmenopausal women during COVID-19 pandemic. Health Care Women Int. 2020 Nov-Dec;41(11-12):1240-1254. doi: 10.1080/07399332.2020.1842878.
Bertrand et al., 2021	Bertrand L, Shaw KA, Ko J, Deprez D, Chilibeck PD, Zello GA. The impact of the coronavirus disease 2019 (COVID-19) pandemic on university students' dietary intake, physical activity, and sedentary behaviour. Appl Physiol Nutr Metab. 2021 Mar;46(3):265-272. doi: 10.1139/apnm-2020-0990.
Bhutani et al., 2021	Bhutani S, vanDellen MR, Cooper JA. Longitudinal Weight Gain and Related Risk Behaviors during the COVID-19 Pandemic in Adults in the US. Nutrients. 2021 Feb 19;13(2):671.
Bin Zarah et al., 2020	Bin Zarah A, Enriquez-Marulanda J, Andrade JM. Relationship between Dietary Habits, Food Attitudes and Food Security Status among Adults Living within the United States Three Months Post-Mandated Quarantine: A Cross-Sectional Study. Nutrients. 2020 Nov 12;12(11):3468. doi: 10.3390/nu12113468.
Biviá-Roig et al.,2020	Biviá-Roig G, La Rosa VL, Gómez-Tébar M, Serrano-Raya L, Amer-Cuenca JJ, Caruso S, Commodari E, Barrasa-Shaw A, Lisón JF. Analysis of the Impact of the Confinement Resulting from COVID-19 on the Lifestyle and Psychological Wellbeing of Spanish Pregnant Women: An Internet-Based Cross-Sectional Survey. Int J Environ Res Public Health. 2020 Aug 15;17(16):5933. doi: 10.3390/ijerph17165933.
Błaszczyk-Bębenek et al., 2020	Błaszczyk-Bębenek E, Jagielski P, Bolesławska I, Jagielska A, Nitsch-Osuch A, Kawalec P. Nutrition Behaviors in Polish Adults before and during COVID-19 Lockdown. Nutrients. 2020 Oct 10;12(10):3084. doi: 10.3390/nu12103084.
Bollen et al., 2021	Bollen Z, Pabst A, Creupelandt C, Fontesse S, Lannoy S, Pinon N, Maurage P. Prior drinking motives predict alcohol consumption during the COVID-19 lockdown: A cross-sectional online survey among Belgian college students. Addict Behav. 2021 Apr;115:106772. doi: 10.1016/j.addbeh.2020.106772.
Bonaccio et al., 2021	Bonaccio M, Costanzo S, Ruggiero E, Persichillo M, Esposito S, Olivieri M, Di Castelnuovo A, Cerletti C, Donati MB, de Gaetano G, Iacoviello L; Moli-LOCK Study Investigators. Changes in ultra-processed food consumption during the first Italian lockdown following the COVID-19 pandemic and major correlates: results from two population-based cohorts. Public Health Nutr. 2021 Mar 5:1-27. doi: 10.1017/S1368980021000999.
Boschuetz et al., 2020	Boschuetz N, Cheng S, Mei L, Loy VM. Changes in Alcohol Use Patterns in the United States During COVID-19 Pandemic. WMJ. 2020 Sep;119(3):171-176.
Botero et al., 2021	Botero JP, Farah BQ, Correia MA, Lofrano-Prado MC, Cucato GG, Shumate G, Ritti-Dias RM, Prado WLD. Impact of the COVID-19 pandemic stay at home order and social isola-tion on physical activity levels and sedentary behavior in Brazilian adults. Einstein (Sao Paulo). 2021 Mar 5;19:eAE6156. doi: 10.31744/einstein_journal/2021AE6156.
Bourdas et al., 2020	Bourdas DI, Zacharakis ED. Evolution of changes in physical activity over lockdown time: Physical activity datasets of four independent adult sample groups corresponding to each of the last four of the six COVID-19 lockdown weeks in Greece. Data Brief. 2020 Oct;32:106301. doi: 10.1016/j.dib.2020.106301.
Bourdas & Zacha-rakis, 2020	Bourdas DI, Zacharakis ED. Impact of COVID-19 Lockdown on Physical Activity in a Sample of Greek Adults. Sports (Basel). 2020 Oct 21;8(10):139. doi: 10.3390/sports8100139.
Brady et al., 2021	Brady SM, Fenton SAM, Metsios GS, Bosworth A, Duda JL, Kitas GD, Veldhuijzen van Zanten JJCS. Different types of physical activity are positively associated with indicators of mental health and psychological wellbeing in rheumatoid arthritis during COVID-19. Rheumatol Int. 2021 Feb;41(2):335-344. doi: 10.1007/s00296-020-04751-w.
Brand et al., 2020	Brand R, Timme S, Nosrat S. When Pandemic Hits: Exercise Frequency and Subjective Well-Being During COVID-19 Pandemic. Front Psychol. 2020 Sep 24;11:570567. doi: 10.3389/fpsyg.2020.570567.
Brindal et al., 2021	Brindal E, Ryan JC, Kakoschke N, Golley S, Zajac IT, Wiggins B. Individual differences and changes in lifestyle behaviours predict decreased subjective well-being during COVID-19 restrictions in an Australian sample. J Public Health (Oxf). 2021 Mar 4:fdab040. doi: 10.1093/pubmed/fdab040.
Brown et al., 2021	Brown L, Mossabir R, Harrison N, Brundle C, Smith J, Clegg A. Life in lockdown: a telephone survey to investigate the impact of COVID-19 lockdown measures on the lives of older people (≥75 years). Age Ageing. 2021 Feb 26;50(2):341-346. doi: 10.1093/ageing/afaa255.
Buoite Stella et al., 2021	Buoite Stella A, AjČeviĆ M, Furlanis G, Cillotto T, Menichelli A, Accardo A, Manganotti P. Smart technology for physical activity and health assessment during COVID-19 lockdown. J Sports Med Phys Fitness. 2021 Mar;61(3):452-460. doi: 10.23736/S0022-4707.20.11373-2.
Cacioppo et al., 2020	Cacioppo M, Bouvier S, Bailly R, Houx L, Lempereur M, Mensah-Gourmel J, Kandalaft C, Varengue R, Chatelin A, Vagnoni J, Vuillerot C, Gautheron V, Dinomais M, Dheilly E, Brochard S, Pons C; ECHO Group. Emerging health challenges for children with physical disabilities and their parents during the COVID-19 pandemic: The ECHO French survey. Ann Phys Rehabil Med. 2020 Aug 18:101429. doi: 10.1016/j.rehab.2020.08.001.
Callinan et al., 2021	Callinan S, Mojica-Perez Y, Wright CJC, Livingston M, Kuntsche S, Laslett AM, Room R, Kuntsche E. Purchasing, consumption, demographic and socioeconomic variables associ-ated with shifts in alcohol consumption during the COVID-19 pandemic. Drug Alcohol Rev. 2021 Feb;40(2):183-191. doi: 10.1111/dar.13200.
Callinan et al., 2020	Callinan S, Smit K, Mojica-Perez Y, D'Aquino S, Moore D, Kuntsche E. Shifts in alcohol consumption during the COVID-19 pandemic: early indications from Australia. Addiction. 2020 Oct 2:10.1111/add.15275. doi: 10.1111/add.15275.

Study	Reference
Callow et al., 2020	Callow DD, Arnold-Nedimala NA, Jordan LS, Pena GS, Won J, Woodard JL, Smith JC. The Mental Health Benefits of Physical Activity in Older Adults Survive the COVID-19 Pandemic. Am J Geriatr Psychiatry. 2020 Oct;28(10):1046-1057. doi: 10.1016/j.jagp.2020.06.024.
Cancello et al., 2020	Cancello R, Soranna D, Zambra G, Zambon A, Invitti C. Determinants of the Lifestyle Changes during COVID-19 Pandemic in the Residents of Northern Italy. Int J Environ Res Public Health. 2020 Aug 28;17(17):6287. doi: 10.3390/ijerph17176287.
Caponnetto et al., 2020	Caponnetto P, Inguscio L, Saitta C, Maglia M, Benfatto F, Polosa R. Smoking behavior and psychological dynamics during COVID-19 social distancing and stay-at-home policies: A survey. Health Psychol Res. 2020 May 27;8(1):9124. doi: 10.4081/hpr.2020.9124.
Carriedo et al., 2020	Carriedo A, Cecchini JA, Fernández-Río J, Méndez-Giménez A. Resilience and physical activity in people under home isolation due to COVID-19: A preliminary evaluation. Ment Health Phys Act. 2020 Oct;19:100361. doi: 10.1016/j.mhpa.2020.100361.
Castañeda-Babarro et al., 2020	Castañeda-Babarro A, Arbillaga-Etxarri A, Gutiérrez-Santamaría B, Coca A. Physical Activity Change during COVID-19 Confinement. Int J Environ Res Public Health. 2020 Sep 21;17(18):6878. doi: 10.3390/ijerph17186878.
Castellini et al., 2020	Castellini G, Cassioli E, Rossi E, Innocenti M, Gironi V, Sanfilippo G, Felciai F, Monteleone AM, Ricca V. The impact of COVID-19 epidemic on eating disorders: A longitudinal observation of pre versus post psychopathological features in a sample of patients with eating disorders and a group of healthy controls. Int J Eat Disord. 2020 Nov;53(11):1855-1862. doi: 10.1002/eat.23368.
Cecchini et al., 2021	Cecchini JA, Carriedo A, Fernández-Río J, Méndez-Giménez A, González C, Sánchez-Martínez B, Rodríguez-González P. A longitudinal study on depressive symptoms and physical activity during the Spanish lockdown. Int J Clin Health Psychol. 2021 Jan-Apr;21(1):100200. doi: 10.1016/j.ijchp.2020.09.001.
Cellini et al., 2020	Cellini N, Canale N, Mioni G, Costa S. Changes in sleep pattern, sense of time and digital media use during COVID-19 lockdown in Italy. J Sleep Res. 2020 Aug;29(4):e13074. doi: 10.1111/jsr.13074.
Chambonniere et al., 2021	Chambonniere C, Lambert C, Fearnbach N, Tardieu M, Fillon A, Genin P, Larras B, Melsens P, Bois J, Pereira B, Tremblay A, Thivel D, Duclos M. Effect of the COVID-19 lockdown on physical activity and sedentary behaviors in French children and adolescents: New results from the ONAPS national survey. Eur J Integr Med. 2021 Apr;43:101308. doi: 10.1016/j.eujim.2021.101308.
Chang et al., 2020	Chang YK, Hung CL, Timme S, Nosrat S, Chu CH. Exercise Behavior and Mood during the COVID-19 Pandemic in Taiwan: Lessons for the Future. Int J Environ Res Public Health. 2020 Sep 28;17(19):7092. doi: 10.3390/ijerph17197092.
Cheikh Ismail et al., 2020a	Cheikh Ismail L, Osaili TM, Mohamad MN, Al Marzouqi A, Jarrar AH, Abu Jamous DO, Magriplis E, Ali HI, Al Sabbah H, Hasan H, AlMarzooqi LMR, Stojanovska L, Hashim M, Shaker Obaid RR, Saleh ST, Al Dhaheri AS. Eating Habits and Lifestyle during COVID-19 Lockdown in the United Arab Emirates: A Cross-Sectional Study. Nutrients. 2020 Oct 29;12(11):3314. doi: 10.3390/nu12113314.
Cheikh Ismail et al., 2020b	Cheikh Ismail L, Osaili TM, Mohamad MN, Al Marzouqi A, Jarrar AH, Zampelas A, Habib-Mourad C, Omar Abu Jamous D, Ali HI, Al Sabbah H, Hasan H, AlMarzooqi LMR, Stojanovska L, Hashim M, Shaker Obaid RR, ElFeky S, Saleh ST, Shawar ZAM, Al Dhaheri AS. Assessment of eating habits and lifestyle during the coronavirus 2019 pandemic in the Middle East and North Africa region: a cross-sectional study. Br J Nutr. 2020 Nov 17:1-10. doi: 10.1017/S0007114520004547.
Cheval et al., 2021	Cheval B, Sivaramakrishnan H, Maltagliati S, Fessler L, Forestier C, Sarrazin P, Orsholits D, Chalabaev A, Sander D, Ntoumanis N, Boisgontier MP. Relationships between changes in self-reported physical activity, sedentary behaviour and health during the coronavirus (COVID-19) pandemic in France and Switzerland. J Sports Sci. 2021 Mar;39(6):699-704. doi: 10.1080/02640414.2020.1841396.
Chodkiewicz et al., 2020	Chodkiewicz J, Talarowska M, Miniszewska J, Nawrocka N, Bilinski P. Alcohol Consumption Reported during the COVID-19 Pandemic: The Initial Stage. Int J Environ Res Public Health. 2020 Jun 29;17(13):4677. doi: 10.3390/ijerph17134677.
Chouchou et al., 2021	Chouchou F, Augustini M, Caderby T, Caron N, Turpin NA, Dalleau G. The importance of sleep and physical activity on well-being during COVID-19 lockdown: reunion island as a case study. Sleep Med. 2021 Jan;77:297-301. doi: 10.1016/j.sleep.2020.09.014.
Constandt et al., 2020	Constandt B, Thibaut E, De Bosscher V, Scheerder J, Ricour M, Willem A. Exercising in Times of Lockdown: An Analysis of the Impact of COVID-19 on Levels and Patterns of Exercise among Adults in Belgium. Int J Environ Res Public Health. 2020 Jun 10;17(11):4144. doi: 10.3390/ijerph17114144.
Constant et al., 2020	Constant A, Conserve DF, Gallopel-Morvan K, Raude J. Socio-Cognitive Factors Associated With Lifestyle Changes in Response to the COVID-19 Epidemic in the General Population: Results From a Cross-Sectional Study in France. Front Psychol. 2020 Sep 29;11:579460. doi: 10.3389/fpsyg.2020.579460.
Coulthard et al., 2021	Coulthard H, Sharps M, Cunliffe L, van den Tol A. Eating in the lockdown during the Covid 19 pandemic; self-reported changes in eating behaviour, and associations with BMI, eating style, coping and health anxiety. Appetite. 2021 Jun 1;161:105082. doi: 10.1016/j.appet.2020.105082.
Cummings et al., 2021	Cummings JR, Ackerman JM, Wolfson JA, Gearhardt AN. COVID-19 stress and eating and drinking behaviors in the United States during the early stages of the pandemic. Appetite. 2021 Jul 1;162:105163. doi: 10.1016/j.appet.2021.105163.
Curtis et al., 2021	Curtis RG, Olds T, Ferguson T, Fraysse F, Dumuid D, Esterman A, Hendrie GA, Brown WJ, Lagiseti R, Maher CA. Changes in diet, activity, weight, and wellbeing of parents during COVID-19 lockdown. PLoS One. 2021 Mar 3;16(3):e0248008. doi: 10.1371/journal.pone.0248008.
da Silva et al., 2021	da Silva LF, de Almeida-Neto PF, BulhÕes-Correia A, de Queiros VS, Matos DG, Silva Dantas PM, de Araujo Tinoco Cabral BG. Impact of social isolation on the level of physical activity in young Brazilian athletes caused by COVID-19. J Sports Med Phys Fitness. 2021 Mar 5. doi: 10.23736/S0022-4707.21.12198-X.

Study	Reference
Deschasaux-Tanguy et al., 2021	Deschasaux-Tanguy M, Druesne-Pecollo N, Esseddik Y, de Edelenyi FS, Allès B, Andreeva VA, Baudry J, Charreire H, Deschamps V, Egnell M, Fezeu LK, Galan P, Julia C, Kesse-Guyot E, Latino-Martel P, Oppert JM, Péneau S, Verdot C, Hercberg S, Touvier M. Diet and physical activity during the coronavirus disease 2019 (COVID-19) lockdown (March-May 2020): results from the French NutriNet-Santé cohort study. Am J Clin Nutr. 2021 Apr 6;113(4):924-938. doi: 10.1093/ajcn/nqaa336.
De Sio et al., 2020	De Sio S, Buomprisco G, La Torre G, Lapteva E, Perri R, Greco E, Mucci N, Cedrone F. The impact of COVID-19 on doctors' well-being: results of a web survey during the lockdown in Italy. Eur Rev Med Pharmacol Sci. 2020 Jul;24(14):7869-7879. doi: 10.26355/eurrev_202007_22292.
Di Corrado et al., 2020	Di Corrado D, Magnano P, Muzii B, Coco M, Guarnera M, De Lucia S, Maldonato NM. Effects of social distancing on psychological state and physical activity routines during the COVID-19 pandemic. Sport Sci Health. 2020 Sep 25:1-6. doi: 10.1007/s11332-020-00697-5.
Di Renzo et al., 2020	Di Renzo L, Gualtieri P, Pivari F, Soldati L, Attinà A, Cinelli G, Leggeri C, Caparello G, Barrea L, Scerbo F, Esposito E, De Lorenzo A. Eating habits and lifestyle changes during COVID-19 lockdown: an Italian survey. J Transl Med. 2020 Jun 8;18(1):229. doi: 10.1186/s12967-020-02399-5.
Di Santo et al., 2020	Di Santo SG, Franchini F, Filiputti B, Martone A, Sannino S. The Effects of COVID-19 and Quarantine Measures on the Lifestyles and Mental Health of People Over 60 at Increased Risk of Dementia. Front Psychiatry. 2020 Oct 14;11:578628. doi: 10.3389/fpsyt.2020.578628.
Dumas et al., 2020	Dumas TM, Ellis W, Litt DM. What Does Adolescent Substance Use Look Like During the COVID-19 Pandemic? Examining Changes in Frequency, Social Contexts, and Pandemic-Related Predictors. J Adolesc Health. 2020 Sep;67(3):354-361. doi: 10.1016/j.jadohealth.2020.06.018.
Drieskens et al., 2021	Drieskens S, Berger N, Vandevijvere S, Gisle L, Braekman E, Charafeddine R, De Ridder K, Demarest S. Short-term impact of the COVID-19 confinement measures on health behaviours and weight gain among adults in Belgium. Arch Public Health. 2021 Feb 22;79(1):22. doi: 10.1186/s13690-021-00542-2.
Dunton et al., 2020a	Dunton GF, Do B, Wang SD. Early effects of the COVID-19 pandemic on physical activity and sedentary behavior in children living in the U.S. BMC Public Health. 2020 Sep 4;20(1):1351. doi: 10.1186/s12889-020-09429-3.
Dunton et al., 2020b	Dunton GF, Wang SD, Do B, Courtney J. Early effects of the COVID-19 pandemic on physical activity locations and behaviors in adults living in the United States. Prev Med Rep. 2020 Dec;20:101241. doi: 10.1016/j.pmedr.2020.101241.
Dutta et al., 2020	Koumi Dutta, Ruchira Mukherjee, Devashish Sen & Subhashis Sahu (2020) Effect of COVID-19 lockdown on sleep behavior and screen exposure time: an observational study among Indian school children, Biological Rhythm Research, DOI: 10.1080/09291016.2020.1825284
Eek et al., 2021	Eek F, Larsson C, Wisén A, Ekvall Hansson E. Self-Perceived Changes in Physical Activity and the Relation to Life Satisfaction and Rated Physical Capacity in Swedish Adults during the COVID-19 Pandemic-A Cross Sectional Study. Int J Environ Res Public Health. 2021 Jan 14;18(2):671. doi: 10.3390/ijerph18020671.
Elnaggar et al.,2020	Elnaggar RK, Alqahtani BA, Mahmoud WS, Elfakharany MS. Physical Activity in Adolescents During the Social Distancing Policies of the COVID-19 Pandemic. Asia Pac J Public Health. 2020 Nov;32(8):491-494. doi: 10.1177/1010539520963564.
Elran-Barak et al., 2020	Elran-Barak R, Mozeikov M. One Month into the Reinforcement of Social Distancing due to the COVID-19 Outbreak: Subjective Health, Health Behaviors, and Loneliness among People with Chronic Medical Conditions. Int J Environ Res Public Health. 2020 Jul 27;17(15):5403. doi: 10.3390/ijerph17155403.
Emerson, 2020	Emerson KG. Coping with being cooped up: Social distancing during COVID-19 among 60+ in the United States. Rev Panam Salud Publica. 2020 Jun 29;44:e81. doi: 10.26633/RPSP.2020.81.
Evans et al., 2021	Evans S, Alkan E, Bhangoo JK, Tenenbaum H, Ng-Knight T. Effects of the COVID-19 lockdown on mental health, wellbeing, sleep, and alcohol use in a UK student sample. Psychiatry Res. 2021 Apr;298:113819. doi: 10.1016/j.psychres.2021.113819.
Fallon et al., 2020	Fallon N, Brown C, Twiddy H, et al. Adverse effects of COVID-19-related lockdown on pain, physical activity and psychological well-being in people with chronic pain. British Journal of Pain. November 2020. doi:10.1177/2049463720973703
Faulkner et al., 2021	Faulkner J, O'Brien WJ, McGrane B, Wadsworth D, Batten J, Askew CD, Badenhorst C, Byrd E, Coulter M, Draper N, Elliot C, Fryer S, Hamlin MJ, Jakeman J, Mackintosh KA, McNarry MA, Mitchelmore A, Murphy J, Ryan-Stewart H, Saynor Z, Schaumberg M, Stone K, Stoner L, Stuart B, Lambrick D. Physical activity, mental health and well-being of adults during initial COVID-19 containment strategies: A multi-country cross-sectional analysis. J Sci Med Sport. 2021 Apr;24(4):320-326. doi: 10.1016/j.jsams.2020.11.016.
Fearnbach et al., 2021	Fearnbach SN, Flanagan EW, Höchsmann C, Beyl RA, Altazan AD, Martin CK, Redman LM. Factors Protecting against a Decline in Physical Activity during the COVID-19 Pandemic. Med Sci Sports Exerc. 2021 Jan 15. doi: 10.1249/MSS.0000000000002602.
Ferrante et al., 2020	Ferrante G, Camussi E, Piccinelli C, Senore C, Armaroli P, Ortale A, Garena F, Giordano L. Did social isolation during the SARS-CoV-2 epidemic have an impact on the lifestyles of citizens? Epidemiol Prev. 2020 Sep-Dec;44(5-6 Suppl 2):353-362. English. doi: 10.19191/EP20.5-6.S2.137.
Fernandez-Rio et al., 2020	Fernandez-Rio J, Cecchini JA, Mendez-Gimenez A, Carriedo A. Weight changes during the COVID-19 home confinement. Effects on psychosocial variables. Obes Res Clin Pract. 2020 Jul-Aug;14(4):383-385. doi: 10.1016/j.orcp.2020.07.006.
Feter et al., 2021	Feter N, Caputo EL, Smith EC, Doring IR, Cassuriaga J, Leite JS, Reichert FF, da Silva MC, Coombes JS, Rombaldi AJ. Association between physical activity and subjective memory decline triggered by the COVID-19 pandemic: Findings from the PAMPA cohort. Prev Med. 2021 Apr;145:106415. doi: 10.1016/j.ypmed.2020.106415.
Flanagan et al., 2021	Flanagan EW, Beyl RA, Fearnbach SN, Altazan AD, Martin CK, Redman LM. The Impact of COVID-19 Stay-At-Home Orders on Health Behaviors in Adults. Obesity (Silver Spring). 2021 Feb;29(2):438-445. doi: 10.1002/oby.23066.

Study	Reference
Fong et al., 2020	Fong BYF, Wong MCS, Law VTS, Lo MF, Ng TKC, Yee HHL, Leung TCH, Ho PWT. Relationships between Physical and Social Behavioural Changes and the Mental Status of Homebound Residents in Hong Kong during the COVID-19 Pandemic. Int J Environ Res Public Health. 2020 Sep 12;17(18):6653. doi: 10.3390/ijerph17186653.
Francisco et al., 2020	Francisco R, Pedro M, Delvecchio E, Espada JP, Morales A, Mazzeschi C, Orgilés M. Psychological Symptoms and Behavioral Changes in Children and Adolescents During the Early Phase of COVID-19 Quarantine in Three European Countries. Front Psychiatry. 2020 Dec 3;11:570164. doi: 10.3389/fpsyt.2020.570164.
Franco et al., 2021	Franco I, Bianco A, Bonfiglio C, Sorino P, Mirizzi A, Campanella A, Buongiorno C, Liuzzi R, Osella AR. Decreased levels of physical activity: results from a cross-sectional study in southern Italy during the COVID-19 lockdown. J Sports Med Phys Fitness. 2021 Feb;61(2):294-300. doi: 10.23736/S0022-4707.20.11536-6.
Füzéki et al., 2021	Füzéki E, Schröder J, Carraro N, Merlo L, Reer R, Groneberg DA, Banzer W. Physical Activity during the First COVID-19-Related Lockdown in Italy. Int J Environ Res Public Health. 2021 Mar 3;18(5):2511. doi: 10.3390/ijerph18052511.
Gadermann et al., 2021	Gadermann AC, Thomson KC, Richardson CG, Gagné M, McAuliffe C, Hirani S, Jenkins E. Examining the impacts of the COVID-19 pandemic on family mental health in Canada: findings from a national cross-sectional study. BMJ Open. 2021 Jan 12;11(1):e042871. doi: 10.1136/bmjopen-2020-042871.
Gaiha et al., 2020	Gaiha SM, Lempert LK, Halpern-Felsher B. Underage Youth and Young Adult e-Cigarette Use and Access Before and During the Coronavirus Disease 2019 Pandemic. JAMA Netw Open. 2020 Dec 1;3(12):e2027572. doi: 10.1001/jamanetworkopen.2020.27572.
Gallè et al., 2020a	Gallè F, Sabella EA, Da Molin G, De Giglio O, Caggiano G, Di Onofrio V, Ferracuti S, Montagna MT, Liguori G, Orsi GB, Napoli C. Understanding Knowledge and Behaviors Related to CoViD-19 Epidemic in Italian Undergraduate Students: The EPICO Study. Int J Environ Res Public Health. 2020 May 16;17(10):3481. doi: 10.3390/ijerph17103481.
Gallè et al., 2020b	Gallè F, Sabella EA, Ferracuti S, De Giglio O, Caggiano G, Protano C, Valeriani F, Parisi EA, Valerio G, Liguori G, Montagna MT, Romano Spica V, Da Molin G, Orsi GB, Napoli C. Sedentary Behaviors and Physical Activity of Italian Undergraduate Students during Lockdown at the Time of CoViD-19 Pandemic. Int J Environ Res Public Health. 2020 Aug 25;17(17):6171. doi: 10.3390/ijerph17176171.
Gallego-Gómez et al., 2020	Gallego-Gómez JI, Campillo-Cano M, Carrión-Martínez A, Balanza S, Rodríguez-González-Moro MT, Simonelli-Muñoz AJ, Rivera-Caravaca JM. The COVID-19 Pandemic and Its Impact on Homebound Nursing Students. Int J Environ Res Public Health. 2020 Oct 10;17(20):7383. doi: 10.3390/ijerph17207383.
Gallo et al., 2020	Gallo LA, Gallo TF, Young SL, Moritz KM, Akison LK. The Impact of Isolation Measures Due to COVID-19 on Energy Intake and Physical Activity Levels in Australian University Students. Nutrients. 2020 Jun 23;12(6):1865. doi: 10.3390/nu12061865.
Garcia et al., 2021	Garcia JM, Lawrence S, Brazendale K, Leahy N, Fukuda D. Brief report: The impact of the COVID-19 pandemic on health behaviors in adolescents with Autism Spectrum Disorder. Disabil Health J. 2021 Apr;14(2):101021. doi: 10.1016/j.dhjo.2020.101021.
García-Tascón et al., 2020	García-Tascón M, Sahelices-Pinto C, Mendaña-Cuervo C, Magaz-González AM. The Impact of the COVID-19 Confinement on the Habits of PA Practice According to Gender (Male/Female): Spanish Case. Int J Environ Res Public Health. 2020 Sep 23;17(19):6961. doi: 10.3390/ijerph17196961.
Garnett et al., 2021	Garnett C, Jackson S, Oldham M, Brown J, Steptoe A, Fancourt D. Factors associated with drinking behaviour during COVID-19 social distancing and lockdown among adults in the UK. Drug Alcohol Depend. 2021 Feb 1;219:108461. doi: 10.1016/j.drugalcdep.2020.108461.
Gendall et al., 2021	Gendall P, Hoek J, Stanley J, Jenkins M, Every-Palmer S. Changes in Tobacco Use During the 2020 COVID-19 Lockdown in New Zealand. Nicotine Tob Res. 2021 Jan 30:ntaa257. doi: 10.1093/ntr/ntaa257.
Genin et al., 2021	Genin PM, Lambert C, Larras B, Pereira B, Toussaint JF, Baker JS, Tremblay A, Thivel D, Duclos M. How Did the COVID-19 Confinement Period Affect Our Physical Activity Level and Sedentary Behaviors? Methodology and First Results From the French National ONAPS Survey. J Phys Act Health. 2021 Feb 13;18(3):296-303. doi: 10.1123/jpah.2020-0449.
Giacalone et al., 2020	Giacalone D, Frøst MB, Rodríguez-Pérez C. Reported Changes in Dietary Habits During the COVID-19 Lockdown in the Danish Population: The Danish COVIDiet Study. Front Nutr. 2020 Dec 8;7:592112. doi: 10.3389/fnut.2020.592112.
Gilic et al., 2020	Gilic B, Ostojic L, Corluka M, Volaric T, Sekulic D. Contextualizing Parental/Familial Influence on Physical Activity in Adolescents before and during COVID-19 Pandemic: A Prospective Analysis. Children (Basel). 2020 Sep 3;7(9):125. doi: 10.3390/children7090125.
Gjaka et al., 2021	Gjaka M, Feka K, Bianco A, Tishukaj F, Giustino V, Parroco AM, Palma A, Battaglia G. The Effect of COVID-19 Lockdown Measures on Physical Activity Levels and Sedentary Behaviour in a Relatively Young Population Living in Kosovo. J Clin Med. 2021 Feb 14;10(4):763. doi: 10.3390/jcm10040763.
Gonzalez et al., 2021	Gonzalez M, Epperson AE, Halpern-Felsher B, Halliday DM, Song AV. Smokers Are More Likely to Smoke More after the COVID-19 California Lockdown Order. Int J Environ Res Public Health. 2021 Mar 5;18(5):2582. doi: 10.3390/ijerph18052582.
Górnicka et al., 2020	Górnicka M, Drywień ME, Zielinska MA, Hamułka J. Dietary and Lifestyle Changes During COVID-19 and the Subsequent Lockdowns among Polish Adults: A Cross-Sectional Online Survey PLifeCOVID-19 Study. Nutrients. 2020 Aug 3;12(8):2324. doi: 10.3390/nu12082324.
Hanafi et al., 2021	Hanafi E, Siste K, Limawan AP, Sen LT, Christian H, Murtani BJ, Adrian, Siswidiani LP, Suwartono C. Alcohol- and Cigarette-Use Related Behaviors During Quarantine and Physical Distancing Amid COVID-19 in Indonesia. Front Psychiatry. 2021 Feb 2;12:622917. doi: 10.3389/fpsyt.2021.622917.
Hargreaves et al., 2021	Hargreaves EA, Lee C, Jenkins M, Calverley JR, Hodge K, Houge Mackenzie S. Changes in Physical Activity Pre-, During and Post-lockdown COVID-19 Restrictions in New Zealand and the Explanatory Role of Daily Hassles. Front Psychol. 2021 Feb 25;12:642954. doi: 10.3389/fpsyg.2021.642954.

Study	Reference
Hashem et al., 2020	Hashem, Shaimaa & Refay, Amira & Mostafa, Hend & Kamel, Iman & Sherif, Lobna. (2020). Impact of Coronavirus Disease-19 Lockdown on Egyptian Children and Adolescents: Dietary Pattern Changes Health Risk. Open Access Macedonian Journal of Medical Sciences. 8. 561-569. 10.3889/oamjms.2020.5249.
He et al., 2020	He M, Xian Y, Lv X, He J, Ren Y. Changes in Body Weight, Physical Activity, and Lifestyle During the Semi-lockdown Period After the Outbreak of COVID-19 in China: An Online Survey. Disaster Med Public Health Prep. 2020 Jul 14:1-6. doi: 10.1017/dmp.2020.237.
Herle et al., 2021	Herle M, Smith AD, Bu F, Steptoe A, Fancourt D. Trajectories of eating behavior during COVID-19 lockdown: Longitudinal analyses of 22,374 adults. Clin Nutr ESPEN. 2021 Apr;42:158-165. doi: 10.1016/j.clnesp.2021.01.046.
Herrera-Valenzuela et al., 2020	Herrera-Valenzuela T, Narrea Vargas JJ, Merlo R, Valdés-Badilla PA, Pardo-Tamayo C, Franchini E. Effect of the COVID-19 quarantine on body mass among combat sports athletes. Nutr Hosp. 2020 Dec 16;37(6):1186-1189. English. doi: 10.20960/nh.03207.
Hu et al., 2020	Hu Z, Lin X, Chiwanda Kaminga A, Xu H. Impact of the COVID-19 Epidemic on Lifestyle Behaviors and Their Association With Subjective Well-Being Among the General Population in Mainland China: Cross-Sectional Study. J Med Internet Res. 2020 Aug 25;22(8):e21176. doi: 10.2196/21176.
Huber et al., 2020a	Huber BC, Steffen J, Schlichtiger J, Graupe T, Deuster E, Strouvelle VP, Fischer MR, Massberg S, Brunner S. Alteration of physical activity during COVID-19 pandemic lockdown in young adults. J Transl Med. 2020 Nov 2;18(1):410. doi: 10.1186/s12967-020-02591-7.
Huber et al., 2020b	Huber BC, Steffen J, Schlichtiger J, Brunner S. Altered nutrition behavior during COVID-19 pandemic lockdown in young adults. Eur J Nutr. 2020 Dec 1:1–10. doi: 10.1007/s00394-020-02435-6.
Husain et al., 2020	Husain W, Ashkanani F. Does COVID-19 change dietary habits and lifestyle behaviours in Kuwait: a community-based cross-sectional study. Environ Health Prev Med. 2020 Oct 12;25(1):61. doi: 10.1186/s12199-020-00901-5.
Ingram et al., 2020	Ingram J, Maciejewski G, Hand CJ. Changes in Diet, Sleep, and Physical Activity Are Associated With Differences in Negative Mood During COVID-19 Lockdown. Front Psychol. 2020 Sep 2;11:588604. doi: 10.3389/fpsyg.2020.588604.
Jacob et al., 2021	Jacob L, Smith L, Armstrong NC, Yakkundi A, Barnett Y, Butler L, McDermott DT, Koyanagi A, Shin JI, Meyer J, Firth J, Remes O, López-Sánchez GF, Tully MA. Alcohol use and mental health during COVID-19 lockdown: A cross-sectional study in a sample of UK adults. Drug Alcohol Depend. 2021 Feb 1;219:108488. doi: 10.1016/j.drugalcdep.2020.108488.
Jackson et al., 2021	Jackson SE, Garnett C, Shahab L, Oldham M, Brown J. Association of the COVID-19 lockdown with smoking, drinking and attempts to quit in England: an analysis of 2019-20 data. Addiction. 2021 May;116(5):1233-1244. doi: 10.1111/add.15295.
Janssen et al., 2020	Janssen X, Fleming L, Kirk A, Rollins L, Young D, Grealy M, MacDonald B, Flowers P, Williams L. Changes in Physical Activity, Sitting and Sleep across the COVID-19 National Lockdown Period in Scotland. Int J Environ Res Public Health. 2020 Dec 14;17(24):9362. doi: 10.3390/ijerph17249362.
Jia et al., 2021a	Jia P, Liu L, Xie X, Yuan C, Chen H, Guo B, Zhou J, Yang S. Changes in dietary patterns among youths in China during COVID-19 epidemic: The COVID-19 impact on lifestyle change survey (COINLICS). Appetite. 2021 Mar 1;158:105015. doi: 10.1016/j.appet.2020.105015.
Jia et al., 2021b	Jia P, Zhang L, Yu W, Yu B, Liu M, Zhang D, Yang S. Impact of COVID-19 lockdown on activity patterns and weight status among youths in China: the COVID-19 Impact on Lifestyle Change Survey (COINLICS). Int J Obes (Lond). 2021 Mar;45(3):695-699. doi: 10.1038/s41366-020-00710-4.
Joensen et al., 2020	Joensen LE, Madsen KP, Holm L, Nielsen KA, Rod MH, Petersen AA, Rod NH, Willaing I. Diabetes and COVID-19: psychosocial consequences of the COVID-19 pandemic in people with diabetes in Denmark-what characterizes people with high levels of COVID-19-related worries? Diabet Med. 2020 Jul;37(7):1146-1154. doi: 10.1111/dme.14319.
Karlon et al., 2021	Kalron A, Dolev M, Greenberg-Abrahami M, Menascu S, Frid L, Avrech-Shezifi S, Harari G, Magalashvili D, Achiron A. Physical activity behavior in people with multiple sclerosis during the COVID-19 pandemic in Israel: Results of an online survey. Mult Scler Relat Disord. 2021 Jan;47:102603. doi: 10.1016/j.msard.2020.102603.
Kaushal et al., 2020	Kaushal N, Keith N, Aguiñaga S, Hagger MS. Social Cognition and Socioecological Predictors of Home-Based Physical Activity Intentions, Planning, and Habits during the COVID-19 Pandemic. Behav Sci (Basel). 2020 Aug 31;10(9):133. doi: 10.3390/bs10090133.
Kishimoto et al., 2020	Kishimoto M, Ishikawa T, Odawara M. Behavioral changes in patients with diabetes during the COVID-19 pandemic. Diabetol Int. 2020 Sep 30;12(2):1-5. doi: 10.1007/s13340-020-00467-1.
Knell et al., 2020	Knell G, Robertson MC, Dooley EE, Burford K, Mendez KS. Health Behavior Changes During COVID-19 Pandemic and Subsequent "Stay-at-Home" Orders. Int J Environ Res Public Health. 2020 Aug 28;17(17):6268. doi: 10.3390/ijerph17176268.
Kochhar et al., 2020	Kochhar AS, Bhasin R, Kochhar GK, Dadlani H, Mehta VV, Kaur R, Bhasin CK. Lockdown of 1.3 billion people in India during Covid-19 pandemic: A survey of its impact on mental health. Asian J Psychiatr. 2020 Dec;54:102213. doi: 10.1016/j.ajp.2020.102213.
Koopmann et al., 2020	Koopmann A, Georgiadou E, Kiefer F, Hillemacher T. Did the General Population in Germany Drink More Alcohol during the COVID-19 Pandemic Lockdown? Alcohol Alcohol. 2020 Oct 20;55(6):698-699. doi: 10.1093/alcalc/agaa058.
Kovacs et al., 2021	Kovacs VA, Starc G, Brandes M, Kaj M, Blagus R, Leskošek B, Suesse T, Dinya E, Guinhouya BC, Zito V, Rocha PM, Gonzalez BP, Kontsevaya A, Brzezinski M, Bidiugan R, Kiraly A, Csányi T, Okely AD. Physical activity, screen time and the COVID-19 school closures in Europe - An observational study in 10 countries. Eur J Sport Sci. 2021 Mar 29:1-10. doi: 10.1080/17461391.2021.1897166.

Study	Reference
Kriaucioniene et al., 2020	Kriaucioniene V, Bagdonaviciene L, Rodríguez-Pérez C, Petkeviciene J. Associations between Changes in Health Behaviours and Body Weight during the COVID-19 Quarantine in Lithuania: The Lithuanian COVIDiet Study. Nutrients. 2020 Oct 13;12(10):3119. doi: 10.3390/nu12103119.
Lamarche et al., 2021	Lamarche B, Brassard D, Lapointe A, Laramée C, Kearney M, Côté M, Bélanger-Gravel A, Desroches S, Lemieux S, Plante C. Changes in diet quality and food security among adults during the COVID-19-related early lockdown: results from NutriQuébec. Am J Clin Nutr. 2021 Apr 6;113(4):984-992. doi: 10.1093/ajcn/nqaa363.
León-Zarceño et al., 2021	León-Zarceño E, Moreno-Tenas A, Boix Vilella S, García-Naveira A, Serrano-Rosa MA. Habits and Psychological Factors Associated With Changes in Physical Activity Due to COVID-19 Confinement. Front Psychol. 2021 Feb 17;12:620745. doi: 10.3389/fpsyg.2021.620745.
Lesser & Nienhuis, 2020	Lesser IA, Nienhuis CP. The Impact of COVID-19 on Physical Activity Behavior and Well-Being of Canadians. Int J Environ Res Public Health. 2020 May 31;17(11):3899. doi: 10.3390/ijerph17113899.
Lippke et al., 2021	Lippke S, Fischer MA, Ratz T. Physical Activity, Loneliness, and Meaning of Friendship in Young Individuals - A Mixed-Methods Investigation Prior to and During the COVID-19 Pandemic With Three Cross-Sectional Studies. Front Psychol. 2021 Feb 2;12:617267. doi: 10.3389/fpsyg.2021.617267.
López-Bueno et al., 2020	López-Bueno R, Calatayud J, Casaña J, Casajús JA, Smith L, Tully MA, Andersen LL, López-Sánchez GF. COVID-19 Confinement and Health Risk Behaviors in Spain. Front Psychol. 2020 Jun 4;11:1426. doi: 10.3389/fpsyg.2020.01426.
López-Gil et al., 2021	López-Gil JF, Tremblay MS, Brazo-Sayavera J. Changes in Healthy Behaviors and Meeting 24-h Movement Guidelines in Spanish and Brazilian Preschoolers, Children and Adolescents during the COVID-19 Lockdown. Children (Basel). 2021 Jan 26;8(2):83. doi: 10.3390/children8020083.
López-Moreno et al., 2020	López-Moreno M, López MTI, Miguel M, Garcés-Rimón M. Physical and Psychological Effects Related to Food Habits and Lifestyle Changes Derived from Covid-19 Home Confinement in the Spanish Population. Nutrients. 2020 Nov 10;12(11):3445. doi: 10.3390/nu12113445.
López-Sánchez et al., 2021	López-Sánchez GF, López-Bueno R, Gil-Salmerón A, Zauder R, Skalska M, Jastrzębska J, Jastrzębski Z, Schuch FB, Grabovac I, Tully MA, Smith L. Comparison of physical activity levels in Spanish adults with chronic conditions before and during COVID-19 quarantine. Eur J Public Health. 2021 Feb 1;31(1):161-166. doi: 10.1093/eurpub/ckaa159.
Luciano et al., 2020	Luciano F, Cenacchi V, Vegro V, Pavei G. COVID-19 lockdown: Physical activity, sedentary behaviour and sleep in Italian medicine students. Eur J Sport Sci. 2020 Dec 6:1-10. doi: 10.1080/17461391.2020.1842910.
Maher et al., 2021	Maher JP, Hevel DJ, Reifsteck EJ, Drollette ES. Physical activity is positively associated with college students' positive affect regardless of stressful life events during the COVID-19 pandemic. Psychol Sport Exerc. 2021 Jan;52:101826. doi: 10.1016/j.psychsport.2020.101826.
Malta et al., 2020	Malta DC, Szwarcwald CL, Barros MBA, Gomes CS, Machado ÍE, Souza Júnior PRB, Romero DE, Lima MG, Damacena GN, Pina MF, Freitas MIF, Werneck AO, Silva DRPD, Azevedo LO, Gracie R. The COVID-19 Pandemic and changes in adult Brazilian lifestyles: a cross-sectional study, 2020. Epidemiol Serv Saude. 2020 Sep 25;29(4):e2020407. Portuguese, English. doi: 10.1590/S1679-49742020000400026.
Martinez et al.,2020	Martinez EZ, Silva FM, Morigi TZ, Zucoloto ML, Silva TL, Joaquim AG, Dall'Agnol G, Galdino G, Martinez MOZ, Silva WRD. Physical activity in periods of social distancing due to COVID-19: a cross-sectional survey. Cien Saude Colet. 2020 Oct;25(suppl 2):4157-4168. doi: 10.1590/1413-812320202510.2.27242020.
Martínez-de-Quel et al., 2021	Martínez-de-Quel Ó, Suárez-Iglesias D, López-Flores M, Pérez CA. Physical activity, dietary habits and sleep quality before and during COVID-19 lockdown: A longitudinal study. Appetite. 2021 Mar 1;158:105019. doi: 10.1016/j.appet.2020.105019.
Matvienko-Sikar et al., 2020	Matvienko-Sikar K, Pope J, Cremin A, Carr H, Leitao S, Olander EK, Meaney S. Differences in levels of stress, social support, health behaviours, and stress-reduction strategies for women pregnant before and during the COVID-19 pandemic, and based on phases of pandemic restrictions, in Ireland. Women Birth. 2020 Oct 23:S1871-5192(20)30367-X. doi: 10.1016/j.wombi.2020.10.010.
Maugeri et al., 2020	Maugeri G, Castrogiovanni P, Battaglia G, Pippi R, D'Agata V, Palma A, Di Rosa M, Musumeci G. The impact of physical activity on psychological health during Covid-19 pandemic in Italy. Heliyon. 2020 Jun 24;6(6):e04315. doi: 10.1016/j.heliyon.2020.e04315.
McPhee et al., 2020	McPhee MD, Keough MT, Rundle S, Heath LM, Wardell JD, Hendershot CS. Depression, Environmental Reward, Coping Motives and Alcohol Consumption During the COVID-19 Pandemic. Front Psychiatry. 2020 Oct 30;11:574676. doi: 10.3389/fpsyt.2020.574676.
Meiring et al., 2021	Meiring RM, Gusso S, McCullough E, Bradnam L. The Effect of the COVID-19 Pandemic Movement Restrictions on Self-Reported Physical Activity and Health in New Zealand: A Cross-Sectional Survey. Int J Environ Res Public Health. 2021 Feb 10;18(4):1719. doi: 10.3390/ijerph18041719.
Meyer et al., 2020	Meyer J, McDowell C, Lansing J, Brower C, Smith L, Tully M, Herring M. Changes in Physical Activity and Sedentary Behavior in Response to COVID-19 and Their Associations with Mental Health in 3052 US Adults. Int J Environ Res Public Health. 2020 Sep 5;17(18):6469. doi: 10.3390/ijerph17186469.
Micheletti et al., 2021	Micheletti Cremasco M, Mulasso A, Moroni A, Testa A, Degan R, Rainoldi A, Rabaglietti E. Relation among Perceived Weight Change, Sedentary Activities and Sleep Quality during COVID-19 Lockdown: A Study in an Academic Community in Northern Italy. Int J Environ Res Public Health. 2021
Mititelu, 2021	Magdalena, Mititelu. (2021). THE IMPACT OF COVID-19 LOCKDOWN ON THE LIFESTYLE AND DIETARY PATTERNS AMONG ROMANIAN POPULATION. FARMACIA. 69. 1-11. 10.31925/farmacia.2021.1.1.
Mon-López et al., 2020a	Mon-López D, Bernardez-Vilaboa R, Fernandez-Balbuena AA, Sillero-Quintana M. The Influence of COVID-19 Isolation on Physical Activity Habits and Its Relationship with Convergence Insufficiency. Int J Environ Res Public Health. 2020 Oct 12;17(20):7406. doi: 10.3390/ijerph17207406.

Study	Reference
Mon-López et al., 2020b	Mon-López D, García-Aliaga A, Ginés Bartolomé A, Muriarte Solana D. How has COVID-19 modified training and mood in professional and non-professional football players? Physiol Behav. 2020 Dec 1;227:113148. doi: 10.1016/j.physbeh.2020.113148.
Mon-López et al., 2020c	Mon-López D, de la Rubia Riaza A, Hontoria Galán M, Refoyo Roman I. The Impact of Covid-19 and the Effect of Psychological Factors on Training Conditions of Handball Players. Int J Environ Res Public Health. 2020 Sep 5;17(18):6471. doi: 10.3390/ijerph17186471.
Monteiro et al., 2021	Monteiro R, Rocha NB, Fernandes S. Are Emotional and Behavioral Problems of Infants and Children Aged Younger Than 7 Years Related to Screen Time Exposure During the Coronavirus Disease 2019 Confinement? An Exploratory Study in Portugal. Front Psychol. 2021 Feb 26;12:590279. doi: 10.3389/fpsyg.2021.590279.
Moore et al., 2020	Moore SA, Faulkner G, Rhodes RE, Brussoni M, Chulak-Bozzer T, Ferguson LJ, Mitra R, O'Reilly N, Spence JC, Vanderloo LM, Tremblay MS. Impact of the COVID-19 virus outbreak on movement and play behaviours of Canadian children and youth: a national survey. Int J Behav Nutr Phys Act. 2020 Jul 6;17(1):85. doi: 10.1186/s12966-020-00987-8.
Munasinghe et al., 2020	Munasinghe S, Sperandei S, Freebairn L, Conroy E, Jani H, Marjanovic S, Page A. The Impact of Physical Distancing Policies During the COVID-19 Pandemic on Health and Well-Being Among Australian Adolescents. J Adolesc Health. 2020 Nov;67(5):653-661. doi: 10.1016/j.jadohealth.2020.08.008.
Murphy et al., 2020	Murphy B, Benson T, McCloat A, Mooney E, Elliott C, Dean M, Lavelle F. Changes in Consumers' Food Practices during the COVID-19 Lockdown, Implications for Diet Quality and the Food System: A Cross-Continental Comparison. Nutrients. 2020 Dec 23;13(1):20. doi: 10.3390/nu13010020.
Nathan et al., 2021	Nathan A, George P, Ng M, Wenden E, Bai P, Phiri Z, Christian H. Impact of COVID-19 Restrictions on Western Australian Children's Physical Activity and Screen Time. Int J Environ Res Public Health. 2021 Mar 5;18(5):2583. doi: 10.3390/ijerph18052583.
Naugthon et al., 2021	Naughton F, Ward E, Khondoker M, Belderson P, Marie Minihane A, Dainty J, Hanson S, Holland R, Brown T, Notley C. Health behaviour change during the UK COVID-19 lockdown: Findings from the first wave of the C-19 health behaviour and well-being daily tracker study. Br J Health Psychol. 2021 May;26(2):624-643. doi: 10.1111/bjhp.12500.
Neill et al., 2020	Neill E, Meyer D, Toh WL, van Rheenen TE, Phillipou A, Tan EJ, Rossell SL. Alcohol use in Australia during the early days of the COVID-19 pandemic: Initial results from the COLLATE project. Psychiatry Clin Neurosci. 2020 Oct;74(10):542-549. doi: 10.1111/pcn.13099.
Niela-Vilén et al., 2021	Niela-Vilén H, Auxier J, Ekholm E, Sarhaddi F, Asgari Mehrabadi M, Mahmoudzadeh A, Azimi I, Liljeberg P, Rahmani AM, Axelin A. Pregnant women's daily patterns of well-being before and during the COVID-19 pandemic in Finland: Longitudinal monitoring through smartwatch technology. PLoS One. 2021 Feb 3;16(2):e0246494. doi: 10.1371/journal.pone.0246494.
Nienhuis et al., 2020	Nienhuis CP, Lesser IA. The Impact of COVID-19 on Women's Physical Activity Behavior and Mental Well-Being. Int J Environ Res Public Health. 2020 Dec 4;17(23):9036. doi: 10.3390/ijerph17239036.
Nieto et al., 2020	Nieto R, Pardo R, Sora B, Feliu-Soler A, Luciano JV. Impact of COVID-19 Lockdown Measures on Spanish People with Chronic Pain: An Online Study Survey. J Clin Med. 2020 Nov 5;9(11):3558. doi: 10.3390/jcm9113558.
Oldham et al., 2021	Oldham M, Garnett C, Brown J, Kale D, Shahab L, Herbec A. Characterising the patterns of and factors associated with increased alcohol consumption since COVID-19 in a UK sample. Drug Alcohol Rev. 2021 Mar 3:10.1111/dar.13256. doi: 10.1111/dar.13256.
Ong et al., 2021	Ong JL, Lau T, Massar SAA, Chong ZT, Ng BKL, Koek D, Zhao W, Yeo BTT, Cheong K, Chee MWL. COVID-19-related mobility reduction: heterogenous effects on sleep and physical activity rhythms. Sleep. 2021 Feb 12;44(2):zsaa179. doi: 10.1093/sleep/zsaa179.
Panagiotidis et al., 2020	Panagiotidis P, Rantis K, Holeva V, Parlapani E, Diakogiannis I. Changes in Alcohol Use Habits in the General Population, during the COVID-19 Lockdown in Greece. Alcohol Alcohol. 2020 Oct 20;55(6):702-704. doi: 10.1093/alcalc/agaa092.
Papandreou et al., 2020	Papandreou C, Arija V, Aretouli E, Tsilidis KK, Bulló M. Comparing eating behaviours, and symptoms of depression and anxiety between Spain and Greece during the COVID-19 outbreak: Cross-sectional analysis of two different confinement strategies. Eur Eat Disord Rev. 2020 Nov;28(6):836-846. doi: 10.1002/erv.2772.
Pabst et al., 2021	Pabst A, Bollen Z, Creupelandt C, Fontesse S, Maurage P. Alcohol consumption changes following COVID-19 lockdown among French-speaking Belgian individuals at risk for alcohol use disorder. Prog Neuropsychopharmacol Biol Psychiatry. 2021 Feb 12;110:110282. doi: 10.1016/j.pnpbp.2021.110282.
Pellegrini et al., 2020	Pellegrini CA, Webster J, Hahn KR, Leblond TL, Unick JL. Relationship between stress and weight management behaviors during the COVID-19 pandemic among those enrolled in an internet program. Obes Sci Pract. 2020 Nov 1;7(1):129-134. doi: 10.1002/osp4.465.
Pérez et al., 2021	Pérez LM, Castellano-Tejedor C, Cesari M, Soto-Bagaria L, Ars J, Zambom-Ferraresi F, Baró S, Díaz-Gallego F, Vilaró J, Enfedaque MB, Espí-Valbé P, Inzitari M. Depressive Symptoms, Fatigue and Social Relationships Influenced Physical Activity in Frail Older Community-Dwellers during the Spanish Lockdown due to the COVID-19 Pandemic. Int J Environ Res Public Health. 2021 Jan 19;18(2):808. doi: 10.3390/ijerph18020808.
Pérez-Rodrigo et al., 2021	Pérez-Rodrigo C, Gianzo Citores M, Hervás Bárbara G, Ruiz-Litago F, Casis Sáenz L, Arija V, López-Sobaler AM, Martínez de Victoria E, Ortega RM, Partearroyo T, Quiles-Izquierdo J, Ribas-Barba L, Rodríguez-Martín A, Salvador Castell G, Tur JA, Varela-Moreiras G, Serra-Majem L, Aranceta-Bartrina J. Patterns of Change in Dietary Habits and Physical Activity during Lockdown in Spain Due to the COVID-19 Pandemic. Nutrients. 2021 Jan 21;13(2):300. doi: 10.3390/nu13020300.
Philippe et al., 2021	Philippe K, Chabanet C, Issanchou S, Monnery-Patris S. Child eating behaviors, parental feeding practices and food shopping motivations during the COVID-19 lockdown in France: (How) did they change? Appetite. 2021 Jun 1;161:105132. doi: 10.1016/j.appet.2021.105132.
Phillipou et al., 2020	Phillipou A, Meyer D, Neill E, Tan EJ, Toh WL, Van Rheenen TE, Rossell SL. Eating and exercise behaviors in eating disorders and the general population during the COVID-19 pandemic in Australia: Initial results from the COLLATE project. Int J Eat Disord. 2020 Jul;53(7):1158-1165. doi: 10.1002/eat.23317.

Study	Reference
Picco et al., 2020	PICCO, J., GONZáLEZ DáVILA, E., GONZáLEZ DáVILA, E., WOLFF, S., WOLFF, S., GóMEZ, V., GóMEZ, V., WOLFF, D., WOLFF, D.. Psychosocial aspects of the COVID-19 pandemic in the Mendoza population. Argentine Journal of Cardiology, North America, 88, may. 2020.
Pirutinsky et al., 2021	Pirutinsky S, Cherniak AD, Rosmarin DH. COVID-19, Religious Coping, and Weight Change in the Orthodox Jewish Community. J Relig Health. 2021 Apr;60(2):646-653. doi: 10.1007/s10943-021-01196-8.
Pišot et al., 2020	Pišot S, Milovanović I, Šimunič B, Gentile A, Bosnar K, Prot F, Bianco A, Lo Coco G, Bartoluci S, Katović D, Bakalár P, Kovalik Slančová T, Tlučáková L, Casals C, Feka K, Christo-gianni A, Drid P. Maintaining everyday life praxis in the time of COVID-19 pandemic measures (ELP-COVID-19 survey). Eur J Public Health. 2020 Dec 11;30(6):1181-1186. doi: 10.1093/eurpub/ckaa157.
Poelman et al., 2021	Poelman MP, Gillebaart M, Schlinkert C, Dijkstra SC, Derksen E, Mensink F, Hermans RCJ, Aardening P, de Ridder D, de Vet E. Eating behavior and food purchases during the COVID-19 lockdown: A cross-sectional study among adults in the Netherlands. Appetite. 2021 Feb 1;157:105002. doi: 10.1016/j.appet.2020.105002.
Poskute et al., 2021	Poskute AS, Nzesi A, Geliebter A. Changes in food intake during the COVID-19 pandemic in New York City. Appetite. 2021 Mar 3;163:105191. doi: 10.1016/j.appet.2021.105191.
Puccinelli et al., 2021	Puccinelli PJ, Costa TS, Seffrin A, de Lira CAB, Vancini RL, Knechtle B, Nikolaidis PT, Andrade MS. Physical Activity Levels and Mental Health during the COVID-19 Pandemic: Preliminary Results of a Comparative Study between Convenience Samples from Brazil and Switzerland. Medicina (Kaunas). 2021 Jan 8;57(1):48. doi: 10.3390/medicina57010048.
Puccinelli et al., 2021	Puccinelli PJ, da Costa TS, Seffrin A, de Lira CAB, Vancini RL, Nikolaidis PT, Knechtle B, Rosemann T, Hill L, Andrade MS. Reduced level of physical activity during COVID-19 pandemic is associated with depression and anxiety levels: an internet-based survey. BMC Public Health. 2021 Mar 1;21(1):425. doi: 10.1186/s12889-021-10470-z.
Qi e tal., 2020	Qi M, Li P, Moyle W, Weeks B, Jones C. Physical Activity, Health-Related Quality of Life, and Stress among the Chinese Adult Population during the COVID-19 Pandemic. Int J Environ Res Public Health. 2020 Sep 7;17(18):6494. doi: 10.3390/ijerph17186494.
Qin e tal., 2020	Qin F, Song Y, Nassis GP, Zhao L, Dong Y, Zhao C, Feng Y, Zhao J. Physical Activity, Screen Time, and Emotional Well-Being during the 2019 Novel Coronavirus Outbreak in China. Int J Environ Res Public Health. 2020 Jul 17;17(14):5170. doi: 10.3390/ijerph17145170.
Radtke et al., 2020	Radtke T, Haile SR, Dressel H, Benden C. Recommended shielding against COVID-19 impacts physical activity levels in adults with cystic fibrosis. J Cyst Fibros. 2020 Nov;19(6):875-879. doi: 10.1016/j.jcf.2020.08.013.
Reyes-Olavarría et al., 2020	Reyes-Olavarría D, Latorre-Román PÁ, Guzmán-Guzmán IP, Jerez-Mayorga D, Caamaño-Navarrete F, Delgado-Floody P. Positive and Negative Changes in Food Habits, Physical Activity Patterns, and Weight Status during COVID-19 Confinement: Associated Factors in the Chilean Population. Int J Environ Res Public Health. 2020 Jul 28;17(15):5431. doi: 10.3390/ijerph17155431.
Rezende et al., 2021	Rezende DAN, Pinto AJ, Goessler KF, Nicoletti CF, Sieczkowska SM, Meireles K, Esteves GP, Genario R, Oliveira Júnior GN, Santo MA, de Cleva R, Roschel H, Gualano B. Influ-ence of Adherence to Social Distancing Due to the COVID-19 Pandemic on Physical Activity Level in Post-bariatric Patients. Obes Surg. 2021 Mar;31(3):1372-1375. doi: 10.1007/s11695-020-05044-8.
Rhodes et al., 2020	Rhodes RE, Liu S, Lithopoulos A, Zhang CQ, Garcia-Barrera MA. Correlates of Perceived Physical Activity Transitions during the COVID-19 Pandemic among Canadian Adults. Appl Psychol Health Well Being. 2020 Dec;12(4):1157-1182. doi: 10.1111/aphw.12236.
Richardson et al., 2021	Richardson DL, Duncan MJ, Clarke ND, Myers TD, Tallis J. The influence of COVID-19 measures in the United Kingdom on physical activity levels, perceived physical function and mood in older adults: A survey-based observational study. J Sports Sci. 2021 Apr;39(8):887-899. doi: 10.1080/02640414.2020.1850984.
Roberts et al., 2020	Roberts C, Gill N, Sims S. The Influence of COVID-19 Lockdown Restrictions on Perceived Nutrition Habits in Rugby Union Players. Front Nutr. 2020 Oct 26;7:589737. doi: 10.3389/fnut.2020.589737.
Robinson et al., 2020a	Robinson E, Boyland E, Chisholm A, Harrold J, Maloney NG, Marty L, Mead BR, Noonan R, Hardman CA. Obesity, eating behavior and physical activity during COVID-19 lockdown: A study of UK adults. Appetite. 2021 Jan 1;156:104853. doi: 10.1016/j.appet.2020.104853.
Robinson et al., 2020	Robinson E, Gillespie S, Jones A. Weight-related lifestyle behaviours and the COVID-19 crisis: An online survey study of UK adults during social lockdown. Obes Sci Pract. 2020 Aug 12;6(6):735-740. doi: 10.1002/osp4.442.
Rodríguez-Gonzá-lez et al., 2020	Rodríguez-González R, Facal D, Martínez-Santos AE, Gandoy-Crego M. Psychological, Social and Health-Related Challenges in Spanish Older Adults During the Lockdown of the COVID-19 First Wave. Front Psychiatry. 2020 Dec 3;11:588949. doi: 10.3389/fpsyt.2020.588949.
Rodríguez-Larrad et al., 2021	Rodríguez-Larrad A, Mañas A, Labayen I, González-Gross M, Espin A, Aznar S, Serrano-Sánchez JA, Vera-Garcia FJ, González-Lamuño D, Ara I, Carrasco-Páez L, Castro-Piñero J, Gómez-Cabrera MC, Márquez S, Tur JA, Gusi N, Benito PJ, Moliner-Urdiales D, Ruiz JR, Ortega FB, Jiménez-Pavón D, Casajús JA, Irazusta J. Impact of COVID-19 Confinement on Physical Activity and Sedentary Behaviour in Spanish University Students: Role of Gender. Int J Environ Res Public Health. 2021 Jan 6;18(2):369. doi: 10.3390/ijerph18020369.
Rodríguez-Pérez et al., 2020	Rodríguez-Pérez C, Molina-Montes E, Verardo V, Artacho R, García-Villanova B, Guerra-Hernández EJ, Ruíz-López MD. Changes in Dietary Behaviours during the COVID-19 Outbreak Confinement in the Spanish COVIDiet Study. Nutrients. 2020 Jun 10;12(6):1730. doi: 10.3390/nu12061730.
Rogers et al., 2020	Rogers NT, Waterlow NR, Brindle H, Enria L, Eggo RM, Lees S, Roberts CH. Behavioral Change Towards Reduced Intensity Physical Activity Is Disproportionately Prevalent Among Adults With Serious Health Issues or Self-Perception of High Risk During the UK COVID-19 Lockdown. Front Public Health. 2020 Sep 30;8:575091. doi: 10.3389/fpubh.2020.575091.

Study	Reference
Rogowska et al., 2020	Rogowska AM, Pavlova I, Kuśnierz C, Ochnik D, Bodnar I, Petrytsa P. Does Physical Activity Matter for the Mental Health of University Students during the COVID-19 Pandemic? J Clin Med. 2020 Oct 29;9(11):3494. doi: 10.3390/jcm9113494.
Rolland et al., 2020	Rolland B, Haesebaert F, Zante E, Benyamina A, Haesebaert J, Franck N. Global Changes and Factors of Increase in Caloric/Salty Food Intake, Screen Use, and Substance Use During the Early COVID-19 Containment Phase in the General Population in France: Survey Study. JMIR Public Health Surveill. 2020 Sep 18;6(3):e19630. doi: 10.2196/19630.
Rossinot et al., 2020	Rossinot H, Fantin R, Venne J. Behavioral Changes During COVID-19 Confinement in France: A Web-Based Study. Int J Environ Res Public Health. 2020 Nov 14;17(22):8444. doi: 10.3390/ijerph17228444.
Rowlands et al., 2021	Rowlands AV, Henson JJ, Coull NA, Edwardson CL, Brady E, Hall A, Khunti K, Davies M, Yates T. The impact of COVID-19 restrictions on accelerometer-assessed physical activity and sleep in individuals with type 2 diabetes. Diabet Med. 2021 Mar 1:e14549. doi: 10.1111/dme.14549.
Ruiz-Roso et al., 2020a	Ruiz-Roso MB, de Carvalho Padilha P, Mantilla-Escalante DC, Ulloa N, Brun P, Acevedo-Correa D, Arantes Ferreira Peres W, Martorell M, Aires MT, de Oliveira Cardoso L, Car-rasco-Marín F, Paternina-Sierra K, Rodriguez-Meza JE, Montero PM, Bernabè G, Pauletto A, Taci X, Visioli F, Dávalos A. Covid-19 Confinement and Changes of Adolescent's Dietary Trends in Italy, Spain, Chile, Colombia and Brazil. Nutrients. 2020 Jun 17;12(6):1807. doi: 10.3390/nu12061807.
Ruiz-Roso et al., 2020b	Ruíz-Roso MB, de Carvalho Padilha P, Matilla-Escalante DC, Brun P, Ulloa N, Acevedo-Correa D, Arantes Ferreira Peres W, Martorell M, Rangel Bousquet Carrilho T, de Oliveira Cardoso L, Carrasco-Marín F, Paternina-Sierra K, Lopez de Las Hazas MC, Rodriguez-Meza JE, Villalba-Montero LF, Bernabè G, Pauletto A, Taci X, Cárcamo-Regla R, Martínez JA, Dávalos A. Changes of Physical Activity and Ultra-Processed Food Consumption in Adolescents from Different Countries during Covid-19 Pandemic: An Observational Study. Nutrients. 2020 Jul 30;12(8):2289. doi: 10.3390/nu12082289.
Sá et al., 2020	Sá CDSC, Pombo A, Luz C, Rodrigues LP, Cordovil R. COVID-19 SOCIAL ISOLATION IN BRAZIL: EFFECTS ON THE PHYSICAL ACTIVITY ROUTINE OF FAMILIES WITH CHILDREN. Rev Paul Pediatr. 2020 Nov 11;39:e2020159. doi: 10.1590/1984-0462/2021/39/2020159.
Sallie et al., 2020	Sallie SN, Ritou V, Bowden-Jones H, Voon V. Assessing international alcohol consumption patterns during isolation from the COVID-19 pandemic using an online survey: highlight-ing negative emotionality mechanisms. BMJ Open. 2020 Nov 26;10(11):e044276. doi: 10.1136/bmjopen-2020-044276.
Sánchez et al., 2021	Sánchez E, Lecube A, Bellido D, Monereo S, Malagón MM, Tinahones FJ, On Behalf Of The Spanish Society For The Study Of Obesity. Leading Factors for Weight Gain during COVID-19 Lockdown in a Spanish Population: A Cross-Sectional Study. Nutrients. 2021 Mar 10;13(3):894. doi: 10.3390/nu13030894.
Sánchez-Sánchez et al., 2020	Sánchez-Sánchez E, Ramírez-Vargas G, Avellaneda-López Y, Orellana-Pecino JI, García-Marín E, Díaz-Jimenez J. Eating Habits and Physical Activity of the Spanish Population during the COVID-19 Pandemic Period. Nutrients. 2020 Sep 15;12(9):2826. doi: 10.3390/nu12092826.
Santana et al., 2021	Santana JDM, Milagres MP, Silva Dos Santos C, Brazil JM, Lima ER, Pereira M. Dietary intake of university students during COVID-19 social distancing in the Northeast of Brazil and associated factors. Appetite. 2021 Jul 1;162:105172. doi: 10.1016/j.appet.2021.105172.
Scacchi et al., 2021	Scacchi A, Catozzi D, Boietti E, Bert F, Siliquini R. COVID-19 Lockdown and Self-Perceived Changes of Food Choice, Waste, Impulse Buying and Their Determinants in Italy: QuarantEat, a Cross-Sectional Study. Foods. 2021 Feb 2;10(2):306. doi: 10.3390/foods10020306.
Scarmozzino & Vi-sioli, 2020	Scarmozzino F, Visioli F. Covid-19 and the Subsequent Lockdown Modified Dietary Habits of Almost Half the Population in an Italian Sample. Foods. 2020 May 25;9(5):675. doi: 10.3390/foods9050675.
Schlichtiger et al., 2021	Schlichtiger J, Steffen J, Huber BC, Brunner S. Physical activity during COVID-19 lockdown in older adults. J Sports Med Phys Fitness. 2021 Jan;61(1):164-166. doi: 10.23736/S0022-4707.20.11726-2.
Schmidt et al., 2021	Schmidt T, Pawlowski CS. Physical Activity in Crisis: The Impact of COVID-19 on Danes' Physical Activity Behavior. Front Sports Act Living. 2021 Feb 1;2:610255. doi: 10.3389/fspor.2020.610255.
Schmits & Glowacz, 2021	Schmits E, Glowacz F. Changes in Alcohol Use During the COVID-19 Pandemic: Impact of the Lockdown Conditions and Mental Health Factors. Int J Ment Health Addict. 2021 Jan 4:1-12. doi: 10.1007/s11469-020-00432-8.
Schnitzer et al., 2020	Schnitzer M, Schöttl SE, Kopp M, Barth M. COVID-19 stay-at-home order in Tyrol, Austria: sports and exercise behaviour in change? Public Health. 2020 Aug;185:218-220. doi: 10.1016/j.puhe.2020.06.042.
Sciberras et al., 2020	Sciberras E, Patel P, Stokes MA, Coghill D, Middeldorp CM, Bellgrove MA, Becker SP, Efron D, Stringaris A, Faraone SV, Bellows ST, Quach J, Banaschewski T, McGillivray J, Hutchinson D, Silk TJ, Melvin G, Wood AG, Jackson A, Loram G, Engel L, Montgomery A, Westrupp E. Physical Health, Media Use, and Mental Health in Children and Adolescents With ADHD During the COVID-19 Pandemic in Australia. J Atten Disord. 2020 Dec 17:1087054720978549. doi: 10.1177/1087054720978549.
Shinohara et al., 2021	Shinohara T, Saida K, Tanaka S, Murayama A. Association between frailty and changes in lifestyle and physical or psychological conditions among older adults affected by the coronavirus disease 2019 countermeasures in Japan. Geriatr Gerontol Int. 2021 Jan;21(1):39-42. doi: 10.1111/ggi.14092.
Shrestha et al., 2020	Shrestha, Carmina & Acharya, Sajan & Sharma, Raksha & Khanal, Roja & Joshi, Jasmin & Ghimire, Calvin & Bhandari, Prakriti & Agrawal, Aditi. (2020). Changes and Compromises in Health Choices during COVID-19 Lockdown in Kathmandu Valley: A Descriptive Cross-sectional Study. Journal of Nepal Medical Association. 58. 10.31729/jnma.5790.
Serin & Koç, 2020	Emre Serin, Mustafa Can Koç.Examination of the eating behaviours and depression states of the university students who stay at home during the coronavirus pandemic in terms of different variables. Progress in Nutrition 2020; Vol. 22, Supplement 1: 33-43. DOI: 10.23751/pn.v22i1-S.9780

Study	Reference
Sfendla & Hadrya, 2020	Sfendla A, Hadrya F. Factors Associated with Psychological Distress and Physical Activity During the COVID-19 Pandemic. Health Secur. 2020 Dec;18(6):444-453. doi: 10.1089/hs.2020.0062.
Sidor et al., 2020	Sidor A, Rzymski P. Dietary Choices and Habits during COVID-19 Lockdown: Experience from Poland. Nutrients. 2020 Jun 3;12(6):1657. doi: 10.3390/nu12061657.
Siegle et al., 2020	Siegle CBH, Pombo A, Luz C, Rodrigues LP, Cordovil R, Sá CDSC. INFLUENCES OF FAMILY AND HOUSEHOLD CHARACTERISTICS ON CHILDREN'S LEVEL OF PHYSICAL ACTIVITY DURING SOCIAL DISTANCING DUE TO COVID-19 IN BRAZIL. Rev Paul Pediatr. 2020 Dec 7;39:e2020297. doi: 10.1590/1984-0462/2021/39/2020297.
Silva et al., 2020	Silva LRB, Seguro CS, de Oliveira CGA, Santos POS, de Oliveira JCM, de Souza Filho LFM, de Paula Júnior CA, Gentil P, Rebelo ACS. Physical Inactivity Is Associated With Increased Levels of Anxiety, Depression, and Stress in Brazilians During the COVID-19 Pandemic: A Cross-Sectional Study. Front Psychiatry. 2020 Nov 17;11:565291. doi: 10.3389/fpsyt.2020.565291.
Sinha et al., 2020	Sinha M, Pande B, Sinha R. Impact of COVID-19 lockdown on sleep-wake schedule and associated lifestyle related behavior: A national survey. J Public Health Res. 2020 Jul 28;9(3):1826. doi: 10.4081/jphr.2020.1826.
Solé et al., 2021	Solé B, Verdolini N, Amoretti S, Montejo L, Rosa AR, Hogg B, Garcia-Rizo C, Mezquida G, Bernardo M, Martinez-Aran A, Vieta E, Torrent C. Effects of the COVID-19 pandemic and lockdown in Spain: comparison between community controls and patients with a psychiatric disorder. Preliminary results from the BRIS-MHC STUDY. J Affect Disord. 2021 Feb 15;281:13-23. doi: 10.1016/j.jad.2020.11.099.
Spence et al., 2021	Spence JC, Rhodes RE, McCurdy A, Mangan A, Hopkins D, Mummery WK. Determinants of physical activity among adults in the United Kingdom during the COVID-19 pandemic: The DUK-COVID study. Br J Health Psychol. 2021 May;26(2):588-605. doi: 10.1111/bjhp.12497.
Szajnoga et al., 2020	Szajnoga D, Klimek-Tulwin M, Piekut A. COVID-19 lockdown leads to changes in alcohol consumption patterns. Results from the Polish national survey. J Addict Dis. 2021 Apr-Jun;39(2):215-225. doi: 10.1080/10550887.2020.1848247.
Ten Velde et al., 2021	Ten Velde G, Lubrecht J, Arayess L, van Loo C, Hesselink M, Reijnders D, Vreugdenhil A. Physical activity behaviour and screen time in Dutch children during the COVID-19 pan-demic: Pre-, during- and post-school closures. Pediatr Obes. 2021 Feb 23:e12779. doi: 10.1111/ijpo.12779.
Theis et al., 2021	Theis N, Campbell N, De Leeuw J, Owen M, Schenke KC. The effects of COVID-19 restrictions on physical activity and mental health of children and young adults with physical and/or intellectual disabilities. Disabil Health J. 2021 Jan 22:101064. doi: 10.1016/j.dhjo.2021.101064.
Tulchin-Francis et al., 2021	Tulchin-Francis, Kirsten & Stevens, Wilshaw & Gu, Xiangli & Zhang, Tao & Roberts, Heather & Keller, Jean & Dempsey, Dana & Borchard, Justine & Jeans, Kelly & VanPelt, Jona-than. (2021). The impact of the COVID-19 pandemic on physical activity in US children. Journal of Sport and Health Science. 10.1016/j.jshs.2021.02.005.
Ugbolue et al., 2020	Ugbolue UC, Duclos M, Urzeala C, Berthon M, Kulik K, Bota A, Thivel D, Bagheri R, Gu Y, Baker JS, Andant N, Pereira B, Rouffiac K, Clinchamps M, Dutheil F, On Behalf Of The Covistress Network. An Assessment of the Novel COVISTRESS Questionnaire: COVID-19 Impact on Physical Activity, Sedentary Action and Psychological Emotion. J Clin Med. 2020 Oct 19;9(10):3352. doi: 10.3390/jcm9103352.
Van Bakel et al., 2021	van Bakel BMA, Bakker EA, de Vries F, Thijssen DHJ, Eijsvogels TMH. Impact of COVID-19 lockdown on physical activity and sedentary behaviour in Dutch cardiovascular disease patients. Neth Heart J. 2021 May;29(5):273-279. doi: 10.1007/s12471-021-01550-1.
van der Heide et al., 2020	van der Heide A, Meinders MJ, Bloem BR, Helmich RC. The Impact of the COVID-19 Pandemic on Psychological Distress, Physical Activity, and Symptom Severity in Parkinson's Disease. J Parkinsons Dis. 2020;10(4):1355-1364. doi: 10.3233/JPD-202251.
Van Rheenen et al., 2020	Van Rheenen TE, Meyer D, Neill E, Phillipou A, Tan EJ, Toh WL, Rossell SL. Mental health status of individuals with a mood-disorder during the COVID-19 pandemic in Australia: Initial results from the COLLATE project. J Affect Disord. 2020 Oct 1;275:69-77. doi: 10.1016/j.jad.2020.06.037.
Vanderbruggen et al., 2020	Vanderbruggen N, Matthys F, Van Laere S, Zeeuws D, Santermans L, Van den Ameele S, Crunelle CL. Self-Reported Alcohol, Tobacco, and Cannabis Use during COVID-19 Lock-down Measures: Results from a Web-Based Survey. Eur Addict Res. 2020;26(6):309-315. doi: 10.1159/000510822.
Vetrovsky et al., 2020	Vetrovsky T, Frybova T, Gant I, Semerad M, Cimler R, Bunc V, Siranec M, Miklikova M, Vesely J, Griva M, Precek J, Pelouch R, Parenica J, Belohlavek J. The detrimental effect of COVID-19 nationwide quarantine on accelerometer-assessed physical activity of heart failure patients. ESC Heart Fail. 2020 Oct;7(5):2093-2097. doi: 10.1002/ehf2.12916.
Walędziak et al., 2020	Walędziak M, Różańska-Walędziak A, Pędziwiatr M, Szeliga J, Proczko-Stepaniak M, Wysocki M, Stefura T, Major P. Bariatric Surgery During COVID-19 Pandemic from Patients' Point of View-The Results of a National Survey. J Clin Med. 2020 Jun 2;9(6):1697. doi: 10.3390/jcm9061697.
Wang et al., 2020	Wang X, Lei SM, Le S, Yang Y, Zhang B, Yao W, Gao Z, Cheng S. Bidirectional Influence of the COVID-19 Pandemic Lockdowns on Health Behaviors and Quality of Life among Chinese Adults. Int J Environ Res Public Health. 2020 Aug 2;17(15):5575. doi: 10.3390/ijerph17155575.
Weerakoon et al., 2020	Weerakoon SM, Jetelina KK, Knell G. Longer time spent at home during COVID-19 pandemic is associated with binge drinking among US adults. Am J Drug Alcohol Abuse. 2021 Jan 2;47(1):98-106. doi: 10.1080/00952990.2020.1832508.
Werneck et al., 2021a	Werneck AO, Silva DR, Malta DC, Gomes CS, Souza-Júnior PR, Azevedo LO, Barros MB, Szwarcwald CL. Associations of sedentary behaviours and incidence of unhealthy diet during the COVID-19 quarantine in Brazil. Public Health Nutr. 2021 Feb;24(3):422-426. doi: 10.1017/S1368980020004188.
Werneck et al., 2021b	Werneck AO, Silva DR, Malta DC, Souza-Júnior PRB, Azevedo LO, Barros MBA, Szwarcwald CL. Changes in the clustering of unhealthy movement behaviors during the COVID-19 quarantine and the association with mental health indicators among Brazilian adults. Transl Behav Med. 2021 Mar 16;11(2):323-331. doi: 10.1093/tbm/ibaa095.

Study	Reference
Werneck et al., 2020a	Werneck AO, Silva DR, Malta DC, Souza-Júnior PRB, Azevedo LO, Barros MBA, Szwarcwald CL. Physical inactivity and elevated TV-viewing reported changes during the COVID-19 pandemic are associated with mental health: A survey with 43,995 Brazilian adults. J Psychosom Res. 2021 Jan;140:110292. doi: 10.1016/j.jpsychores.2020.110292. Epub 2020 Nov 10.
Werneck et al., 2020b	Werneck AO, Silva DRD, Malta DC, Souza-Júnior PRB, Azevedo LO, Barros MBA, Szwarcwald CL. Lifestyle behaviors changes during the COVID-19 pandemic quarantine among 6,881 Brazilian adults with depression and 35,143 without depression. Cien Saude Colet. 2020 Oct;25(suppl 2):4151-4156. doi: 10.1590/1413-812320202510.2.27862020.
Wichaidit et al., 2021	Wichaidit W, Sittisombut M, Assanangkornchai S, Vichitkunakorn P. Self-reported drinking behaviors and observed violation of state-mandated social restriction and alcohol control measures during the COVID-19 pandemic: Findings from nationally-representative surveys in Thailand. Drug Alcohol Depend. 2021 Apr 1;221:108607. doi: 10.1016/j.drugalcdep.2021.108607.
Wilke et al., 2021	Wilke J, Mohr L, Tenforde AS, Edouard P, Fossati C, González-Gross M, Sánchez Ramírez C, Laiño F, Tan B, Pillay JD, Pigozzi F, Jimenez-Pavon D, Novak B, Jaunig J, Zhang M, van Poppel M, Heidt C, Willwacher S, Yuki G, Lieberman DE, Vogt L, Verhagen E, Hespanhol L, Hollander K. A Pandemic within the Pandemic? Physical Activity Levels Substantially Decreased in Countries Affected by COVID-19. Int J Environ Res Public Health. 2021 Feb 24;18(5):2235. doi: 10.3390/ijerph18052235.
Wilson et al., 2021	Wilson JJ, McMullan I, Blackburn NE, Klempel N, Yakkundi A, Armstrong NC, Brolly C, Butler LT, Barnett Y, Jacob L, Koyanagi A, Smith L, Tully MA. Changes in dietary fat intake and associations with mental health in a UK public sample during the COVID-19 pandemic. J Public Health (Oxf). 2021 Mar 1:fdab009. doi: 10.1093/pubmed/fdab009.
Winkler et al., 2020	Winkler P, Formanek T, Mlada K, Kagstrom A, Mohrova Z, Mohr P, Csemy L. Increase in prevalence of current mental disorders in the context of COVID-19: analysis of repeated nationwide cross-sectional surveys. Epidemiol Psychiatr Sci. 2020 Sep 29;29:e173. doi: 10.1017/S2045796020000888.
Wood et al., 2021	Wood CJ, Barton J, Smyth N. A cross-sectional study of physical activity behaviour and associations with wellbeing during the UK coronavirus lockdown. J Health Psychol. 2021 Mar 3:1359105321999710. doi: 10.1177/1359105321999710.
Yan et al., 2020	Yan AF, Sun X, Zheng J, Mi B, Zuo H, Ruan G, Hussain A, Wang Y, Shi Z. Perceived risk, behavior changes and Health-related outcomes during COVID-19 pandemic: Findingsamong adults with and without diabetesin China. Diabetes Res Clin Pract. 2020 Sep;167:108350. doi: 10.1016/j.diabres.2020.108350.
Yang et al., 2020	Yang S, Guo B, Ao L, Yang C, Zhang L, Zhou J, Jia P. Obesity and activity patterns before and during COVID-19 lockdown among youths in China. Clin Obes. 2020 Dec;10(6):e12416. doi: 10.1111/cob.12416.
Zach et al., 2021	Zach S, Zeev A, Ophir M, Eilat-Adar S. Physical activity, resilience, emotions, moods, and weight control of older adults during the COVID-19 global crisis. Eur Rev Aging Phys Act. 2021 Mar 1;18(1):5. doi: 10.1186/s11556-021-00258-w.
Zachary et al., 2020	Zachary Z, Brianna F, Brianna L, Garrett P, Jade W, Alyssa D, Mikayla K. Self-quarantine and weight gain related risk factors during the COVID-19 pandemic. Obes Res Clin Pract. 2020 May-Jun;14(3):210-216. doi: 10.1016/j.orcp.2020.05.004.
Zhang et al., 2020	Zhang J, Zhang Y, Huo S, Ma Y, Ke Y, Wang P, Zhao A. Emotional Eating in Pregnant Women during the COVID-19 Pandemic and Its Association with Dietary Intake and Gestational Weight Gain. Nutrients. 2020 Jul 28;12(8):2250. doi: 10.3390/nu12082250.
Zhao et al., 2020	Zhao A, Li Z, Ke Y, Huo S, Ma Y, Zhang Y, Zhang J, Ren Z. Dietary Diversity among Chinese Residents during the COVID-19 Outbreak and Its Associated Factors. Nutrients. 2020 Jun 6;12(6):1699. doi: 10.3390/nu12061699.
Zheng et al., 2020	Zheng C, Huang WY, Sheridan S, Sit CH, Chen XK, Wong SH. COVID-19 Pandemic Brings a Sedentary Lifestyle in Young Adults: A Cross-Sectional and Longitudinal Study. Int J Environ Res Public Health. 2020 Aug 19;17(17):6035. doi: 10.3390/ijerph17176035.
Zhou et al., 2021	Zhou J, Xie X, Guo B, Pei R, Pei X, Yang S, Jia P. Impact of COVID-19 Lockdown on Physical Activity Among the Chinese Youths: The COVID-19 Impact on Lifestyle Change Survey (COINLICS). Front Public Health. 2021 Feb 4;9:592795. doi: 10.3389/fpubh.2021.592795.
Zorcec et al., 2020	Zorcec T, Jakovska T, Micevska V, Boskovska K, Cholakovska VC. Pandemic with COVID-19 and Families with Children with Chronic Respiratory Diseases. Pril (Makedon Akad Nauk Umet Odd Med Nauki). 2020 Sep 1;41(2):95-101. doi: 10.2478/prilozi-2020-0038.

S2.1.3 Excluded Fulltexts – Reason: Study design

Study	Reference
Anonymus, 2021	Corrigendum to: Comparison of physical activity levels in Spanish adults with chronic conditions before and during COVID-19 quarantine. Eur J Public Health. 2021 Mar 13:ckab005. doi: 10.1093/eurpub/ckab005.
Abbas & Kamel, 2020	Abbas AM, Kamel MM. Dietary habits in adults during quarantine in the context of COVID-19 pandemic. Obes Med. 2020 Sep;19:100254. doi: 10.1016/j.obmed.2020.100254.

Study	Reference
Agababian et al., 2020	A. S. Aghababian, K.; Jansen, E.; Thapaliya, G.; Sadler, J.; Baltimore, Md L. C.; Carnell, S. Binge watching during COVID: Associations with stress and body weight. Obesity ; 28:10-10, 2020.
Atesyakar et al., 2020	Atesyakar, Nilay & Ecevitoglu, Alev & Duman, Elif. (2020). Preliminary findings on the impact of COVID-19 pandemic on mood and daily routines of women from the BABIP birth cohort. Psychoneuroendocrinology. 119. 104965. 10.1016/j.psyneuen.2020.104965
Balanzá-Martínez et al., 2020	Balanzá-Martínez V, Atienza-Carbonell B, Kapczinski F, De Boni RB. Lifestyle behaviours during the COVID-19 - time to connect. Acta Psychiatr Scand. 2020 May;141(5):399-400. doi: 10.1111/acps.13177.
Balducci & Coccia, 2021	Balducci S, Coccia EM. Sedentariness and physical activity in type 2 diabetes during the COVID-19 pandemic. Diabetes Metab Res Rev. 2021 Feb;37(2):e3378. doi: 10.1002/dmrr.3378.
Baysun & Akar, 2020	Baysun Ş, Akar MN. WEIGHT GAIN IN CHILDREN DURING THE COVID-19 QUARANTINE PERIOD. J Paediatr Child Health. 2020 Sep;56(9):1487-1488. doi: 10.1111/jpc.15105.
Bian, 2020	Bian Y. Epidemic-specific social capital and its impact on physical activity and health status. J Sport Health Sci. 2020 Sep;9(5):426-429. doi: 10.1016/j.jshs.2020.07.009.
Branley-Bell & Tal-bot, 2020	Branley-Bell D, Talbot CV. Exploring the impact of the COVID-19 pandemic and UK lockdown on individuals with experience of eating disorders. J Eat Disord. 2020 Aug 31;8:44. doi: 10.1186/s40337-020-00319-y.
Brigugino et al., 2020	Briguglio M, Giorgino R, Dell'Osso B, Cesari M, Porta M, Lattanzio F, Banfi G, Peretti GM. Consequences for the Elderly After COVID-19 Isolation: FEaR (Frail Elderly amid Re-strictions). Front Psychol. 2020 Sep 28;11:565052. doi: 10.3389/fpsyg.2020.565052.
Bhutani et al., 2020	S. V. D. Bhutani, M.; Haskins, L. A.; Cooper, J. Psychological/health factors may alter energy balance behaviors during covid-19 home confinement. Obesity; 28:145-145, 2020.
Calceterra et al., 2020	Calcaterra V, Vandoni M, Pellino VC, Cena H. Special Attention to Diet and Physical Activity in Children and Adolescents With Obesity During the Coronavirus Disease-2019 Pan-demic. Front Pediatr. 2020 Jun 26;8:407. doi: 10.3389/fped.2020.00407.
Iho & Gois, 2020	Iho VO, Gois CO. COVID-19 pandemic and home-based physical activity. J Allergy Clin Immunol Pract. 2020 Sep;8(8):2833-2834. doi: 10.1016/j.jaip.2020.05.018.
Cetin et al., 2020	Cetin, S. Y., Buyuk, A., Gencer, G. Y. G. Investigation of leisure time activities and anxiety in elderly of Non-Covid living at home during COVID-19 period. European Geriatric Medi-cine 2020; 11 (SUPPL 1): S96
Chague et al., 2021	Chague, Frederic & Boulin, M. & Eicher, Jean-Christophe & Bichat, Florence & Saint-Jalmes, M. & Cransac, A. & Trojak, B. & Soudry-Faure, Agnès & Danchin, N. & Cottin, Y. & Zeller, Marianne. (2021). Alarming increased rate of smoking and associated lifestyle behaviours in patients with chronic cardiac diseases during COVID-19 pandemic related lock-down. Archives of Cardiovascular Diseases Supplements. 13. 127. 10.1016/j.acvdsp.2020.10.265.
Chaves et al., 2020	Chaves, R. & Correia, L. & Ramalho, R. & Pereira, Paula. (2020). Food habits and body image perception changes during Covid-19 pandemic in Portugal. Clinical Nutrition ESPEN. 40. 620. 10.1016/j.clnesp.2020.09.643.
Chen et al., 2020	Chen F, Zheng D, Liu J, Gong Y, Guan Z, Lou D. Depression and anxiety among adolescents during COVID-19: A cross-sectional study. Brain Behav Immun. 2020 Aug;88:36-38. doi: 10.1016/j.bbi.2020.05.061.
Chen-Sankey et al., 2020	Chen-Sankey JC, Broun A, Duarte DA, Ajith A, Jewett B, Smiley SL, Mead-Morse EL, Guy MC, Choi K. Exploring changes in cigar smoking patterns and motivations to quit cigars among black young adults in the time of COVID-19. Addict Behav Rep. 2020 Nov 12;12:100317. doi: 10.1016/j.abrep.2020.100317.
Clemmensen et al., 2020	Clemmensen C, Petersen MB, Sørensen TIA. Will the COVID-19 pandemic worsen the obesity epidemic? Nat Rev Endocrinol. 2020 Sep;16(9):469-470. doi: 10.1038/s41574-020-0387-z.
Cooper et al., 2021	Cooper JA, vanDellen M, Bhutani S. Self-weighing Practices and Associated Health Behaviors during COVID-19. Am J Health Behav. 2021 Jan 1;45(1):17-30. doi: 10.5993/AJHB.45.1.2.
de la Cámara et al., 2020	de la Cámara MÁ, Jiménez-Fuente A, Pardos-Sevilla AI. Confinement time due to the COVID-19 disease: An opportunity to promote and engage people in regular physical exer-cise? Transl Sports Med. 2020 May 29:10.1002/tsm2.170. doi: 10.1002/tsm2.170.
Ding et al., 2020	Ding D, Del Pozo Cruz B, Green MA, Bauman AE. Is the COVID-19 lockdown nudging people to be more active: a big data analysis. Br J Sports Med. 2020 Oct;54(20):1183-1184. doi: 10.1136/bjsports-2020-102575.
Gaiha et al., 2021	Gaiha, Shivani & Lempert, Lauren & McKelvey, Karma & Halpern-Felsher, Bonnie. (2021). 109. Adolescent E-Cigarette Use and Access Before and During the COVID-19 Pandemic. Journal of Adolescent Health. 68. S57. 10.1016/j.jadohealth.2020.12.118.
García-Álvarez et al., 2020	García-Álvarez L, Fuente-Tomás L, Sáiz PA, García-Portilla MP, Bobes J. Will changes in alcohol and tobacco use be seen during the COVID-19 lockdown? Adicciones. 2020 Apr 1;32(2):85-89. English, Spanish. doi: 10.20882/adicciones.1546.
Grogan et al., 2020	Grogan S, Walker L, McChesney G, Gee I, Gough B, Cordero MI. How has COVID-19 lockdown impacted smoking? A thematic analysis of written accounts from UK smokers. Psychol Health. 2020 Dec 18:1-17. doi: 10.1080/08870446.2020.1862110.
Gupta & Nebhinani, 2020	Tanu Gupta, Naresh Nebhinani. Impact of COVID-19 pandemic on child and adolescent mental health. Journal of Indian Association for Child and Adolescent Mental Health 2020; 16(3):1-16.

Study	Reference
Heymsfield et al., 2020	Heymsfield, S., Pecoraro, L., Ferruzzi, A., Heo, M., Faith, M., Zoller, T., Antoniazzi, F., Piacentini, G., Nicole Fearnbach, S., Pietrobelli, A. Effects of COVID-19 lockdown on lifestyle behaviors in children with obesity: A longitudinal study. Obesity 2020; 28 (SUPPL 2): 88.
Hnatiuk, 2020	Hnatiuk, Anna. (2020). Social Isolation During COVID-19: Are We Facing the Development of a Magnifier for Cardiovascular Risk Factors. Revista Argentina de Cardiologia. 88. 405-406. 10.7775/rac.v88.i5.19081.
Igic, 2020	Igic, Rajko. (2020). Smoking and COVID-19. Vojnosanitetski pregled. 77. 461-462. 10.2298/VSP2005461I.
Jiménez-Pavón et al., 2020	Jiménez-Pavón D, Carbonell-Baeza A, Lavie CJ. Physical exercise as therapy to fight against the mental and physical consequences of COVID-19 quarantine: Special focus in older people. Prog Cardiovasc Dis. 2020 May-Jun;63(3):386-388. doi: 10.1016/j.pcad.2020.03.009.
Kosravi, 2021	Khosravi M. COVID-19 quarantine: Two-way interaction between physical activity and mental health. Eur J Transl Myol. 2021 Jan 14;30(4):9509. doi: 10.4081/ejtm.2020.9509.
Lakicevic et al., 2020	Lakicevic N, Moro T, Paoli A, Roklicer R, Trivic T, Cassar S, Drid P. Stay fit, don't quit: Geriatric Exercise Prescription in COVID-19 Pandemic. Aging Clin Exp Res. 2020 Jul;32(7):1209-1210. doi: 10.1007/s40520-020-01588-y.
Le Brocq et al., 2020	Le Brocq S, Clare K, Bryant M, Roberts K, Tahrani AA; writing group form Obesity UK; Obesity Empowerment Network; UK Association for the Study of Obesity. Obesity and COVID-19: a call for action from people living with obesity. Lancet Diabetes Endocrinol. 2020 Aug;8(8):652-654. doi: 10.1016/S2213-8587(20)30236-9.
Leong et al., 2021	Leong DP, Eikelboom JW, Yusuf S. The Indirect Consequences of the Response to the COVID-19 Pandemic. J Am Coll Cardiol. 2021 Jan 19;77(2):186-188. doi: 10.1016/j.jacc.2020.11.028.
Lewandowicz et al., 2020	Lewandowicz, M., Kropinska, S., Tobis, S., Kaluzniak-Szymanowska, A., KrzyminskaSiemaszko, R., Wieczorowska-Tobis, K. The impact of COVID-19 on activity and involvement in social life and quality of life among people 60+ living in Poland, depending on the symptoms of depression. European Geriatric Medicine 2020; 11 (SUPPL 1):S17-18.
MacHorn et al., 2020	MacHon, M., Mateo-Abad, M., Vrotsou, K., PenEa, N., Rico, L., Vergara, I. Assessment of health status and lifestyle habits of vulnerable, community-dwelling older adults during the covid-19 confinement. European Geriatric Medicine 2020; 11 (SUPPL 1):S106-7.
Mattioli et al., 2020	Mattioli AV, Nasi M, Cocchi C, Farinetti A. COVID-19 outbreak: impact of the quarantine-induced stress on cardiovascular disease risk burden. Future Cardiol. 2020 Nov;16(6):539-542. doi: 10.2217/fca-2020-0055.
Mehrsafar et al., 2020	Mehrsafar AH, Gazerani P, Moghadam Zadeh A, Jaenes Sánchez JC. Addressing potential impact of COVID-19 pandemic on physical and mental health of elite athletes. Brain Behav Immun. 2020 Jul;87:147-148. doi: 10.1016/j.bbi.2020.05.011.
Middletonn et al., 2020	Middleton, J. T., Konganige, S., Dahaghin, P., Zafar, H., Reddy, A., Martin, C., Thompson, A. A. R., Swift, A. J., Morris, P., Condliffe, R., Morrell, N. W., Kiely, D. G., Toshner, M., Rothman, A. M. Effect of COVID-19 Public Health Measures on Activity and Quality of Life in Patients with Pulmonary Arterial Hypertension. Circulation. Conference: American Heart Association Scientific Sessions, AHA 2020; 142(SUPPL 3)
Morrison et al., 2020	Morrison SA, Jurak G, Starc G. Responding to a global pandemic: Republic of Slovenia on maintaining physical activity during self-isolation. Scand J Med Sci Sports. 2020 Aug;30(8):1546-1548. doi: 10.1111/sms.13745.
Muhammad & Abu-bakar, 2021	Muhammad DG, Abubakar IA. COVID-19 lockdown may increase cardiovascular disease risk factors. Egypt Heart J. 2021 Jan 5;73(1):2. doi: 10.1186/s43044-020-00127-4.
Murukesu et al., 2021	Murukesu, Resshaya & Singh, Devinder Kaur Ajit & Shahar, Suzana & Subramaniam, Ponnusamy. (2021). Physical Activity Patterns, Psychosocial Well-Being and Coping Strategies Among Older Persons with Cognitive Frailty of the "WE-RISE" Trial Throughout the COVID-19 Movement Control Order. Clinical Interventions in Aging. 2021. 415-429. 10.2147/CIA.S290851.
Nava-gonzalez et al., 2020	Nava-gonzalez, E., Silva, I. R., Velezquez, R. P., Hunot, C., Olguin, G. Q., Rasgado, M. C., Del Rosario Silva, M., Delgado De La Cruz, A. C., Zarate, B. E. L. J., Lopez, A., Karina, D., De Ciencias Martinez, I. N., Del Sol Huescas Juarez, M. Perceived changes in diet quality and bmi in mexico before and during the covid-19 quarantine. Obesity 2020, 28 (SUPPL 2): 135.
Nisticò et al., 2021	Nisticò V, Bertelli S, Tedesco R, Anselmetti S, Priori A, Gambini O, Demartini B. The psychological impact of COVID-19-related lockdown measures among a sample of Italian patients with eating disorders: a preliminary longitudinal study. Eat Weight Disord. 2021 Feb 13:1–7. doi: 10.1007/s40519-021-01137-0.
Nyenhuis et al., 20202020	Nyenhuis SM, Greiwe J, Zeiger JS, Nanda A, Cooke A. Exercise and Fitness in the Age of Social Distancing During the COVID-19 Pandemic. J Allergy Clin Immunol Pract. 2020 Jul-Aug;8(7):2152-2155. doi: 10.1016/j.jaip.2020.04.039.
Odone et al.,	Odone A, Lugo A, Amerio A, Borroni E, Bosetti C, Carreras G, Cavalieri d'Oro L, Colombo P, Fanucchi T, Ghislandi S, Gorini G, Iacoviello L, Pacifici R, Santucci C, Serafini G, Signorelli C, Stival C, Stuckler D, Tersalvi CA, Gallus S. COVID-19 lockdown impact on lifestyle habits of Italian adults. Acta Biomed. 2020 Jul 20;91(9-S):87-89. doi: 10.23750/abm.v91i9-S.10122.
Ong et al., 2021	Ong JL, Lau T, Massar SAA, Chong ZT, Ng BKL, Koek D, Zhao W, Yeo BTT, Cheong K, Chee MWL. COVID-19-related mobility reduction: heterogenous effects on sleep and physical activity rhythms. Sleep. 2021 Feb 12;44(2):zsaa179. doi: 10.1093/sleep/zsaa179
Paiva et al., 2020	Paiva, Teresa & Gaspar, Tania & Silva, Maria-Raquel G. & Feliciano, Amélia & Ramiro, Lucia & Gaspar, Susana & Sampaio, Francisco & Videira, Gabriela & Fonseca, Júlio & Pimentel, Joana & Vaz de Castro, Joana & Reis, Cátia & Simião, Hugo & Clara, Ana & de Sousa, Rute & Guedes de Pinho, Lara & Botelho Guedes, Fábio & Branquinho, Cátia & Tomé, Gina & Matos, Margarida. (2020). Gender differences in health risk behaviors and its correlates during COVID-19 pandemic. 10.13140/RG.2.2.16216.32005.

Study	Reference
Park et al., 2020	Park S, Kim B, Lee J. Social Distancing and Outdoor Physical Activity During the COVID-19 Outbreak in South Korea: Implications for Physical Distancing Strategies. Asia Pac J Public Health. 2020 Sep-Oct;32(6-7):360-362. doi: 10.1177/1010539520940929.
Patwardhan, 2020	Patwardhan P. COVID-19: Risk of increase in smoking rates among England's 6 million smokers and relapse among England's 11 million ex-smokers. BJGP Open. 2020 Jun 23;4(2):bjgpopen20X101067. doi: 10.3399/bjgpopen20X101067.
Pecoraro et al., 2020	Pecoraro L, Dalle Carbonare L, De Franceschi L, Piacentini G, Pietrobelli A. The psychophysical impact that COVID-19 has on children must not be underestimated. Acta Paediatr. 2020 Aug;109(8):1679-1680. doi: 10.1111/apa.15347.
Pereira et al., 2020	Pereira, X., Romero-Velez, G., Skendelas, J., Grosser, R., Costinett, S., Creange, C., Moran-Atkin, E., Choi, J., Camacho, D. Effect of City-Wide Lockdown on Early Weight-Loss in Bariatric Patients During the COVID-19 Pandemic. Obesity 2020; 28 (SUPPL 2):84-5.
Petroni et al, 2020	Petroni, M. L., Caretto, A., Pintus, S., Morabito, S., Zuliani, P., Di Carlo, M. S., Sturda, R. A. Factors predicting weight gain during Covid-19 lockdown in patients with obesity: A national survey. Clinical Nutrition ESPEN 2020; 40:659.
Phd, 2021	Phd, Andrew. (2021). 78. The Effects of the COVID-19 Pandemic on Physical Activity and Mental Well-Being in Older Adolescents in College. Journal of Adolescent Health. 68. S42. 10.1016/j.jadohealth.2020.12.087.
Pitanga et al., 2020	Pitanga FJG, Beck CC, Pitanga CPS. Physical Activity And Reducing Sedentary Behavior During The Coronavirus Pandemic. Arq Bras Cardiol. 2020 Jun;114(6):1058-1060. English, Portuguese. doi: 10.36660/abc.20200238.
Rahman et al., 2020	Rahman MS, Lassi ZS, Shariful Islam SM. Risks to Bangladeshi children and young people during covid-19 outbreak. BMJ. 2020 Jun 11;369:m2299. doi: 10.1136/bmj.m2299.
Reza et al., 2020	Reza N, DeFilippis EM, Jessup M. Secondary Impact of the COVID-19 Pandemic on Patients With Heart Failure. Circ Heart Fail. 2020 May;13(5):e007219. doi: 10.1161/CIR-CHEARTFAILURE.120.007219.
Rivan et al., 2020	Rivan, N. F. M., Shahar, S., Singh, D. K. A., Ibrahim, N., Ludin, A. F. M., Yahya, H. M., Sakian, N. I. M., Mahadzir, H., Subramaniam, P., Kamaruddin, M. Z. A., Murukesu, R., Goh, J. W., Malek, M. R. A., Rashid, R. General psychological health status and coping strategies during Lock-down due to COVID-19 outbreak among Malaysian Older Adults. European Geriatric Medicine 2020; 11 (SUPPL 1):S95-6.
Rundle et al., 2020	Rundle AG, Park Y, Herbstman JB, Kinsey EW, Wang YC. COVID-19-Related School Closings and Risk of Weight Gain Among Children. Obesity (Silver Spring). 2020 Jun;28(6):1008-1009. doi: 10.1002/oby.22813.
Schladen et al., 2020	Schladen, Manon & Weidemann, Deb & Pizzino, Kenzi & Cassetty, Tami & Grufstedt, Maria & Martinez, Brandon & Boxtel, Amanda. (2020). Changes in Physical Activity, Health, and Wellness Experienced by Persons with Neurologic Impairments During the COVID-19 Pandemic. Archives of Physical Medicine and Rehabilitation. 101. e132. 10.1016/j.apmr.2020.10.020.
Siddiqui et al., 2020	Siddiqui, Shafayet & Jakaria, Md. (2020). Lockdown leading obesity and its possible impacts on the second wave of COVID-19. Bangladesh Journal of Medical Science. 2020. 10.3329/bjms.v19i0.48172.
The Lancet, 2020	The Lancet Gastroenterology Hepatology. Drinking alone: COVID-19, lockdown, and alcohol-related harm. Lancet Gastroenterol Hepatol. 2020 Jul;5(7):625. doi: 10.1016/S2468-1253(20)30159-X.
Timpka, 2020	Timpka T. Sport in the tracks and fields of the corona virus: Critical issues during the exit from lockdown. J Sci Med Sport. 2020 Jul;23(7):634-635. doi: 10.1016/j.jsams.2020.05.001.
Tom et al., 2020	Tom, M. R., Dearborn-Tomazos, J. L. Impact of covid-19-related physical distancing on mental and physical health of people with recent stroke. Annals of Neurology 2020; 88 (SUPPL 25): S91-2.
Udigwe et al., 2021	Udigwe, Ifeoma & Mbachu, Chioma & Onukwuli, Vivian & Anyanwu, Onyinye & Mbanuzuru, Ahoma & Umeh, Uche. (2021). 92. The Impact of CORVID-19 on Adolescents and Young People Living in Southeast Nigeria. Journal of Adolescent Health. 68. S49. 10.1016/j.jadohealth.2020.12.101.
Undela et al., 2020	P.B, S. & Undela, Krishna & R.K.Puvavada, & Gupta, Balaji & Manohar Rao, Kishor. (2020). To Assess the psychological impact of COVID-19 lockdown among healthy individuals in India. Value in Health. 23. S589. 10.1016/j.jval.2020.08.1107.
Usman & Fahy, 2020	Usman M, Fahy S. Coping with the COVID-19 crisis: an overview of service adaptation and challenges encountered by a rural Psychiatry of Later Life (POLL) team. Ir J Psychol Med. 2020 Jul 2:1-5. doi: 10.1017/ipm.2020.86.
Valenzuela et al., 2020	Valenzuela PL, Santos-Lozano A, Lista S, Serra-Rexach JA, Emanuele E, Lucia A. Coronavirus Lockdown: Forced Inactivity for the Oldest Old? J Am Med Dir Assoc. 2020 Jul;21(7):988-989. doi: 10.1016/j.jamda.2020.03.026.
Wang et al., 2020	Wang, Y., Shi, Z., Yan, A., Zheng, J., Xue, Q., Qu, W., Wu, J. COVID-19 pandemic and obesity-related outcomes: A nationwide survey study of 10,139 adults in China. Obesity 2020; 28(SUPPL 2):73.
Yach, 2020	Yach D. Tobacco Use Patterns in Five Countries During the COVID-19 Lockdown. Nicotine Tob Res. 2020 Aug 24;22(9):1671-1672. doi: 10.1093/ntr/ntaa097.
Yan et al., 2020	Yan, A., Wang, Y., Sun, X., Zhao, B., Baibing Mi, D., Shi, Z. Physical activity, sedentary behaviors, and weight gain during covid-19 in China. Obesity 2020; 28 (SUPPL 2):137.

S2.1.4 Excluded Fulltexts – Reason: Missing reference values

Study	Reference
Abate Daga et al., 2021	Abate Daga F, Agostino S, Peretti S, Beratto L. COVID-19 nationwide lockdown and physical activity profiles among North-western Italian population using the International Physical Activity Questionnaire (IPAQ). Sport Sci Health. 2021 Mar 4:1-6. doi: 10.1007/s11332-021-00745-8.
Alves et al., 2021	Alves JM, Yunker AG, DeFendis A, Xiang AH, Page KA. BMI status and associations between affect, physical activity and anxiety among U.S. children during COVID-19. Pediatr Obes. 2021 Mar 15:e12786. doi: 10.1111/ijpo.12786. Epub ahead of print. PMID: 33720550.
Amatori et al., 2020	Amatori S, Donati Zeppa S, Preti A, Gervasi M, Gobbi E, Ferrini F, Rocchi MBL, Baldari C, Perroni F, Piccoli G, Stocchi V, Sestili P, Sisti D. Dietary Habits and Psychological States during COVID-19 Home Isolation in Italian College Students: The Role of Physical Exercise. Nutrients. 2020 Nov 28;12(12):3660. doi: 10.3390/nu12123660.
Balanzá-Martínez et al., 2021	Balanzá-Martínez V, Kapczinski F, de Azevedo Cardoso T, Atienza-Carbonell B, Rosa AR, Mota JC, De Boni RB. The assessment of lifestyle changes during the COVID-19 pandemic using a multidimensional scale. Rev Psiquiatr Salud Ment. 2021 Jan-Mar;14(1):16-26. doi: 10.1016/j.rpsm.2020.07.003.
Barrea et al., 2020	Barrea L, Pugliese G, Framondi L, Di Matteo R, Laudisio D, Savastano S, Colao A, Muscogiuri G. Does Sars-Cov-2 threaten our dreams? Effect of quarantine on sleep quality and body mass index. J Transl Med. 2020 Aug 18;18(1):318. doi: 10.1186/s12967-020-02465-y.
Barwais, 2020	Barwais, Faisal. (2020). Assessing physical activity and sedentary time during the COVID-19 pandemic using self-reported measurement. National Journal of Physiology, Pharmacy and Pharmacology. 10. 1. 10.5455/njppp.2020.10.09241202001102020.
Boukrim et al., 2021	Boukrim M, Obtel M, Kasouati J, Achbani A, Razine R. Covid-19 and Confinement: Effect on Weight Load, Physical Activity and Eating Behavior of Higher Education Students in Southern Morocco. Ann Glob Health. 2021 Jan 6;87(1):7. doi: 10.5334/aogh.3144.
Budimir et al., 2021	Budimir S, Probst T, Pieh C. Coping strategies and mental health during COVID-19 lockdown. J Ment Health. 2021 Jan 27:1-8. doi: 10.1080/09638237.2021.1875412.
Cachón-Zagalaz et al., 2021	Cachón-Zagalaz J, Zagalaz-Sánchez MªL, Arufe-Giráldez V, Sanmiguel-Rodríguez A, González-Valero G. Physical Activity and Daily Routine among Children Aged 0-12 during the COVID-19 Pandemic in Spain. Int J Environ Res Public Health. 2021 Jan 15;18(2):703. doi: 10.3390/ijerph18020703.
Carriedo et al., 2020	Carriedo A, Cecchini JA, Fernandez-Rio J, Méndez-Giménez A. COVID-19, Psychological Well-being and Physical Activity Levels in Older Adults During the Nationwide Lockdown in Spain. Am J Geriatr Psychiatry. 2020 Nov;28(11):1146-1155. doi: 10.1016/j.jagp.2020.08.007.
Cecchetto et al., 2021	Cecchetto C, Aiello M, Gentili C, Ionta S, Osimo SA. Increased emotional eating during COVID-19 associated with lockdown, psychological and social distress. Appetite. 2021 May 1;160:105122. doi: 10.1016/j.appet.2021.105122.
Di Dalmazi et al., 2020	Di Dalmazi G, Maltoni G, Bongiorno C, Tucci L, Di Natale V, Moscatiello S, Laffi G, Pession A, Zucchini S, Pagotto U. Comparison of the effects of lockdown due to COVID-19 on glucose patterns among children, adolescents, and adults with type 1 diabetes: CGM study. BMJ Open Diabetes Res Care. 2020 Oct;8(2):e001664. doi: 10.1136/bmjdrc-2020-001664.
Diniz et al., 2020	Diniz TA, Christofaro DGD, Tebar WR, Cucato GG, Botero JP, Correia MA, Ritti-Dias RM, Lofrano-Prado MC, Prado WL. Reduction of Physical Activity Levels During the COVID-19 Pandemic Might Negatively Disturb Sleep Pattern. Front Psychol. 2020 Dec 10;11:586157. doi: 10.3389/fpsyg.2020.586157.
Dobler et al., 2020	Dobler CL, Krüger B, Strahler J, Weyh C, Gebhardt K, Tello K, Ghofrani HA, Sommer N, Gall H, Richter MJ, Krüger K. Physical Activity and Mental Health of Patients with Pulmonary Hypertension during the COVID-19 Pandemic. J Clin Med. 2020 Dec 12;9(12):4023. doi: 10.3390/jcm9124023.
Dönmez et al., 2021	Dönmez G, Özkan Ö, Menderes Y, Torgutalp ŞŞ, Karaçoban L, Denerel N, Kudaş S. The effects of home confinement on physical activity level and mental status in professional football players during COVID-19 outbreak. Phys Sportsmed. 2021 Feb 21:1-7. doi: 10.1080/00913847.2021.1888630.
Dragun et al., 2020	Dragun R, Veček NN, Marendić M, Pribisalić A, Đivić G, Cena H, Polašek O, Kolčić I. Have Lifestyle Habits and Psychological Well-Being Changed among Adolescents and Medical Students Due to COVID-19 Lockdown in Croatia? Nutrients. 2020 Dec 30;13(1):97. doi: 10.3390/nu13010097.
Flaudias et al., 2020	Flaudias V, Iceta S, Zerhouni O, Rodgers RF, Billieux J, Llorca PM, Boudesseul J, de Chazeron I, Romo L, Maurage P, Samalin L, Bègue L, Naassila M, Brousse G, Guillaume S. COVID-19 pandemic lockdown and problematic eating behaviors in a student population. J Behav Addict. 2020 Sep 24;9(3):826-835. doi: 10.1556/2006.2020.00053.
Flaudias et al., 2021	Flaudias V, Zerhouni O, Pereira B, Cherpitel CJ, Boudesseul J, de Chazeron I, Romo L, Guillaume S, Samalin L, Cabe J, Bègue L, Gerbaud L, Rolland B, Llorca PM, Naassila M, Brousse G. The Early Impact of the COVID-19 Lockdown on Stress and Addictive Behaviors in an Alcohol-Consuming Student Population in France. Front Psychiatry. 2021 Feb 9;12:628631. doi: 10.3389/fpsyt.2021.628631.
Ginous et al., 2021	Ginoux C, Isoard-Gautheur S, Teran-Escobar C, Forestier C, Chalabaev A, Clavel A, Sarrazin P. Being Active during the Lockdown: The Recovery Potential of Physical Activity for Well-Being. Int J Environ Res Public Health. 2021 Feb 10;18(4):1707. doi: 10.3390/ijerph18041707.
Haddad et al., 2020	Haddad C, Zakhour M, Bou Kheir M, Haddad R, Al Hachach M, Sacre H, Salameh P. Association between eating behavior and quarantine/confinement stressors during the corona-virus disease 2019 outbreak. J Eat Disord. 2020 Sep 1;8:40. doi: 10.1186/s40337-020-00317-0.
Horigian et al., 2021	Horigian VE, Schmidt RD, Feaster DJ. Loneliness, Mental Health, and Substance Use among US Young Adults during COVID-19. J Psychoactive Drugs. 2021 Jan-Mar;53(1):1-9. doi: 10.1080/02791072.2020.1836435.

Study	Reference
Khare & Jindal, 2020	Khare J, Jindal S. Observational study on Effect of Lock Down due to COVID 19 on glycemic control in patients with Diabetes: Experience from Central India. Diabetes Metab Syndr. 2020 Nov-Dec;14(6):1571-1574. doi: 10.1016/j.dsx.2020.08.012.
Kilani et al., 2020	Kilani HA, Bataineh MF, Al-Nawayseh A, Atiyat K, Obeid O, Abu-Hilal MM, Mansi T, Al-Kilani M, Al-Kitani M, El-Saleh M, Jaber RM, Sweidan A, Himsi M, Yousef I, Alzeer F, Nasral-lah M, Al Dhaheri AS, Al-Za'abi A, Allala O, Al-Kilani L, Alhasan AM, Ghieda M, Najah Y, Alsheekhly S, Alhaifi A, Shukri R, Al Adwani J, Waly M, Kilani L, Kilani LH, Al Shareef AS, Kilani A. Healthy lifestyle behaviors are major predictors of mental wellbeing during COVID-19 pandemic confinement: A study on adult Arabs in higher educational institutions. PLoS One. 2020 Dec 14;15(12):e0243524. doi: 10.1371/journal.pone.0243524.
Jacob et al., 2020	Jacob L, Tully MA, Barnett Y, Lopez-Sanchez GF, Butler L, Schuch F, López-Bueno R, McDermott D, Firth J, Grabovac I, Yakkundi A, Armstrong N, Young T, Smith L. The relation-ship between physical activity and mental health in a sample of the UK public: A cross-sectional study during the implementation of COVID-19 social distancing measures. Ment Health Phys Act. 2020 Oct;19:100345. doi: 10.1016/j.mhpa.2020.100345.
Maertl et al., 2021	Maertl T, De Bock F, Huebl L, Oberhauser C, Coenen M, Jung-Sievers C, On Behalf Of The Cosmo Study Team. Physical Activity during COVID-19 in German Adults: Analyses in the COVID-19 Snapshot Monitoring Study (COSMO). Int J Environ Res Public Health. 2021 Jan 9;18(2):507. doi: 10.3390/ijerph18020507.
McDowell et al., 2020	McDowell CP, Herring MP, Lansing J, Brower C, Meyer JD. Working From Home and Job Loss Due to the COVID-19 Pandemic Are Associated With Greater Time in Sedentary Behaviors. Front Public Health. 2020 Nov 5;8:597619. doi: 10.3389/fpubh.2020.597619.
Méndez-Giménez et al., 2021	Méndez-Giménez A, Cecchini JA, Fernández-Río J, Carriedo A. Physical Activity and Prevention of Depressive Symptoms in the Spanish Population during Confinement due to COVID-19. Psicothema. 2021 Feb;33(1):111-117. doi: 10.7334/psicothema2020.294.
Meyer et al., 2002	Meyer J, Herring M, McDowell C, Lansing J, Brower C, Schuch F, Smith L, Tully M, Martin J, Caswell S, Cortes N, Boolani A. Joint prevalence of physical activity and sitting time during COVID-19 among US adults in April 2020. Prev Med Rep. 2020 Dec;20:101256. doi: 10.1016/j.pmedr.2020.101256.
Nicodemo et al., 2021	Nicodemo M, Spreghini MR, Manco M, Wietrzykowska Sforza R, Morino G. Childhood Obesity and COVID-19 Lockdown: Remarks on Eating Habits of Patients Enrolled in a Food-Education Program. Nutrients. 2021 Jan 26;13(2):383. doi: 10.3390/nu13020383.
Ozdemir et al., 2020	Ozdemir F, Cansel N, Kizilay F, Guldogan E, Ucuz I, Sinanoglu B, Colak C, Cumurcu HB. The role of physical activity on mental health and quality of life during COVID-19 outbreak: A cross-sectional study. Eur J Integr Med. 2020 Dec;40:101248. doi: 10.1016/j.eujim.2020.101248.
Panno et al., 2020	Panno A, Carbone GA, Massullo C, Farina B, Imperatori C. COVID-19 Related Distress Is Associated With Alcohol Problems, Social Media and Food Addiction Symptoms: Insights From the Italian Experience During the Lockdown. Front Psychiatry. 2020 Nov 25;11:577135. doi: 10.3389/fpsyt.2020.577135.
Patel et al., 2020	Shweta Patel, Manindra Rajak, Kamini Khatak, Ranjana Patnaik, Diwakar Chauhan. ASSESSMENT of IMPACT of COVID-19 (NOVEL CORONA VIRUS DISEASE) on ADULT INDIAN POPULATION: A CROSS SECTIONAL QUESTIONNAIRE BASED SURVEY. European Journal of Molecular & Clinical Medicine, 2020, 7(9):10-23
Pombo et al., 2020	Pombo A, Luz C, Rodrigues LP, Ferreira C, Cordovil R. Correlates of children's physical activity during the COVID-19 confinement in Portugal. Public Health. 2020 Dec;189:14-19. doi: 10.1016/j.puhe.2020.09.009.
Rahman et al., 2020	Rahman ME, Islam MS, Bishwas MS, Moonajilin MS, Gozal D. Physical inactivity and sedentary behaviors in the Bangladeshi population during the COVID-19 pandemic: An online cross-sectional survey. Heliyon. 2020 Oct 30;6(10):e05392. doi: 10.1016/j.heliyon.2020.e05392.
Roe et al., 2021	Roe A, Blikstad-Balas M, Dalland CP. The Impact of COVID-19 and Homeschooling on Students' Engagement With Physical Activity. Front Sports Act Living. 2021 Jan 26;2:589227. doi: 10.3389/fspor.2020.589227.
Sang et al., 2021	Sang X, Menhas R, Saqib ZA, Mahmood S, Weng Y, Khurshid S, Iqbal W, Shahzad B. The Psychological Impacts of COVID-19 Home Confinement and Physical Activity: A Struc-tural Equation Model Analysis. Front Psychol. 2021 Jan 15;11:614770. doi: 10.3389/fpsyg.2020.614770.
Schuch et al., 2020	Schuch FB, Bulzing RA, Meyer J, Vancampfort D, Firth J, Stubbs B, Grabovac I, Willeit P, Tavares VDO, Calegaro VC, Deenik J, López-Sánchez GF, Veronese N, Caperchione CM, Sadarangani KP, Abufaraj M, Tully MA, Smith L. Associations of moderate to vigorous physical activity and sedentary behavior with depressive and anxiety symptoms in self-isolat-ing people during the COVID-19 pandemic: A cross-sectional survey in Brazil. Psychiatry Res. 2020 Oct;292:113339. doi: 10.1016/j.psychres.2020.113339.
Slimani et al., 2020	Slimani M, Paravlic A, Mbarek F, Bragazzi NL, Tod D. The Relationship Between Physical Activity and Quality of Life During the Confinement Induced by COVID-19 Outbreak: A Pilot Study in Tunisia. Front Psychol. 2020 Aug 7;11:1882. doi: 10.3389/fpsyg.2020.01882.
Smith et al., 2020	Smith L, Jacob L, Trott M, Yakkundi A, Butler L, Barnett Y, Armstrong NC, McDermott D, Schuch F, Meyer J, López-Bueno R, Sánchez GFL, Bradley D, Tully MA. The association between screen time and mental health during COVID-19: A cross sectional study. Psychiatry Res. 2020 Oct;292:113333. doi: 10.1016/j.psychres.2020.113333.
Taeymans et al., 2021	Taeymans J, Luijckx E, Rogan S, Haas K, Baur H. Physical Activity, Nutritional Habits, and Sleeping Behavior in Students and Employees of a Swiss University During the COVID-19 Lockdown Period: Questionnaire Survey Study. JMIR Public Health Surveill. 2021 Apr 13;7(4):e26330. doi: 10.2196/26330.
Tao et al., 2020	Tao J, Gao L, Liu Q, Dong K, Huang J, Peng X, Yang Y, Wang H, Yu X. Factors contributing to glycemic control in diabetes mellitus patients complying with home quarantine during the coronavirus disease 2019 (COVID-19) epidemic. Diabetes Res Clin Pract. 2020 Dec;170:108514. doi: 10.1016/j.diabres.2020.108514.
Totosy et al. 2021	Totosy de Zepetnek JO, Martin J, Cortes N, Caswell S, Boolani A. Influence of grit on lifestyle factors during the COVID-19 pandemic in a sample of adults in the United States. Pers Individ Dif. 2021 Jun;175:110705. doi: 10.1016/j.paid.2021.110705.

Study	Reference
Valadez et al., 2020	Valadez MLD, Rodríguez-Naveiras E, Castellanos-Simons D, López-Aymes G, Aguirre T, Flores JF, Borges Á. Physical Activity and Well-Being of High Ability Students and Community Samples During the COVID-19 Health Alert. Front Psychol. 2020 Dec 21;11:606167. doi: 10.3389/fpsyg.2020.606167.
Werneck et al., 2020	Werneck AO, Silva DR, Malta DC, Lima MG, Souza-Júnior PRB, Azevedo LO, Barros MBA, Szwarcwald CL. The mediation role of sleep quality in the association between the incidence of unhealthy movement behaviors during the COVID-19 quarantine and mental health. Sleep Med. 2020 Dec;76:10-15. doi: 10.1016/j.sleep.2020.09.021.
Zhang et al., 2020	Zhang X, Zhu W, Kang S, Qiu L, Lu Z, Sun Y. Association between Physical Activity and Mood States of Children and Adolescents in Social Isolation during the COVID-19 Epidemic. Int J Environ Res Public Health. 2020 Oct 21;17(20):7666. doi: 10.3390/ijerph17207666.
Zhao et al., 2020	Zhao Y, Guo Y, Xiao Y, Zhu R, Sun W, Huang W, Liang D, Tang L, Zhang F, Zhu D, Wu JL. The Effects of Online Homeschooling on Children, Parents, and Teachers of Grades 1-9 During the COVID-19 Pandemic. Med Sci Monit. 2020 Sep 12;26:e925591. doi: 10.12659/MSM.925591.

S2.1.5 EXCLUDED FULLTEXTS – REASON: UNCLEAR RECRUITMENT PROCEDURE

Study	Reference
Al Agha et al., 2021	Al Agha AE, Alharbi RS, Almohammadi OA, Yousef SY, Sulimani AE, Alaama RA. Impact of COVID-19 lockdown on glycemic control in children and adolescents. Saudi Med J. 2021 Jan;42(1):44-48. doi: 10.15537/smj.2021.1.25620.
Al Fagih et al., 2020	Al Fagih A, Al Onazi M, Al Basiri S, Al-Kaf F, Dagriri K, Al Hebaishi Y, Samargandy S, Al Shengeiti L. Remotely monitored inactivity due to COVID-19 lockdowns. Potential hazard for heart failure patients. Saudi Med J. 2020 Nov;41(11):1211-1216. doi: 10.15537/smj.2020.11.25449.
Almadoz et al., 2021	Almandoz JP, Xie L, Schellinger JN, Mathew MS, Bismar N, Ofori A, Kukreja S, Schneider B, Vidot D, Messiah SE. Substance use, mental health and weight-related behaviours during the COVID-19 pandemic in people with obesity. Clin Obes. 2021 Apr;11(2):e12440. doi: 10.1111/cob.12440.
Almadoz et al., 2020	Almandoz JP, Xie L, Schellinger JN, Mathew MS, Gazda C, Ofori A, Kukreja S, Messiah SE. Impact of COVID-19 stay-at-home orders on weight-related behaviours among patients with obesity. Clin Obes. 2020 Oct;10(5):e12386. doi: 10.1111/cob.12386.
Alonso-Martinez et al., 2021	Alonso-Martínez AM, Ramírez-Vélez R, García-Alonso Y, Izquierdo M, García-Hermoso A. Physical Activity, Sedentary Behavior, Sleep and Self-Regulation in Spanish Preschoolers during the COVID-19 Lockdown. Int J Environ Res Public Health. 2021 Jan 15;18(2):693. doi: 10.3390/ijerph18020693.
Alshareef et al., 2020	Alshareef R, Al Zahrani A, Alzahrani A, Ghandoura L. Impact of the COVID-19 lockdown on diabetes patients in Jeddah, Saudi Arabia. Diabetes Metab Syndr. 2020 Sep-Oct;14(5):1583-1587. doi: 10.1016/j.dsx.2020.07.051.
Arai et al., 2021	Arai Y, Oguma Y, Abe Y, Takayama M, Hara A, Urushihara H, Takebayashi T. Behavioral changes and hygiene practices of older adults in Japan during the first wave of COVID-19 emergency. BMC Geriatr. 2021 Feb 24;21(1):137. doi: 10.1186/s12877-021-02085-1.
Azizi et al., 2020	Azizi A, Achak D, Aboudi K, Saad E, Nejjari C, Nouira Y, Hilali A, Youlyouz-Marfak I, Marfak A. Health-related quality of life and behavior-related lifestyle changes due to the COVID-19 home confinement: Dataset from a Moroccan sample. Data Brief. 2020 Oct;32:106239. doi: 10.1016/j.dib.2020.106239.
Bailey et al., 2021	Bailey L, Ward M, DiCosimo A, Baunta S, Cunningham C, Romero-Ortuno R, Kenny RA, Purcell R, Lannon R, McCarroll K, Nee R, Robinson D, Lavan A, Briggs R. Physical and Mental Health of Older People while Cocooning during the COVID-19 Pandemic. QJM. 2021 Jan 20:hcab015. doi: 10.1093/qjmed/hcab015.
Balci et al., 2021	Balci B, Aktar B, Buran S, Tas M, Donmez Colakoglu B. Impact of the COVID-19 pandemic on physical activity, anxiety, and depression in patients with Parkinson's disease. Int J Rehabil Res. 2021 Feb 25. doi: 10.1097/MRR.0000000000000460.
Bonora et al., 2020	Bonora BM, Boscari F, Avogaro A, Bruttomesso D, Fadini GP. Glycaemic Control Among People with Type 1 Diabetes During Lockdown for the SARS-CoV-2 Outbreak in Italy. Diabetes Ther. 2020 May 11;11(6):1-11. doi: 10.1007/s13300-020-00829-7.
Brancaccio et al., 2021	Brancaccio M, Mennitti C, Gentile A, Correale L, Buzzachera CF, Ferraris C, Montomoli C, Frisso G, Borrelli P, Scudiero O. Effects of the COVID-19 Pandemic on Job Activity, Dietary Behaviours and Physical Activity Habits of University Population of Naples, Federico II-Italy. Int J Environ Res Public Health. 2021 Feb 5;18(4):1502. doi: 10.3390/ijerph18041502.
Coughenour et al., 2020	Coughenour C, Gakh M, Pharr JR, Bungum T, Jalene S. Changes in Depression and Physical Activity Among College Students on a Diverse Campus After a COVID-19 Stay-at-Home Order. J Community Health. 2020 Nov 9:1–9. doi: 10.1007/s10900-020-00918-5.
Elmacıoğlu et al., 2021	Elmacıoğlu F, Emiroğlu E, Ülker MT, Özyılmaz Kırcali B, Oruç S. Evaluation of nutritional behaviour related to COVID-19. Public Health Nutr. 2021 Feb;24(3):512-518. doi: 10.1017/S1368980020004140.
Franco et al., 2021	Franco E, Urosa J, Barakat R, Refoyo I. Physical Activity and Adherence to the Mediterranean Diet among Spanish Employees in a Health-Promotion Program before and during the COVID-19 Pandemic: The Sanitas-Healthy Cities Challenge. Int J Environ Res Public Health. 2021 Mar 8;18(5):2735. doi: 10.3390/ijerph18052735.

Study	Reference
Ghosal eta l., 2020	Ghosal S, Arora B, Dutta K, Ghosh A, Sinha B, Misra A. Increase in the risk of type 2 diabetes during lockdown for the COVID19 pandemic in India: A cohort analysis. Diabetes Metab Syndr. 2020 Sep-Oct;14(5):949-952. doi: 10.1016/j.dsx.2020.06.020.
Ghosh et al., 2020	Ghosh A, Arora B, Gupta R, Anoop S, Misra A. Effects of nationwide lockdown during COVID-19 epidemic on lifestyle and other medical issues of patients with type 2 diabetes in north India. Diabetes Metab Syndr. 2020 Sep-Oct;14(5):917-920. doi: 10.1016/j.dsx.2020.05.044.
Glowacz & Schmits, 2020	Glowacz F, Schmits E. Psychological distress during the COVID-19 lockdown: The young adults most at risk. Psychiatry Res. 2020 Nov;293:113486. doi: 10.1016/j.psy-chres.2020.113486.
Kim et al., 2020	Kim JU, Majid A, Judge R, Crook P, Nathwani R, Selvapatt N, Lovendoski J, Manousou P, Thursz M, Dhar A, Lewis H, Vergis N, Lemoine M. Effect of COVID-19 lockdown on alco-hol consumption in patients with pre-existing alcohol use disorder. Lancet Gastroenterol Hepatol. 2020 Oct;5(10):886-887. doi: 10.1016/S2468-1253(20)30251-X.
Majumdar et al., 2020	Majumdar P, Biswas A, Sahu S. COVID-19 pandemic and lockdown: cause of sleep disruption, depression, somatic pain, and increased screen exposure of office workers and students of India. Chronobiol Int. 2020 Aug;37(8):1191-1200. doi: 10.1080/07420528.2020.1786107.
Malanchini et al., 2020	Malanchini G, Malacrida M, Ferrari P, Leidi C, Ferrari G, Racheli M, Senni M, Filippo P. Impact of the Coronavirus Disease-19 Outbreak on Physical Activity of Patients With Implant-able Cardioverter Defibrillators. J Card Fail. 2020 Oct;26(10):898-899. doi: 10.1016/j.cardfail.2020.08.005.
Marco-Ahullo et al., 2021	Marco-Ahulló A, Montesinos-Magraner L, González LM, Morales J, Bernabéu-García JA, García-Massó X. Impact of COVID-19 on the self-reported physical activity of people with complete thoracic spinal cord injury full-time manual wheelchair users. J Spinal Cord Med. 2021 Jan 19:1-5. doi: 10.1080/10790268.2020.1857490.
Marty et al., 2021	Marty L, de Lauzon-Guillain B, Labesse M, Nicklaus S. Food choice motives and the nutritional quality of diet during the COVID-19 lockdown in France. Appetite. 2021 Feb 1;157:105005. doi: 10.1016/j.appet.2020.105005.
McCarthy et al., 2021	McCarthy H, Potts HWW, Fisher A. Physical Activity Behavior Before, During, and After COVID-19 Restrictions: Longitudinal Smartphone-Tracking Study of Adults in the United Kingdom. J Med Internet Res. 2021 Feb 3;23(2):e23701. doi: 10.2196/23701.
Önmez et al., 2020	Önmez A, Gamsızkan Z, Özdemir Ş, Kesikbaş E, Gökosmanoğlu F, Torun S, Cinemre H. The effect of COVID-19 lockdown on glycemic control in patients with type 2 diabetes mellitus in Turkey. Diabetes Metab Syndr. 2020 Nov-Dec;14(6):1963-1966. doi: 10.1016/j.dsx.2020.10.007.
Pietrobelli et al. ,2020	Pietrobelli A, Pecoraro L, Ferruzzi A, Heo M, Faith M, Zoller T, Antoniazzi F, Piacentini G, Fearnbach SN, Heymsfield SB. Effects of COVID-19 Lockdown on Lifestyle Behaviors in Children with Obesity Living in Verona, Italy: A Longitudinal Study. Obesity (Silver Spring). 2020 Aug;28(8):1382-1385. doi: 10.1002/oby.22861.
Rastogi et al., 2020	Rastogi A, Hiteshi P, Bhansali A. Improved glycemic control amongst people with long-standing diabetes during COVID-19 lockdown: a prospective, observational, nested cohort study. Int J Diabetes Dev Ctries. 2020 Oct 21:1-6. doi: 10.1007/s13410-020-00880-x.
Romero-Blanco et al., 2020	Romero-Blanco C, Rodríguez-Almagro J, Onieva-Zafra MD, Parra-Fernández ML, Prado-Laguna MDC, Hernández-Martínez A. Physical Activity and Sedentary Lifestyle in Univer-sity Students: Changes during Confinement Due to the COVID-19 Pandemic. Int J Environ Res Public Health. 2020 Sep 9;17(18):6567. doi: 10.3390/ijerph17186567.
Sama et al., 2021	Sama BK, Kaur P, Thind PS, Verma MK, Kaur M, Singh DD. Implications of COVID-19-induced nationwide lockdown on children's behaviour in Punjab, India. Child Care Health Dev. 2021 Jan;47(1):128-135. doi: 10.1111/cch.12816.
Sankar et al., 2020	Sankar P, Ahmed WN, Mariam Koshy V, Jacob R, Sasidharan S. Effects of COVID-19 lockdown on type 2 diabetes, lifestyle and psychosocial health: A hospital-based cross-sec-tional survey from South India. Diabetes Metab Syndr. 2020 Nov-Dec;14(6):1815-1819. doi: 10.1016/j.dsx.2020.09.005.
Sassone et al., 2020	Sassone B, Mandini S, Grazzi G, Mazzoni G, Myers J, Pasanisi G. Impact of COVID-19 Pandemic on Physical Activity in Patients With Implantable Cardioverter-Defibrillators. J Cardiopulm Rehabil Prev. 2020 Sep;40(5):285-286. doi: 10.1097/HCR.0000000000000539.
Sisto et al., 2020	Sisto A, Vicinanza F, Tuccinardi D, Watanabe M, Gallo IF, D'Alessio R, Manfrini S, Quintiliani L. The psychological impact of COVID-19 pandemic on patients included in a bariatric surgery program. Eat Weight Disord. 2020 Aug 28:1–11. doi: 10.1007/s40519-020-00988-3.
Srivastav et al., 2021	Srivastav AK, Sharma N, Samuel AJ. Impact of Coronavirus disease-19 (COVID-19) lockdown on physical activity and energy expenditure among physiotherapy professionals and students using web-based open E-survey sent through WhatsApp, Facebook and Instagram messengers. Clin Epidemiol Glob Health. 2021 Jan-Mar;9:78-84. doi: 10.1016/j.cegh.2020.07.003.
Utama et al., 2020	Utama, Lalu & Yunianto, Andi & Shagti, Indhira & Gressilda, Juni & Sine, Louisa & Agung, Anak & Mirah, Ayu & Loaloka, Meirina & Nur, Astuti. (2020). European Journal of Molecu-lar & Clinical Medicine Impact of the COVID-19 Epidemic on Eating Habits and Lifestyle : An East Nusa Tenggara Survey.
Zhu et al., 2021	Zhu Q, Li M, Ji Y, Shi Y, Zhou J, Li Q, Qin R, Zhuang X. "Stay-at-Home" Lifestyle Effect on Weight Gain during the COVID-19 Outbreak Confinement in China. Int J Environ Res Public Health. 2021 Feb 12;18(4):1813. doi: 10.3390/ijerph18041813. PMID: 33673375; PMCID: PMC7918476.

S2.1.6 Excluded Fulltexts – Reason: Outcome

Study	Reference
Alfawaz et al., 2021	Alfawaz H, Yakout SM, Wani K, Aljumah GA, Ansari MGA, Khattak MNK, Hussain SD, Al-Daghri NM. Dietary Intake and Mental Health among Saudi Adults during COVID-19 Lock-down. Int J Environ Res Public Health. 2021 Feb 9;18(4):1653. doi: 10.3390/ijerph18041653.
Ali et al., 2021	Ali A, Siddiqui AA, Arshad MS, Iqbal F, Arif TB. Effects of COVID-19 pandemic and lockdown on lifestyle and mental health of students: A retrospective study from Karachi,Pakistan. Ann Med Psychol (Paris). 2021 Feb 15. doi: 10.1016/j.amp.2021.02.004.
Al-Musharaf, 2020	Al-Musharaf S. Prevalence and Predictors of Emotional Eating among Healthy Young Saudi Women during the COVID-19 Pandemic. Nutrients. 2020 Sep 24;12(10):2923. doi: 10.3390/nu12102923. PMID: 32987773; PMCID: PMC7598723.
Ares et al., 2021	Ares G, Bove I, Vidal L, Brunet G, Fuletti D, Arroyo Á, Blanc MV. The experience of social distancing for families with children and adolescents during the coronavirus (COVID-19) pandemic in Uruguay: Difficulties and opportunities. Child Youth Serv Rev. 2021 Feb;121:105906. doi: 10.1016/j.childyouth.2020.105906.
Bade et al., 2020	Bade R, Simpson BS, Ghetia M, Nguyen L, White JM, Gerber C. Changes in alcohol consumption associated with social distancing and self-isolation policies triggered by COVID-19 in South Australia: a wastewater analysis study. Addiction. 2020 Sep 18:10.1111/add.15256. doi: 10.1111/add.15256.
Balhara et al., 2020	Balhara YPS, Singh S, Narang P. Effect of lockdown following COVID-19 pandemic on alcohol use and help-seeking behavior: Observations and insights from a sample of alcohol use disorder patients under treatment from a tertiary care center. Psychiatry Clin Neurosci. 2020 Aug;74(8):440-441. doi: 10.1111/pcn.13075.
Barguilla et al., 2020	Barguilla A, Fernández-Lebrero A, Estragués-Gázquez I, García-Escobar G, Navalpotro-Gómez I, Manero RM, Puente-Periz V, Roquer J, Puig-Pijoan A. Effects of COVID-19 Pan-demic Confinement in Patients With Cognitive Impairment. Front Neurol. 2020 Nov 24;11:589901. doi: 10.3389/fneur.2020.589901.
Ben-Ezra et al., 2020	Ben-Ezra M, Sun S, Hou WK, Goodwin R. The association of being in quarantine and related COVID-19 recommended and non-recommended behaviors with psychological distress in Chinese population. J Affect Disord. 2020 Oct 1;275:66-68. doi: 10.1016/j.jad.2020.06.026.
Chirico et al., 2020	Chirico A, Lucidi F, Galli F, Giancamilli F, Vitale J, Borghi S, La Torre A, Codella R. COVID-19 Outbreak and Physical Activity in the Italian Population: A Cross-Sectional Analysis of the Underlying Psychosocial Mechanisms. Front Psychol. 2020 Aug 21;11:2100. doi: 10.3389/fpsyg.2020.02100.
Di Renzo et al., 2020	Di Renzo L, Gualtieri P, Cinelli G, Bigioni G, Soldati L, Attinà A, Bianco FF, Caparello G, Camodeca V, Carrano E, Ferraro S, Giannattasio S, Leggeri C, Rampello T, Lo Presti L, Tarsitano MG, De Lorenzo A. Psychological Aspects and Eating Habits during COVID-19 Home Confinement: Results of EHLC-COVID-19 Italian Online Survey. Nutrients. 2020 Jul 19;12(7):2152. doi: 10.3390/nu12072152.
Ezpeleta et al., 2020	Ezpeleta L, Navarro JB, de la Osa N, Trepat E, Penelo E. Life Conditions during COVID-19 Lockdown and Mental Health in Spanish Adolescents. Int J Environ Res Public Health. 2020 Oct 7;17(19):7327. doi: 10.3390/ijerph17197327.
Ford, 2021	Ford MB. Social distancing during the COVID-19 pandemic as a predictor of daily psychological, social, and health-related outcomes. J Gen Psychol. 2021 Jul;148(3):249-271. doi: 10.1080/00221309.2020.1860890.
Głąbska et al., 2020	Głąbska D, Skolmowska D, Guzek D. Population-Based Study of the Changes in the Food Choice Determinants of Secondary School Students: Polish Adolescents' COVID-19 Experience (PLACE-19) Study. Nutrients. 2020 Aug 30;12(9):2640. doi: 10.3390/nu12092640.
Gupta et al., 2020	Gupta R, Grover S, Basu A, Krishnan V, Tripathi A, Subramanyam A, Nischal A, Hussain A, Mehra A, Ambekar A, Saha G, Mishra KK, Bathla M, Jagiwala M, Manjunatha N, Nebhinani N, Gaur N, Kumar N, Dalal PK, Kumar P, Midha PK, Daga R, Tikka SK, Praharaj SK, Goyal SK, Kanchan S, Sarkar S, Das S, Sarkhel S, Padhy SK, Sahoo S, Satyanarayana Rao TS, Dubey V, Menon V, Chhabra V, Lahan V, Avasthi A. Changes in sleep pattern and sleep quality during COVID-19 lockdown. Indian J Psychiatry. 2020 Jul-Aug;62(4):370-378. doi: 10.4103/psychiatry.IndianJPsychiatry_523_20.
Kalkan et al., 2021	Kalkan Uğurlu Y, Mataracı Değirmenci D, Durgun H, Gök Uğur H. The examination of the relationship between nursing students' depression, anxiety and stress levels and restrictive, emotional, and external eating behaviors in COVID-19 social isolation process. Perspect Psychiatr Care. 2021 Apr;57(2):507-516. doi: 10.1111/ppc.12703.
Keel et al., 2020	Keel PK, Gomez MM, Harris L, Kennedy GA, Ribeiro J, Joiner TE. Gaining "The Quarantine 15:" Perceived versus observed weight changes in college students in the wake of COVID-19. Int J Eat Disord. 2020 Nov;53(11):1801-1808. doi: 10.1002/eat.23375.
Khan et al., 2020	Khan AH, Sultana MS, Hossain S, Hasan MT, Ahmed HU, Sikder MT. The impact of COVID-19 pandemic on mental health & wellbeing among home-quarantined Bangladeshi students: A cross-sectional pilot study. J Affect Disord. 2020 Dec 1;277:121-128. doi: 10.1016/j.jad.2020.07.135.
Li et al., 2021	Li W, Zhang Y, Wang J, Ozaki A, Wang Q, Chen Y, Jiang Q. Association of Home Quarantine and Mental Health Among Teenagers in Wuhan, China, During the COVID-19 Pan-demic. JAMA Pediatr. 2021 Mar 1;175(3):313-316. doi: 10.1001/jamapediatrics.2020.5499.
Maddaloni et al., 2020	Maddaloni E, Coraggio L, Pieralice S, Carlone A, Pozzilli P, Buzzetti R. Effects of COVID-19 Lockdown on Glucose Control: Continuous Glucose Monitoring Data From People With Diabetes on Intensive Insulin Therapy. Diabetes Care. 2020 Aug;43(8):e86-e87. doi: 10.2337/dc20-0954.
Mamun et al., 2021	Mamun MA, Sakib N, Gozal D, Bhuiyan AI, Hossain S, Bodrud-Doza M, Al Mamun F, Hosen I, Safiq MB, Abdullah AH, Sarker MA, Rayhan I, Sikder MT, Muhit M, Lin CY, Griffiths MD, Pakpour AH. The COVID-19 pandemic and serious psychological consequences in Bangladesh: A population-based nationwide study. J Affect Disord. 2021 Jan 15;279:462-472. doi: 10.1016/j.jad.2020.10.036.
Meda et al., 2021	Meda N, Pardini S, Slongo I, Bodini L, Zordan MA, Rigobello P, Visioli F, Novara C. Students' mental health problems before, during, and after COVID-19 lockdown in Italy. J Psychiatr Res. 2021 Feb;134:69-77. doi: 10.1016/j.jpsychires.2020.12.045.

Study	Reference
Nilima et al., 2021	Nilima N, Kaushik S, Tiwary B, Pandey PK. Psycho-social factors associated with the nationwide lockdown in India during COVID- 19 pandemic. Clin Epidemiol Glob Health. 2021 Jan-Mar;9:47-52. doi: 10.1016/j.cegh.2020.06.010.
Nudelman et al., 2021	Nudelman G, Peleg S, Shiloh S. The Association Between Healthy Lifestyle Behaviours and Coronavirus Protective Behaviours. Int J Behav Med. 2021 Feb 10:1–9. doi: 10.1007/s12529-021-09960-6.
Owen et al., 2021	Owen AJ, Tran T, Hammarberg K, Kirkman M, Fisher J; COVID-19 Restrictions Impact Research Group. Poor appetite and overeating reported by adults in Australia during the coronavirus-19 disease pandemic: a population-based study. Public Health Nutr. 2021 Feb;24(2):275-281. doi: 10.1017/S1368980020003833.
Santos-García et al., 2020	Santos-García D, Oreiro M, Pérez P, Fanjul G, Paz González JM, Feal Painceiras MJ, Cores Bartolomé C, Valdés Aymerich L, García Sancho C, Castellanos Rodrigo MDM. Impact of Coronavirus Disease 2019 Pandemic on Parkinson's Disease: A Cross-Sectional Survey of 568 Spanish Patients. Mov Disord. 2020 Oct;35(10):1712-1716. doi: 10.1002/mds.28261.
Serafim et al., 2021	Serafim AP, Durães RSS, Rocca CCA, Gonçalves PD, Saffi F, Cappellozza A, Paulino M, Dumas-Diniz R, Brissos S, Brites R, Alho L, Lotufo-Neto F. Exploratory study on the psy-chological impact of COVID-19 on the general Brazilian population. PLoS One. 2021 Feb 3;16(2):e0245868. doi: 10.1371/journal.pone.0245868.
Serralta et al., 2020	Serralta FB, Zibetti MR, Evans C. Psychological Distress of University Workers during COVID-19 Pandemic in Brazil. Int J Environ Res Public Health. 2020 Nov 17;17(22):8520. doi: 10.3390/ijerph17228520.
Sutin et al., 2021	Sutin AR, Stephan Y, Luchetti M, Aschwanden D, Strickhouser JE, Lee JH, Sesker AA, Terracciano A. BMI, Weight Discrimination, and the Trajectory of Distress and Well-Being Across the Coronavirus Pandemic. Obesity (Silver Spring). 2021 Jan;29(1):38-45. doi: 10.1002/oby.23048.
Szczepanek, 2020	Szczepanek R. Analysis of pedestrian activity before and during COVID-19 lockdown, using webcam time-lapse from Cracow and machine learning. PeerJ. 2020 Oct 5;8:e10132. doi: 10.7717/peerj.10132.
Tasnim et al., 2020	Tasnim R, Islam MS, Sujan MSH, Sikder MT, Potenza MN. Suicidal ideation among Bangladeshi university students early during the COVID-19 pandemic: Prevalence estimates and correlates. Child Youth Serv Rev. 2020 Dec;119:105703. doi: 10.1016/j.childyouth.2020.105703.
Termorshuizen et al., 2020	Termorshuizen JD, Watson HJ, Thornton LM, Borg S, Flatt RE, MacDermod CM, Harper LE, van Furth EF, Peat CM, Bulik CM. Early impact of COVID-19 on individuals with self-reported eating disorders: A survey of ~1,000 individuals in the United States and the Netherlands. Int J Eat Disord. 2020 Nov;53(11):1780-1790. doi: 10.1002/eat.23353.
Zhang et al., 2020	Zhang M, Li Q, Du X, Zuo D, Ding Y, Tan X, Liu Q. Health Behavior Toward COVID-19: The Role of Demographic Factors, Knowledge, and Attitude Among Chinese College Stu-dents During the Quarantine Period. Asia Pac J Public Health. 2020 Nov;32(8):533-535. doi: 10.1177/1010539520951408.

S2.1.7 Excluded Fulltexts – Reason: Language

Study	Reference
Aajal et al., 2021	Aajal A, El Boussaadani B, Hara L, Benajiba C, Boukouk O, Benali M, Ouadfel O, Bendoudouch H, Zergoune N, Alkattan D, Mahdi Z, Najdi A, Raissuni Z. Les conséquences du confinement sur les maladies cardiovasculaires [The consequences of the lockdown on cardiovascular diseases]. Ann Cardiol Angeiol (Paris). 2021 Apr;70(2):94-101. French. doi: 10.1016/j.ancard.2021.01.006.
Alomo et al., 2020	Alomo M, Gagliardi G, Peloche S, Somers E, Alzina P, Prokopez CR. Efectos psicológicos de la pandemia COVID-19 en la población general de Argentina [Psychological effects during the COVID-19 outbreak in Argentina]. Rev Fac Cien Med Univ Nac Cordoba. 2020 Aug 25;77(3):176-181. Spanish. doi: 10.31053/1853.0605.v77.n3.28561.
Arevalo et al., 2020	Arevalo, harold & Urina, Miguel & Santacruz, Juan. (2020). Impact of mandatory preventive isolation on daily physical activity and weight of children in Colombia during the SARS-cov-2 pandemic. Revista Colombiana de Cardiologia. 10.1016/j.rccar.2020.09.003.
Durá-Travé, 2021	Durá-Travé T. Confinamiento domiciliario por COVID-19 y ganancia ponderal en escolares y adolescentes [Home confinement for COVID-19 and weight gain in schoolchildren and adolescents]. Nutr Hosp. 2021 Feb 23;38(1):213-214. Spanish. doi: 10.20960/nh.03417.
Fosco et al., 2020	Fosco MJ, Silva P, Taborda GA, Ahumada L. Asociación entre aislamiento social preventivo obligatorio por COVID-19 e hipertensión arterial grave [Association between mandatory lockdown due to COVID-19 and severe arterial hypertension]. Medicina (B Aires). 2020;80 Suppl 6:25-29.
Hartley et al., 2020	Hartley S, Colas des Francs C, Aussert F, Martinot C, Dagneaux S, Londe V, Waldron L, Royant-Parola S. Les effets de confinement SARS-CoV-2 sur le sommeil : enquête en ligne au cours de la quatrième semaine de confinement [The effects of quarantine for SARS-CoV-2 on sleep: An online survey]. Encephale. 2020 Jun;46(3S):S53-S59. French. doi: 10.1016/j.encep.2020.05.003.
Jauffret-Roustide et al., 2020	Jauffret-Roustide, M.; Barratt, M.; De Dinechin, S.; Davies, E.; Gilchrist, G.; Hughes, C.; Maier, L.; Ferris, J.; Winstock, A. Consumption of alcohol and other psychoactive drugs during the covid-19 pandemic in the global drug survey: A French perspective. Psychotropes (Belgium); 26(2-3):209-219, 2020.

Study	Reference
Kulig et al., 2020	Kulig B, Erdelyi-Hamza B, Elek LP, Kovacs I, Smirnova D, Fountoulakis K, Gonda X. A COVID-19 pandémia hatása a pszichológiai jóllétre, az életmódra és a járvány eredetével kapcsolatos attitűdökre pszichiátriai betegséggel élő és egészséges személyekben: egy nagy nemzetközi vizsgálat első magyarországi adatai [Effects of COVID-19 on psychological well-being, lifestyle and attitudes towards the origins of the pandemic in psychiatric patients and mentally healthy subjects: fi rst Hungarian descriptive results from a large international online study]. Neuropsychopharmacol Hung. 2020 Dec;22(4):154-165. Hungarian.
Perez-Rodrigo et al., 2020	Perez-Rodrigo, C., Citores, M. G., Barbara, G. H., Litago, F. R., Saenz, L. C., Aranceta-Bartrina, J., Val, V. A., Lopez-Sobaler, A. M., Victoria, E. M. D., Ortega, R. M., Partearroyo, T., Izquierdo, J. Q., Barba, L. R., Martin, A. R., Castell, G. S., Tur, J. A., Ibanez, M. V., Varela-Moreiras, G., Serra-Majem, L. Changes in eating habits during lockdown period due to the COVID-19 pandemic in Spain. [Spanish]. Revista Espanola de Nutricion Comunitaria 2020;26(2):101-11.
Rebollar Álvarez et al., 2021	Rebollar Álvarez A, Nuez Vicente C, Lozano Polo A, Pérez Ríos M, Pola Ferrández E, Furió Martínez AM, Monteagudo Piqueras O, Dalmau González-Gallarza R, Doncel Barrera J, Justo Gil S, Rey Brandariz J, González Carreño C, Gómez-Chacón Galán C. Consumo de tabaco en España durante el estado de alarma por COVID-19: resultados de una evalu-ación a través de redes sociales [Tobacco use in Spain during COVID-19 lockdown: an evaluation through social media.]. Rev Esp Salud Publica. 2021 Mar 16;95:e202103049.
Sánchez Conejero et al., 2021	Sánchez Conejero M, González de Buitrago Amigo J, Tejado Bravo ML, de Nicolás Jiménez JM. Repercusión del confinamiento por COVID-19 sobre el control glucémico en niños y adolescentes con diabetes mellitus tipo 1 [Impact of COVID-19 lockdown on glucemic control in children and adolescents with type 1 diabetes mellitus]. An Pediatr (Barc). 2021 Jan 23:S1695-4033(21)00003-5. Spanish. doi: 10.1016/j.anpedi.2020.12.021.
Sinisterra Loaiza et al., 2020	Sinisterra Loaiza LI, Vázquez Belda B, Miranda López JM, Cepeda A, Cardelle Cobas A. Hábitos alimentarios en la poblacion gallega durante el confinamiento por la COVID-19 [Food habits in the Galician population during confinement byCOVID-19]. Nutr Hosp. 2020 Dec 16;37(6):1190-1196. Spanish. doi: 10.20960/nh.03213.
Tárraga López et al., 2021	Tárraga López PJ, Panisello Royo JM, Carbayo Herencia JA, Carro A, Rosich N, Panisello M, Allins Presas J, Solera Albero J. Cambios observados en la adherencia a la dieta mediterránea en una población española durante el confinamiento debido a la pandemia ocasionada por el SARS-CoV-2 [Changes in adherence to the Mediterranean diet observed in a Spanish population during confinement for the SARS-CoV-2 pandemic]. Nutr Hosp. 2021 Feb 23;38(1):109-120. Spanish. doi: 10.20960/nh.03275.
Villanueva et al., 2021	Villanueva VJ, Motos P, Isorna M, Villanueva V, Blay P, Vázquez-Martínez A. Impacto de las medidas de confinamiento durante la pandemia de Covid-19 en el consumo de riesgo de alcohol [Impact of confinement measures on the Covid-19 pandemic on alcohol risk consumption.]. Rev Esp Salud Publica. 2021 Jan 20;95:e202101015.
Wiwanikit et al., 2020	Wiwanitkit V, Rodríguez-Núñez I. COVID-19, cuarentena, intensidad del ejercicio y niños [COVID-19, quarantine, exercise intensity and children]. Rev Chil Pediatr. 2020 Dec;91(6):1000. Spanish. doi: 10.32641/rchped.vi91i6.2991.

S2.1.8 Excluded fulltexts – Reason: Population

Study	Reference
Cavarretta et al., 2021	Cavarretta E, D'Angeli I, Giammarinaro M, Gervasi S, Fanchini M, Causarano A, Costa V, Manara M, Terribili N, Sciarra L, CalÒ L, Fossati C, Peruzzi M, Versaci F, Carnevale R, Biondi-Zoccai G, Frati G. Cardiovascular effects of COVID-19 lockdown in professional Football players. Panminerva Med. 2021 Feb 10. doi: 10.23736/S0031-0808.21.04340-8.

S2.1.9 Excluded fulltexts – Reason: Double publication

Study	Reference
Bourion-Bédès et al., 2021	Bourion-Bédès S, Tarquinio C, Batt M, Tarquinio P, Lebreuilly R, Sorsana C, Legrand K, Rousseau H, Baumann C. Stress and associated factors among French university students under the COVID-19 lockdown: The results of the PIMS-CoV 19 study. J Affect Disord. 2021 Mar 15;283:108-114. doi: 10.1016/j.jad.2021.01.041.

S2.2 Results of data extraction

<table>
<tr>
<th>Reference
(First author, publication year)</th>
<th>General
(Study design
Study name
Country of study
Time of study
No. of waves
Follow-up duration
(e.g., mean, range))</th>
<th>Population
(Description
Inclusion criteria
invited
baseline
follow-up
Age at baseline
% females
Response
Lost to follow-up)</th>
<th>Exposure
(Description & assessment)</th>
<th>Outcome
(Description & assessment)</th>
<th>Results
(Description of all topically relevant results)</th>
<th>Comments
(study quality [overall assessment according to Iljaz/Shamliyan/SIGN/CASP]
Conflict of interest [stated vs. not stated]
Funding (public, non-profit, commercial)
Strengths (+) and weaknesses (-))</th>
</tr>
<tr>
<td colspan="7">ADULTS FROM THE GENERAL POPULATION</td>
</tr>
<tr>
<td>Alpers et al., 2021</td>
<td>Study design:
Cross-sectional study
Study name:
n.a.
Country of study:
Norway
Time of study:
April 15–30, 2020
No. of waves:
n = 1
Follow-up duration:
n.a.</td>
<td>Description:
Adults
Inclusion criteria:
n.r.
# invited:
n = 81,170
# baseline:
n = 25,708
# follow-up:
n.a.
Age at baseline:
Median: 50 years
(IQR: 36–63)
% females:
56.2 %
Response:
31.7 %
Lost to follow-up:
n.a.</td>
<td>COVID-19 pandemic measures (implemented on March 12, 2020, social distancing, closure of educational, cultural, and training/sport/gym facilities, requirements to work from home, introduction of quarantine requirements):
- “placed in quarantine” (dichotomous variable: true/false)
- “temporarily laid-off” (dichotomous variable: true/false)
- “home office/study”, (dichotomous variable: true/false)</td>
<td>Alcohol consumption:
- Alcohol Use Disorders Identification Test Consumption (AUDIT-C) (score for hazardous drinking for women: ≥ 3, for men: ≥ 4)
- consumed mean units per week via units per drinking day and drinking days per month (frequent binge drinking: drinking > 6 units on one occasion at least once a month)
- (Change in) alcohol consumption (1 self-reported single question)</td>
<td>Tables 2 and S1: Descriptive statistics for the change in alcohol consumption since COVID-19 pandemic measures
<table>
<tr><th></th><th>Increase (%)</th><th>Decrease (%)</th></tr>
<tr><td colspan="3">Total sample</td></tr>
<tr><td></td><td>13</td><td>23</td></tr>
<tr><td colspan="3">Gender</td></tr>
<tr><td>Women</td><td>14</td><td>23</td></tr>
<tr><td>Men</td><td>13</td><td>22</td></tr>
<tr><td colspan="3">Age</td></tr>
<tr><td>18–29 years</td><td>16</td><td>48</td></tr>
<tr><td>30–39 years</td><td>20</td><td>25</td></tr>
<tr><td>40–49 years</td><td>16</td><td>21</td></tr>
<tr><td>50–59 years</td><td>12</td><td>19</td></tr>
<tr><td>60–69 years</td><td>10</td><td>15</td></tr>
<tr><td>≥ 70 years</td><td>6</td><td>15</td></tr>
</table>
Table 3: Risk factors for increase in alcohol consumption during COVID-19 pandemic phase
<table>
<tr><th></th><th>Unadjusted model
OR (95 % CI)</th><th>Adjusted model
OR (95 % CI)</th></tr>
<tr><td colspan="3">Temporarily lay-off</td></tr>
<tr><td></td><td>1.7 (1.5–2.0)</td><td>1.2 (1.0–1.4)</td></tr>
<tr><td colspan="3">Quarantine</td></tr>
<tr><td></td><td>1.2 (1.1–1.4)</td><td>1.2 (1.1–1.4)</td></tr>
<tr><td colspan="3">Home office/study</td></tr>
<tr><td></td><td>1.7 (1.5–1.9)</td><td>1.4 (1.3–1.6)</td></tr>
</table>
Table S4: Risk factors for decrease in alcohol consumption during COVID-19 pandemic phase
<table>
<tr><th></th><th>Unadjusted model
OR (95 % CI)</th><th>Adjusted model
OR (95 % CI)</th></tr>
<tr><td colspan="3">Temporarily laid-off</td></tr>
<tr><td></td><td>1.7 (1.6–1.9)</td><td>1.3 (1.1–1.4)</td></tr>
</table>
</td>
<td>Overall study quality:
High risk
Conflict of interest:
Stated (none declared)
Funding:
Public funds

+ ethical approval from the Regional Committee for Medical and Health Research Ethics, Health Region West
+ random sampling
+ validated outcome measurement for the association with the self-reported quarantine status
+ logistic regression analysis for the investigation of associations
+ consideration of confounders “sex” and “age” (by stratification and adjustment)

- low response and no non-responder analysis
- self-reported question for the change in alcohol consumption
- due to study design no actual chronological investigation</td>
</tr>
</table>

<table>
<tr>
<th>Reference
(First author, publication year)</th>
<th>General
(Study design
Study name
Country of study
Time of study
No. of waves
Follow-up duration
(e.g., mean, range))</th>
<th>Population
(Description
Inclusion criteria
invited
baseline
follow-up
Age at baseline
% females
Response
Lost to follow-up)</th>
<th>Exposure
(Description & assessment)</th>
<th>Outcome
(Description & assessment)</th>
<th>Results
(Description of all topically relevant results)</th>
<th>Comments
(study quality [overall assessment according to Iljaz/Shamliyan/SIGN/CASP]
Conflict of interest [stated vs. not stated]
Funding (public, non-profit, commercial)
Strengths (+) and weaknesses (-))</th>
</tr>
<tr>
<td></td>
<td></td>
<td></td>
<td></td>
<td></td>
<td><table>
<tr><td colspan="3">Quarantine</td></tr>
<tr><td></td><td>1.4 (1.2–1.5)</td><td>1.2 (1.1–1.4)</td></tr>
<tr><td colspan="3">Home office/study</td></tr>
<tr><td></td><td>1.9 (1.7–2.0)</td><td>1.4 (1.3–1.5)</td></tr>
</table>
Multivariable binary logistic regression
Adjusted model adjusted for age, gender, economic worries, health worries, temporarily lay-off and/or quarantine and/or home office/study</td>
<td></td>
</tr>
<tr>
<td>Avery et al., 2020</td>
<td>Study design:
Cross-sectional study
Study name:
Washington State Twin Registry
Country of study:
United States
Time of study:
March 26–April 5, 2020
No. of waves:
n = 1
Follow-up duration:
n.a.</td>
<td>Description:
Adult twins
Inclusion criteria:
Identical, same-sex fraternal twins
# invited:
n = 12,173
# baseline:
n = 3,971
# follow-up:
n.a.
Age at baseline:
M: 50.4 years (SD: 16.0)
% females:
69.2 %
Response:
Individual: 32.8 %
Pair-wise: 21.1 %
Lost to follow-up:
n.a.</td>
<td>COVID-19 mitigation strategies: Washington implemented the state-wide “stay home, stay healthy” order on March 24, 2020</td>
<td>(Change in) alcohol use:
1 self-reported single question</td>
<td>Table 1: Descriptive statistics for the change in alcohol use since lockdown
<table>
<tr><th></th><th>Prevalence (%)</th></tr>
<tr><td>Do not use</td><td>35.5</td></tr>
<tr><td>Use more</td><td>14.3</td></tr>
<tr><td>Use the same</td><td>39.4</td></tr>
<tr><td>Use less</td><td>10.9</td></tr>
</table></td>
<td>Overall study quality:
High risk
Conflict of interest:
Stated (none declared)
Funding:
Public funds

+ethical approval from the Institutional Review Board at Washington State University
+ recruitment in the Washington State Twin Registry (identification of twins by the Washington State Department of Licensing (DOL), researchers receive a list of potential twins from the DOL on a weekly basis, the register sends potential participants an enrolment survey, and twins are enrolled when surveys have been received from both members)

- low response and no non-responder analysis
- outcome measurement with a single self-reported question</td>
</tr>
</table>

<table>
<tr>
<th>Reference
(First author, publication year)</th>
<th>General
(Study design
Study name
Country of study
Time of study
No. of waves
Follow-up duration
(e.g., mean, range))</th>
<th>Population
(Description
Inclusion criteria
invited
baseline
follow-up
Age at baseline
% females
Response
Lost to follow-up)</th>
<th>Exposure
(Description & assessment)</th>
<th>Outcome
(Description & assessment)</th>
<th>Results
(Description of all topically relevant results)</th>
<th>Comments
(study quality [overall assessment according to Iljaz/Shamliyan/SIGN/CASP]
Conflict of interest [stated vs. not stated]
Funding (public, non-profit, commercial)
Strengths (+) and weaknesses (-))</th>
</tr>
<tr>
<td></td>
<td></td>
<td></td>
<td></td>
<td></td>
<td></td>
<td>- no consideration of the confounders “age” and “sex” in statistical analysis
- descriptive analysis only
- due to study design no actual chronological investigation</td>
</tr>
<tr>
<td>Berard et al., 2021</td>
<td>Study design:
Cross-sectional study
Study name:
PSYCOV-CV study
Country of study:
France
Time of study:
April 17–May 10, 2020
No. of waves:
n = 1
Follow-up duration:
n.a.</td>
<td>Description:
Older adults from the general population (aged ≥ 50 years)
Inclusion criteria:
n.r.
# invited:
n = 777
# baseline:
n = 536
# follow-up:
n.a.
Age at baseline:
M: 67 years (range: 50–89)
% females:
52 %
Response:
69 %
Lost to follow-up:
n.a.</td>
<td>COVID-19 lockdown (March 17–May 10, 2020):
- requirement to stay at home
- only reasons for going out: going to work (if teleworking was impossible); doing essential food shopping; travelling for health reasons, assisting vulnerable people, family emergencies, childcare; for individual physical activity, taking out a pet (within the limit of one hour per day and within a maximum radius of one kilometre around the home)
- prohibition of any gathering of people who did not live in the same home
- infringements to these rules were penalized initially by fines and further punishable by 6 months imprisonment</td>
<td>(Change in) diet quality:
Short, qualitative food frequency questionnaire (increased consumption of sugary foods, alcohol, fat or carbohydrates, not compensated by increased fruit and vegetable, dairy (within a limit of 2.5 servings/day), or lean protein consumption)
(Change in) physical activity (reduction of ≥ 15 minutes/week since the start of lockdown (as compared to the last 2–4 weeks before lockdown):
Self-reported question
(Change in) weight gain (≥ 500 g):
Self-reported question
(Change in) smoking (≥ 1 cigarette/day):
Self-reported question
(Change in) antihypertensive, lipid-lowering or hypoglycemic drug treatment:
Self-reported question</td>
<td>Table 1 and text: Descriptive statistics for the change in lifestyle factors since lockdown
<table>
<tr><th></th><th>Prevalence (n (%))</th></tr>
<tr><td>Decreased diet quality</td><td>142 (26.5)</td></tr>
<tr><td>Decreased physical activity</td><td>194 (36.2)</td></tr>
<tr><td>Weight gain</td><td>137 (25.6)</td></tr>
<tr><td>Increased smoking</td><td>21 (4.0)</td></tr>
<tr><td>Increased antihypertensive, lipid-lowering or hypoglycemic drug treatment</td><td>2 (0.37)</td></tr>
</table>
</td>
<td>Overall study quality:
High risk
Conflict of interest:
Stated (none declared)
Funding:
Public funds

+ random sampling
+ high response
+ outcome measurement of diet quality with a validated instrument

- self-reported outcome measurement of physical activity, weight gain, and smoking with single questions
- descriptive analysis only
- no consideration of the confounders “age” and “sex” in statistical analysis
- due to study design no actual chronological investigation</td>
</tr>
</table>

<table>
<tr>
<th>Reference
(First author, publication year)</th>
<th>General
(Study design
Study name
Country of study
Time of study
No. of waves
Follow-up duration
(e.g., mean, range))</th>
<th>Population
(Description
Inclusion criteria
invited
baseline
follow-up
Age at baseline
% females
Response
Lost to follow-up)</th>
<th>Exposure
(Description & assessment)</th>
<th>Outcome
(Description & assessment)</th>
<th>Results
(Description of all topically relevant results)</th>
<th>Comments
(study quality [overall assessment according to Iljaz/Shamliyan/SIGN/CASP]
Conflict of interest [stated vs. not stated]
Funding (public, non-profit, commercial)
Strengths (+) and weaknesses (-))</th>
</tr>
<tr>
<td>Cicero et al., 2021</td>
<td>Study design:
Cross-sectional study
Study name:
Brisighella Heart Study
Country of study:
Italy
Time of study:
n.r.
No. of waves:
n = 1
Follow-up duration:
n.a.</td>
<td>Description:
Adults
Inclusion criteria:
Exclusion of workers not in quarantine (e.g., healthcare personnel, police forces, military personnel and farmers), if they were in quarantine from less than four weeks, or were affected by COVID-19 infection or other severe diseases diagnosed after the last population survey
# invited:
n = 1,538
# baseline:
n = 359
# follow-up:
n.a.
Age at baseline:
M: 64.6 years (SD: 13.3)
% females:
56.5 %
Response:
23.3 %
Lost to follow-up:
n.a.</td>
<td>COVID-19-related quarantine
(February–April 2020)</td>
<td>Dietary quality:
Dietary Quality Index
Alcohol consumption:
1 item from the Dietary Quality Index
Smoking: Single question
Body mass index: Single question

Retrospective assessment of levels pre-quarantine using the same instruments</td>
<td>Text: Change in energy intake (kcal) and overall dietary quality index since lockdown
<table>
<tr><th></th><th>Pre-quarantine (M (SD))</th><th>During quarantine (M (SD))</th><th>p</th></tr>
<tr><td>Energy intake</td><td>2568 (322)</td><td>2739 (442)</td><td>< 0.001</td></tr>
<tr><td>Dietary quality index</td><td>42.4 (4.1)</td><td>37.8 (4.7)</td><td>0.011</td></tr>
</table>
Text: Change in daily consumption of different food categories since lockdown
<table>
<tr><th></th><th>Direction of change</th><th>p</th></tr>
<tr><td colspan="3">Daily intake</td></tr>
<tr><td>Bread and bread-like products</td><td>↑</td><td>< 0.001</td></tr>
<tr><td>Pasta and rice</td><td>↑</td><td>< 0.001</td></tr>
<tr><td>Green vegetable</td><td>↑</td><td>< 0.001</td></tr>
<tr><td>Other vegetables</td><td>↑</td><td>< 0.001</td></tr>
<tr><td>Healthy vegetable oils</td><td>↑</td><td>< 0.001</td></tr>
<tr><td>Milk and yogurt</td><td>↑</td><td>< 0.001</td></tr>
<tr><td>Simple sugars and sweets</td><td>↑</td><td>< 0.001</td></tr>
<tr><td>Coffee</td><td>↑</td><td>< 0.001</td></tr>
<tr><td>Non-alcoholic drinks</td><td>=</td><td>0.778</td></tr>
<tr><td>Water</td><td>=</td><td>0.69</td></tr>
<tr><td colspan="3">Frequent weekly intake (0–6 portion per week)</td></tr>
<tr><td>Low-fat meat</td><td>↑</td><td>< 0.001</td></tr>
<tr><td>Cured meats other than ham</td><td>↑</td><td>< 0.001</td></tr>
<tr><td>Cheeses</td><td>↑</td><td>< 0.001</td></tr>
<tr><td>Eggs</td><td>↑</td><td>0.015</td></tr>
<tr><td>Mixed seed oils</td><td>↑</td><td>< 0.001</td></tr>
<tr><td>Non-cured fat meat</td><td>=</td><td>0.954</td></tr>
<tr><td>Ham</td><td>=</td><td>0.835</td></tr>
<tr><td colspan="3">Less frequent week intake (0–3 portion per week)</td></tr>
</table></td>
<td>Overall study quality:
High risk
Conflict of interest:
Stated (none declared)
Funding:
Bank foundation

+ ethical approval from the Institutional Ethical Board of the University Hospital of Bologna
+ random sampling of the participants of the original study
+ measurement of dietary quality with a validated instrument
+ using t-tests for paired samples or the Wilcoxon rank test to investigate differences
+ consideration of confounders “sex” and “age” (by stratification)

- low response
- measurement of smoking and body mass index with single questions
- retrospective assessment of energy intake, main diet components, alcohol consumption, and body mass index before lockdown (recall bias)
- due to study design no actual chronological investigation</td>
</tr>
</table>

**Reference** (First author, publication year)	**General** (Study design Study name Country of study Time of study No. of waves Follow-up duration (e.g., mean, range))	**Population** (Description Inclusion criteria # invited # baseline # follow-up Age at baseline % females Response Lost to follow-up)	**Exposure** (Description & assessment)	**Outcome** (Description & assessment)	**Results** (Description of all topically relevant results)	**Comments** (study quality [overall assessment according to Iljaz/Shamliyan/SIGN/CASP] Conflict of interest [stated vs. not stated] Funding (public, non-profit, commercial) Strengths (+) and weaknesses (-))
					(see nested tables below)	

Fish	↓	< 0.001
Mussels and shellfish	↓	< 0.001
Legumes	↓	< 0.001
Nuts	↑	0.042

Table 2: Percentage of total energy derived from the main diet components

	**Pre-quarantine** (% (SD))	**During quarantine** (% (SD))	**p**
Total carbo-hydrates	49.3 (4.6)	52.6 (6.5)	0.048
Simple sugars	3.1 (0.9)	4.6 (1.1)	0.002
Total fats	28.1 (3.2)	31.4 (2.9)	0.047
Added fats	3.9 (1.1)	4.3 (1.2)	0.021
Proteins	16.2 (2.6)	10.1 (2.7)	0.003

Table 2: Percentage of total energy derived from the alcohol

	**Pre-quarantine** (% (SD))	**During quarantine** (% (SD))	**p**
Alcohol	2.9 (0.6)	4.9 (1.0)	0.002

Text: Descriptive statistics for the change in smoking since lockdown

	**Prevalence** (%)
Reduction	2.2
Increase	1.7

Text: Change in body mass index (kg/m^2) since lockdown

	**Pre-quarantine** (M (SD))	**During quarantine** (M (SD))	**p**

<table>
<tr>
<th>Reference
(First author, publication year)</th>
<th>General
(Study design
Study name
Country of study
Time of study
No. of waves
Follow-up duration
(e.g., mean, range))</th>
<th>Population
(Description
Inclusion criteria
invited
baseline
follow-up
Age at baseline
% females
Response
Lost to follow-up)</th>
<th>Exposure
(Description & assessment)</th>
<th>Outcome
(Description & assessment)</th>
<th>Results
(Description of all topically relevant results)</th>
<th>Comments
(study quality [overall assessment according to Iljaz/Shamliyan/SIGN/CASP]
Conflict of interest [stated vs. not stated]
Funding (public, non-profit, commercial)
Strengths (+) and weaknesses (-))</th>
</tr>
<tr>
<td></td>
<td></td>
<td></td>
<td></td>
<td></td>
<td><table><tr><td>Body mass index</td><td>26.6 (4.7)</td><td>26.9 (4.5)</td><td>0.361</td></tr></table>Results were confirmed by repeating the analysis by gender, education level, and age class.</td>
<td></td>
</tr>
<tr>
<td>Colley et al., 2020</td>
<td>Study design:
Cross-sectional study
Study name:
Canadian Perspective Survey Series – Impacts of COVID-19
(CPSS1)
Country of study:
Canada
Time of study:
March 29–April 3, 2020
No. of waves:
n = 1
Follow-up duration:
n.a.</td>
<td>Description:
Adults (1 household member selected at random from four Labour Force Survey (LFS) groups that answered the LFS for the last time in 2019)
Inclusion criteria:
Canadian residents aged ≥ 20 years, living in the 10 provinces, exclusion of persons living on reserves and Aboriginal settlements in the provinces; full-time members of the Canadian Armed Forces; the institutionalized population; and households in extremely remote areas with very low population density
# invited:
n = 7,242
# baseline:
n = 4,524
# follow-up:
n.a.
Age at baseline:
n.r.
% females:
53.4 %</td>
<td>Physical distancing measures (implemented in March 2020):
- border, school, and business closures
- avoiding unnecessary trips</td>
<td>Screen time behaviours:
3 self-reported single questions (“Have your weekly habits changed for any of the following activities?”: watching TV, spending time on the internet, playing video games)</td>
<td>Table 1: Descriptive statistics for an increase in watching TV
<table>
<tr><th></th><th>Prevalence (% (95% CI))</th></tr>
<tr><td colspan="2">Overall</td></tr>
<tr><td>Men</td><td>59.8 (56.3–63.2)</td></tr>
<tr><td>Women</td><td>66.0 (63.2–68.6)</td></tr>
<tr><td colspan="2">20–34 years</td></tr>
<tr><td>Men</td><td>59.6 (50.4–68.2)</td></tr>
<tr><td>Women</td><td>72.7 (66.2–78.3)</td></tr>
<tr><td colspan="2">35–54 years</td></tr>
<tr><td>Men</td><td>61.8 (56.2–67.2)</td></tr>
<tr><td>Women</td><td>68.1 (63.6–72.4)</td></tr>
<tr><td colspan="2">≥ 55 years</td></tr>
<tr><td>Men</td><td>58.1 (53.4–62.7)</td></tr>
<tr><td>Women</td><td>60.2 (55.7–64.5)</td></tr>
</table>
Table 1: Descriptive statistics for an increase in spending time on the internet
<table>
<tr><th></th><th>Prevalence (% (95% CI))</th></tr>
<tr><td colspan="2">Overall</td></tr>
<tr><td>Men</td><td>63.5 (60.2–66.6)</td></tr>
<tr><td>Women</td><td>68.7 (65.6–71.6)</td></tr>
<tr><td colspan="2">20–34 years</td></tr>
<tr><td>Men</td><td>75.8 (67.6–82.5)</td></tr>
<tr><td>Women</td><td>74.8 (67.5–80.9)</td></tr>
<tr><td colspan="2">35–54 years</td></tr>
<tr><td>Men</td><td>62.9 (57.2–68.2)</td></tr>
<tr><td>Women</td><td>74.2 (69.8–78.3)</td></tr>
<tr><td colspan="2">≥ 55 years</td></tr>
<tr><td>Men</td><td>55.9 (51.1–60.7)</td></tr>
<tr><td>Women</td><td>60.5 (55.8–65.1)</td></tr>
</table></td>
<td>Overall study quality:
High risk
Conflict of interest:
Unclear
Funding:
Unclear

+ random sampling
+ high response
+ consideration of confounders “sex” and “age” (by stratification)

- outcome measurement with self-reported single questions
- descriptive analysis only
- due to study design no actual chronological investigation</td>
</tr>
</table>

<table>
<tr>
<th>Reference
(First author, publication year)</th>
<th>General
(Study design
Study name
Country of study
Time of study
No. of waves
Follow-up duration
(e.g., mean, range))</th>
<th>Population
(Description
Inclusion criteria
invited
baseline
follow-up
Age at baseline
% females
Response
Lost to follow-up)</th>
<th>Exposure
(Description & assessment)</th>
<th>Outcome
(Description & assessment)</th>
<th>Results
(Description of all topically relevant results)</th>
<th>Comments
(study quality [overall assessment according to Iljaz/Shamliyan/SIGN/CASP]
Conflict of interest [stated vs. not stated]
Funding (public, non-profit, commercial)
Strengths (+) and weaknesses (-))</th>
</tr>
<tr>
<td></td>
<td></td>
<td>Response:
62.5 %
Lost to follow-up:
n.a.</td>
<td></td>
<td></td>
<td>Table 1: Descriptive statistics for an increase in playing video games
<table>
<tr><th></th><th>Prevalence (% (95% CI))</th></tr>
<tr><td colspan="2">Overall</td></tr>
<tr><td>Men</td><td>24.1 (21.1–27.3)</td></tr>
<tr><td>Women</td><td>16.0 (13.6–18.6)</td></tr>
<tr><td colspan="2">20–34 years</td></tr>
<tr><td>Men</td><td>50.6 (42.3–58.9)</td></tr>
<tr><td>Women</td><td>29.4 (23.1–36.6)</td></tr>
<tr><td colspan="2">35–54 years</td></tr>
<tr><td>Men</td><td>21.3 (17.5–25.6)</td></tr>
<tr><td>Women</td><td>15.5 (11.8–19.9)</td></tr>
<tr><td colspan="2">≥ 55 years</td></tr>
<tr><td>Men</td><td>7.2 (5.1–10.0)</td></tr>
<tr><td>Women</td><td>7.3 (5.5–9.7)</td></tr>
</table>
Table 1: Descriptive statistics for an increase in 2 or 3 types of screen time behaviors
<table>
<tr><th></th><th>Prevalence (% (95% CI))</th></tr>
<tr><td colspan="2">Overall</td></tr>
<tr><td>Men</td><td>52.7 (49.1–56.2)</td></tr>
<tr><td>Women</td><td>56.7 (53.4–60.0)</td></tr>
<tr><td colspan="2">20–34 years</td></tr>
<tr><td>Men</td><td>63.9 (54.9–72.1)</td></tr>
<tr><td>Women</td><td>67.8 (60.7–74.3)</td></tr>
<tr><td colspan="2">35–54 years</td></tr>
<tr><td>Men</td><td>53.6 (48.2–58.9)</td></tr>
<tr><td>Women</td><td>60.0 (55.0–64.8)</td></tr>
<tr><td colspan="2">≥ 55 years</td></tr>
<tr><td>Men</td><td>43.3 (38.2–48.6)</td></tr>
<tr><td>Women</td><td>46.3 (41.2–51.4)</td></tr>
</table></td>
<td></td>
</tr>
<tr>
<td>Crochemore -Silva et al., 2020</td>
<td>Study design:
Cross-sectional study
Study name:
n.a.
Country of study:</td>
<td>Description:
Adults
Inclusion criteria:
Residents of Bagé’s urban area aged ≥ 18 years</td>
<td>Social distancing (public calamity had been declared (municipal decree 050/2020) in the municipality since March 19, 2020, with the adoption of strict social distancing</td>
<td>Leisure-time physical activity:
1 item from an adapted version of the International Physical Activity Questionnaire:</td>
<td>Figure 3 (A) and text: Percentage of those engaging in physical activities
<table>
<tr><th>Level of social distancing</th><th>Prevalence (%)</th><th>p</th></tr>
<tr><td>Very little</td><td>~ 20</td><td rowspan="2">0.023</td></tr>
<tr><td>Little</td><td>n.r. (~ 21*)</td></tr>
</table></td>
<td>Overall study quality:
High risk
Conflict of interest:
Unclear
Funding:
Unclear</td>
</tr>
</table>

<table>
<tr>
<th>Reference
(First author, publication year)</th>
<th>General
(Study design
Study name
Country of study
Time of study
No. of waves
Follow-up duration
(e.g., mean, range))</th>
<th>Population
(Description
Inclusion criteria
invited
baseline
follow-up
Age at baseline
% females
Response
Lost to follow-up)</th>
<th>Exposure
(Description & assessment)</th>
<th>Outcome
(Description & assessment)</th>
<th>Results
(Description of all topically relevant results)</th>
<th>Comments
(study quality [overall assessment according to Iljaz/Shamliyan/SIGN/CASP]
Conflict of interest [stated vs. not stated]
Funding (public, non-profit, commercial)
Strengths (+) and weaknesses (-))</th>
</tr>
<tr>
<td></td>
<td>Brazil
Time of study:
May 7–9, 2020
No. of waves:
n = 1
Follow-up duration:
n.a.</td>
<td># invited:
n = 400
# baseline:
n = 377
# follow-up:
n.a.
Age at baseline:
n.r.
% females:
62.9 %
Response:
94.3 %
Lost to follow-up:
n.a.</td>
<td>measures, where only essential activities in force remained open; decrees 58 and 59/2020 (published on April 15 and 30, 2020, respectively) were in effect during data collection (suspension of activities in the education network (public and private), social and sports clubs, gyms, cinemas, and bars, amongst others):
2 self-reported single questions from the EPICOVID-RS study:
1. level of “the social distancing instructed by health authorities, that is, staying at home and avoiding contact with other people”, how much they considered they were managing to do it” (response categories: very little; little; average; a lot; practically isolated from everyone)
2. activity routine (response categories: stayed at home all the time; went out only for essential things like buying food; went out from time to time to shop and stretch their legs; went out every day for some activity; went out every day, all day, to work or to perform other regular activities)</td>
<td>percentage of those engaging in physical activities such as gymnastics, sports, hiking, or jogging during the past seven days</td>
<td><table>
<tr><td>Average</td><td>37.7</td><td></td></tr>
<tr><td>A lot</td><td>n.r. (~ 25*)</td><td></td></tr>
<tr><td>Virtually isolated</td><td>~ 20</td><td></td></tr>
</table>
Figure 3 (B) and text: Percentage of those engaging in physical activities
<table>
<tr><th>Activity routine</th><th>Prevalence (%)</th><th>p</th></tr>
<tr><td>Staying home</td><td>12.5</td><td>0.014</td></tr>
<tr><td>Leaves home for essential purchases</td><td>n.r. (~ 27*)</td><td></td></tr>
<tr><td>Leaves home sometimes</td><td>n.r. (~ 35*)</td><td></td></tr>
<tr><td>Leaves home every day</td><td>40.7</td><td></td></tr>
<tr><td>Leaves home every day (for work)</td><td>27.0</td><td></td></tr>
</table>
Chi-square test of heterogeneity and linear trend
* Estimation by the review authors by reading the bars of figure 3.</td>
<td>+ ethical approval from the Research Ethics Committee of the Federal University of Pelotas
+ multiple-stage process (random selection of census tracts, households, and household individuals)
+ high response
+ using Chi-square test to investigate differences

- self-reported exposure measurement with 2 single questions
- self-reported outcome measurement with a single item
- no consideration of the confounders “age” and “sex” in statistical analysis
- due to study design no actual chronological investigation</td>
</tr>
<tr>
<td>Daly & Robinson, 2021</td>
<td>Study design:
Cohort study
Study name:
1970 British Cohort Study</td>
<td>Description:
Adults born in Britain in 1970
Inclusion criteria:
baseline:</td>
<td>COVID-19 lockdown restrictions:
- pubs, bars, and restaurants and other nonessential businesses were closed for</td>
<td>High risk alcohol consumption:
Alcohol Use Disorders Identification Test (AUDIT) for detecting</td>
<td>Text: Overall AUDITPC score
<table>
<tr><th></th><th>2016–2018 (M (SD))</th><th>May 2020 (M (SD))</th><th>p</th></tr>
</table></td>
<td>Overall study quality:
High risk
Conflict of interest:
Unclear
Funding:</td>
</tr>
</table>

<table>
<tr><th>Reference
(First author, publication year)</th><th>General
(Study design
Study name
Country of study
Time of study
No. of waves
Follow-up duration
(e.g., mean, range))</th><th>Population
(Description
Inclusion criteria
invited
baseline
follow-up
Age at baseline
% females
Response
Lost to follow-up)</th><th>Exposure
(Description & assessment)</th><th>Outcome
(Description & assessment)</th><th>Results
(Description of all topically relevant results)</th><th>Comments
(study quality [overall assessment according to Iljaz/Shamliyan/SIGN/CASP]
Conflict of interest [stated vs. not stated]
Funding (public, non-profit, commercial)
Strengths (+) and weaknesses (-))</th></tr>
<tr><td></td><td>Country of study:
United Kingdom
Time of study:
Baseline: 2016–2018
Follow-up: May 2020
No. of waves:
n = 2
Follow-up duration:
Range: 2–4 years</td><td>n.r.
# invited at follow-up:
n = 10,458
# follow-up:
n = 3,358 (those who took part at baseline and at follow-up)
Age at baseline:
Range: 46–48 years
% females:
50.0 %
Response (at follow-up):
32.1 %
Lost to follow-up:
n.r.</td><td>> 3 months between late March and early July 2020</td><td>hazardous drinkers in primary care settings (AUDITPC)
- for the COVID-19 survey, the AUDIT-PC was adapted by adding Since the start of the Coronavirus outbreak to the start of each item
- High risk score: ≥ 5</td><td><table><tr><td>Total sample</td><td>3.17 (2.46)</td><td>3.34 (2.77)</td><td>0.003</td></tr></table>
Table 1: Prevalence of high-risk drinking<table><tr><th></th><th>2016–2018 (%)</th><th>May 2020 (%)</th><th>Difference (% (95 % CI))</th></tr><tr><td>Total sample</td><td>19.3</td><td>24.6</td><td>5.2 (2.4–8.0) (p < 0.001)</td></tr><tr><td>Men</td><td>24.7</td><td>29.9</td><td>5.2 (0.5–10.1) (p < 0.001)</td></tr><tr><td>Women</td><td>14.0</td><td>19.2</td><td>5.2 (2.3–8.1) (p < 0.001)</td></tr></table>Logistic regression analyses
Adjusted for sex, marital status, educational qualifications, diagnosis of a chronic mental or physical health condition, year when the 2016−2018 baseline survey was completed</td><td>Public funds

+ ethical approval from the National Research Ethics Service
+ validated outcome measure
+ using logistic regression analyses to investigate differences
+ statistical analysis of confounders “sex” (adjustment) and “age” (homogeneous group)
+ temporal investigation

- no information on lost to follow-up and number of participants at baseline</td></tr>
<tr><td>Duncan et al., 2020</td><td>Study design:
Cross-sectional study
Study name:
Washington State Twin Registry
Country of study:
United States
Time of study:
March 26–April 5, 2020
No. of waves:
n = 1
Follow-up duration:
n.a.</td><td>Description:
Adult Twins
Inclusion criteria:
Identical, same-sex fraternal twins
# invited:
n = 12,173
# baseline:
n = 3,971
# follow-up:
n.a.
Age at baseline:
M: 50.4 years (SD: 16.0)
% females:
69.2 %
Response:
Individual: 32.8 %
Pair-wise: 21.1 %
Lost to follow-up:</td><td>COVID-19 mitigation strategies:
Washington implemented the state-wide “stay home, stay healthy” order on March 24, 2020</td><td>(Change in) physical activity:
1 self-reported single question</td><td>Table 1 and text: Descriptive statistics for the change in physical activity compared to one month ago (i.e., prior to the spread of COVID-19)<table><tr><th></th><th>Prevalence (%)</th></tr><tr><td>Total sample</td><td></td></tr><tr><td>Decreased a lot</td><td>15.1</td></tr><tr><td>Decreased somewhat</td><td>28.7</td></tr><tr><td>No change</td><td>26.4</td></tr><tr><td>Increased a lot</td><td>5.2</td></tr><tr><td>Increased somewhat</td><td>21.2</td></tr><tr><td>Men</td><td></td></tr><tr><td>Decreased (a lot or somewhat)</td><td>45.3</td></tr><tr><td>No change</td><td>29.8</td></tr><tr><td>Increased (a lot or somewhat)</td><td>25.5</td></tr><tr><td>Women</td><td></td></tr><tr><td>Decreased (a lot or somewhat)</td><td>41.5</td></tr><tr><td>No change</td><td>31.1</td></tr><tr><td>Increased (a lot or somewhat)</td><td>27.4</td></tr></table></td><td>Overall study quality:
High risk
Conflict of interest:
Stated (none declared)
Funding:
Public funds

+ethical approval from the Institutional Review Board at Washington State University
+ recruitment in the Washington State Twin Registry (identification of twins by the Washington State Department of Licensing (DOL), researchers receive a list of potential twins from the DOL on a weekly basis, the register sends potential participants an enrolment survey,</td></tr>
</table>

**Reference** (First author, publication year)	**General** (Study design Study name Country of study Time of study No. of waves Follow-up duration (e.g., mean, range))	**Population** (Description Inclusion criteria # invited # baseline # follow-up Age at baseline % females Response Lost to follow-up)	**Exposure** (Description & assessment)	**Outcome** (Description & assessment)	**Results** (Description of all topically relevant results)	**Comments** (study quality [overall assessment according to Iljaz/Shamliyan/SIGN/CASP] Conflict of interest [stated vs. not stated] Funding (public, non-profit, commercial) Strengths (+) and weaknesses (-))
		n.a.				and twins are enrolled when surveys have been received from both members)<br>+ stratification of the results for the confounder "sex"<br><br>- low response and no non-responder analysis<br>- outcome measurement with a self-reported single question<br>- no consideration of the confounder "age" in the statistical analysis<br>- descriptive analysis only<br>- due to study design no actual chronological investigation
Garre-Olmo et al., 2020	**Study design:**<br>Cross-sectional study<br>**Study name:**<br>Girona Healthy Region Study<br>**Country of study:**<br>Spain<br>**Time of study:**<br>April 8–May 4, 2020<br>**No. of waves:**<br>*n* = 1<br>**Follow-up duration:**<br>n.a.	**Description:**<br>Adults from the general population<br>**Inclusion criteria:**<br>Residents living in the province of Girona (Catalonia), Spain, aged ≥ 18 years, exclusion of subjects with terminal illness, cognitive impairment or dementia, intellectual disability, or institutionalized<br>**# invited:**<br>*n* = 765<br>**# baseline:**<br>*n* = 692<br>**# follow-up:**<br>n.a.<br>**Age at baseline:**	**Movement restrictions and confinement due to the COVID-19 pandemic** (state of alarm implemented by the Spanish government on March 15, 2020):<br>- suspension of all academic activities<br>- Spanish citizens were obliged to stay at home except to purchase food and medicines, to go to work, or to attend emergencies<br>(- more restrictive lockdown period that included the temporary closure of all the non-essential activities and businesses between March 29 and April 9, 2020)	**(Change in) physical activity:**<br>1 self-reported single question<br>**(Change in) dietary pattern:**<br>1 self-reported single question	**Table 2: Characteristics of study participants regarding confinement consequences**<br><table><tr><th></th><th>N (%)</th></tr><tr><td>Worsening dietary pattern</td><td>134 (19.4)</td></tr><tr><td>Diminished sport practice</td><td>420 (60.7)</td></tr></table>	**Overall study quality:**<br>High risk<br>**Conflict of interest:**<br>Stated (none declared)<br>**Funding:**<br>Public funds<br><br>+ ethical approval from Ethics Committee of the IDIAP Jordi Gol<br>+ ratified random sampling procedure according to the population structure<br>+ high response<br>+ non-responder analysis (no differences by age or gender between participants and persons who refused to participate)

<table>
<tr>
<th>Reference
(First au-
thor, publi-
cation year)</th>
<th>General
(Study design
Study name
Country of study
Time of study
No. of waves
Follow-up duration
(e.g., mean, range))</th>
<th>Population
(Description
Inclusion criteria
invited
baseline
follow-up
Age at baseline
% females
Response
Lost to follow-up)</th>
<th>Exposure
(Description & assessment)</th>
<th>Outcome
(Description & assess-
ment)</th>
<th>Results
(Description of all topically relevant results)</th>
<th>Comments
(study quality [overall as-
sessment according to
Iljaz/Shamliyan/SIGN/CASP]
Conflict of interest [stated
vs. not stated]
Funding (public, non-profit,
commercial)
Strengths (+) and weak-
nesses (-))</th>
</tr>
<tr>
<td></td>
<td></td>
<td>M: 50.2 years (SD:
16.3, range: 18.9–
90.9)
% females:
54.8 %
Response:
90.5 %
Lost to follow-up:
n.a.</td>
<td></td>
<td></td>
<td></td>
<td>- outcome measurement
with self-reported single
questions
- descriptive analysis only
- no consideration of the
confounders “age” and “sex”
in the statistical analysis
- due to study design no ac-
tual chronological investiga-
tion</td>
</tr>
<tr>
<td>Karuc et al.,
2020</td>
<td>Study design:
Cross-sectional
study
Study name:
Croatian physical
activity in adoles-
cence longitudinal
study (CRO-PALS)
Country of study:
Croatia
Time of study:
April 24–May 8,
2020
No. of waves:
n = 1
Follow-up dura-
tion:
n.a.</td>
<td>Description:
Young adults
Inclusion criteria:
n.r.
# invited:
n = 363
# baseline:
n = 91
# follow-up:
n.a.
Age at baseline:
Females: M:
21.6 years (SD: 0.4)
Males: M: 21.5 years
(SD: 0.3)
% females:
64.8 %
Response:
25.1 %
Lost to follow-up:
n.a.</td>
<td>Restrictions due to COVID-
19 Pandemic (March 19th–
May 11, 2020):
- restricting gathering in public
places and parks
- suspension of public trans-
portation, and close institu-
tions
- prohibition of all social gath-
erings, work in retail and ser-
vices including sports activities</td>
<td>Physical activity:
- 7-day recall of moder-
ate intensity physical ac-
tivity (MPA) and vigor-
ous intensity physical
activity (VPA): School
Health Action, Planning,
and Evaluation System
(SHAPES) questionnaire
- (Change in) physical
activity: self-reported
single question

Retrospective assess-
ment of levels pre-re-
strictions using the same
instruments</td>
<td>Table 1: Moderate-to-vigorous physical activity (minutes/day) since restrictions
<table>
<tr><th></th><th>Pre-re-
strictions
(Median
(IQR))</th><th>Post-re-
strictions
(Median
(IQR))</th><th>p</th></tr>
<tr><td>Females</td><td>120.0
(227.1)</td><td>64.3 (75.0)</td><td>> 0.0001</td></tr>
<tr><td>Males</td><td>135.0
(127.5)</td><td>85.7 (56.8)</td><td>0.006</td></tr>
</table>
ANOVA
- no interaction between sex and time (p = 0.79)

Table 1: Descriptive statistics in changes of physical activity since restrictions
<table>
<tr><th></th><th>Prevalence (%)</th></tr>
<tr><td>Females</td><td></td></tr>
<tr><td>No change</td><td>25</td></tr>
<tr><td>Increase</td><td>19</td></tr>
<tr><td>Decrease</td><td>56</td></tr>
<tr><td>Males</td><td></td></tr>
<tr><td>No change</td><td>31</td></tr>
<tr><td>Increase</td><td>19</td></tr>
<tr><td>Decrease</td><td>50</td></tr>
</table></td>
<td>Overall study quality:
High risk
Conflict of interest
Stated (none declared)
Funding:
Public funds

+ ethical approval from the
Ethics Committee of the
Faculty of Kinesiology of the
University of Zagreb
+ stratified two-stage ran-
dom sampling procedures
(school level and class level)
of the original study
+ validated outcome meas-
urement for the investigation
of the association between
quarantine status and physi-
cal activity
+ using ANOVA to investi-
gate differences
+ statistical analysis of con-
founders “sex” (by stratifica-
tion and interaction analysis)
and “age” (homogeneous
group)

- low response</td>
</tr>
</table>

<table>
<tr><th>Reference
(First author, publication year)</th><th>General
(Study design
Study name
Country of study
Time of study
No. of waves
Follow-up duration
(e.g., mean, range))</th><th>Population
(Description
Inclusion criteria
invited
baseline
follow-up
Age at baseline
% females
Response
Lost to follow-up)</th><th>Exposure
(Description & assessment)</th><th>Outcome
(Description & assessment)</th><th>Results
(Description of all topically relevant results)</th><th>Comments
(study quality [overall assessment according to Iljaz/Shamliyan/SIGN/CASP]
Conflict of interest [stated vs. not stated]
Funding (public, non-profit, commercial)
Strengths (+) and weaknesses (-))</th></tr>
<tr><td></td><td></td><td></td><td></td><td></td><td></td><td>- difference between responders and non-responders regarding age, sex, socio-economic status, and moderate-to-vigorous physical activity
- retrospective assessment of activity levels, before lockdown (recall bias)
- self-reported question for the outcome “change in physical activity”
- due to study design no actual chronological investigation</td></tr>
<tr><td>Mason et al., 2020</td><td>Study design:
Cohort study
Study name:
Happiness and Health prospective cohort study
Country of study:
United States
Time of study:
Baseline (wave 9): October 2018–October 2019
Follow-up (wave 10): May–July 2020
No. of waves:
n = 2
Follow-up duration:
About 1.5 years</td><td>Description:
Young adults
Inclusion criteria:
n.r.
invited:
Wave 1 (2013):
n = 4,100
Wave 10 (2020):
n = 2,548
# baseline:
Baseline (wave 9): n.r.
# follow-up:
n = 1,820
Age at baseline:
M: 19.72 years (SD: 0.46)
% females:
61.5 %
Response:
Wave 1 (2013):
82.5 %
Wave 10 (2020):
71.4 %
Lost to follow-up:</td><td>COVID-19 restrictions:
- modified on May 4, 2020
- residents were still directed to stay at home except for essential needs
- but lower-risk businesses and spaces were allowed to reopen (“Stage two”, May 4, 2020)
- counties could selectively reopen higher-risk businesses and spaces /“Stage three”, May 18, 2020)
- air service businesses could reopen with restrictions (May 26, 2020)
- closure of most indoor businesses in many counties due to increase in cases (June and July 2020)
- closure of gyms, indoor dining, bars, movie theatres, and museums (July 13, 2020)</td><td>Weight:
Self-reported single question</td><td>Text: Descriptive statistics in weight change (pounds (lbs.)) since restrictions
<table><tr><th></th><th>M (SD)</th><th>M% (SD)</th></tr><tr><td>Weight change</td><td>3.47 (14.57)</td><td>2.5 % (8.6 %)</td></tr></table></td><td>Overall study quality:
High risk
Conflict of interest
Stated (none declared)
Funding:
Public funds

+ ethical approval from the University of Southern California Institutional Review Board
+ complete sampling of participants in the original study (all 9th grade students from 10 out of 40 invited public high schools which were chosen due to their diverse demographic characteristics and proximity)
+ high response
+ temporal investigation
+ consideration of the confounder “age” (homogeneous group)</td></tr>
</table>

<table>
<tr><th>Reference
(First author, publication year)</th><th>General
(Study design
Study name
Country of study
Time of study
No. of waves
Follow-up duration
(e.g., mean, range))</th><th>Population
(Description
Inclusion criteria
invited
baseline
follow-up
Age at baseline
% females
Response
Lost to follow-up)</th><th>Exposure
(Description & assessment)</th><th>Outcome
(Description & assessment)</th><th>Results
(Description of all topically relevant results)</th><th>Comments
(study quality [overall assessment according to Iljaz/Shamliyan/SIGN/CASP]
Conflict of interest [stated vs. not stated]
Funding (public, non-profit, commercial)
Strengths (+) and weaknesses (-))</th></tr>
<tr><td></td><td></td><td>From wave 1 (2013) to wave 10 (2020):
55.6 %
From wave 9 to wave 10: n.r.</td><td>(Measures were researched on the internet)</td><td></td><td></td><td>- self-reported outcome measurement
- no consideration of the confounder “sex” in statistical analysis
- descriptive analysis only</td></tr>
<tr><td>Niedzwiedz et al., 2020</td><td>Study design:
Cohort study
Study name:
UK Household Longitudinal Study (also called “Understanding Society)”
Country of study:
United Kingdom
Time of study:
2015–2020
Wave 7: 2015–2017
Wave 8: 2016–2018
Wave 9: 2017–2019
COVID-19 wave April 24–30, 2020
No. of waves:
n = 4
Follow-up duration:
Annually</td><td>Description:
Adults
Inclusion criteria:
Data from persons who took part in all 4 waves, having complete data sets at all 4 waves, aged ≥ 16 years
invited:
n.r.
# baseline:
n = 27,141 (wave 7)
# follow-up:
n = 25,231 (wave 8)
n = 22,823 (wave 9)
n = 10,977 (COVID-19 wave)
(analysed: n = 9,748 (repeated measures for all relevant waves)
Age at baseline:
18–24 years:
n = 3,009 (11.1 %)
25–44 years:
n = 8,504 (30.7 %)
45–64 years:
n = 9,766 (35.0 %)
≥ 65 years: n = 5,862 (23.2 %)
% females:
52.2 % (follow-up)</td><td>COVID-19 lockdown in the UK:
March 12, 2020:
- all with symptoms of possible COVID-19 required to isolate for 7 days
March 16, 2020:
- all living with someone with symptoms of possible COVID19 required to isolate for 14 days
- all others advised against unnecessary social contact and travel
- mass gatherings banned
March 17, 2020:
- foreign and Commonwealth Office advises against all non-essential world-wide travel
March 20, 2020:
- entertainment, hospitality and indoor leisure premises ordered to close
- schools, colleges and nurseries close for all except children of key workers or children identified as vulnerable by social services
March 22, 2020:
- Those identified as being extremely clinically vulnerable (i.e. those at highest risk of</td><td>Alcohol consumption:
Alcohol Use Disorder Identification Test for Consumption:
- binge drinking (≥ 6 drinks on a single occasion)
- alcohol frequency (≥ 4 times per week)
- heavy drinking (≥ 7 drinks on a typical drinking day)
Cigarette smoking:
2 self-reported single questions
- current smoker versus non-smoker
- number of cigarettes per day (< 10, 10–19, 20+ cigarettes)
E-Cigarette use:
1 single question (use of e-cigarettes once a week)</td><td>Table S2: Descriptive statistics (in %)
<table>
<tr><th></th><th>Wave 7</th><th>Wave 9</th><th>COVID-19 wave</th></tr>
<tr><td colspan="4">Binge drinking</td></tr>
<tr><td>No</td><td>88.5 (88.0–89.0)</td><td>89.2 (88.7–89.7)</td><td>83.8 (82.6–85.0)</td></tr>
<tr><td>Yes</td><td>11.5 (11.1–12.0)</td><td>10.8 (10.3–11.3)</td><td>16.2 (15.0–17.4)</td></tr>
<tr><td colspan="4">Number of drinks on typical drinking day</td></tr>
<tr><td>0–4</td><td>86.7 (86.1–87.2)</td><td>86.4 (85.7–87.0)</td><td>94.5 (93.6–95.2)</td></tr>
<tr><td>≥ 5</td><td>13.3 (12.8–13.9)</td><td>13.6 (13.0–14.3)</td><td>5.6 (4.8–6.4)</td></tr>
<tr><td colspan="4">Alcohol frequency (in times per week)</td></tr>
<tr><td>0–3</td><td>87.2 (86.6–87.7)</td><td>86.3 (85.7–86.9)]</td><td>78.0 (76.6–79.4)</td></tr>
<tr><td>≥ 4</td><td>12.8 (12.3–13.4)</td><td>13.7 (13.1–14.3)</td><td>22.0 (20.6–23.4)</td></tr>
</table>
Table S5: Binge drinking
<table>
<tr><th></th><th>Model 1
RR (95 % CI)</th><th>Model 2
RR (95 % CI)</th></tr>
<tr><td>During Covid-19</td><td>1.18 (0.97–1.45)</td><td>1.27 (1.08–1.48)</td></tr>
</table></td><td>Overall study quality:
Outcome “alcohol consumption”: Low risk
Outcome “smoking”: High risk
Conflict of interest
Stated (none declared)
Funding:
Public funds

+ ethical approval from University of Essex Ethics Committee
+ clustered stratified probability sample
+ large UK household survey
+ high response
+ validated outcome measurement for drinking behaviour
+ temporal investigation
+ regression analysis for the investigation of associations
+ statistical analysis of confounders “sex” and “age” (interaction analysis)

- high lost to follow-up (and no information on drop-outs)
- single questions for</td></tr>
</table>

**Reference**<br>(First author, publication year)	**General**<br>(Study design<br>Study name<br>Country of study<br>Time of study<br>No. of waves<br>Follow-up duration<br>(e.g., mean, range))	**Population**<br>(Description<br>Inclusion criteria<br># invited<br># baseline<br># follow-up<br>Age at baseline<br>% females<br>Response<br>Lost to follow-up)	**Exposure**<br>(Description & assessment)	**Outcome**<br>(Description & assessment)	**Results**<br>(Description of all topically relevant results)	**Comments**<br>(study quality [overall assessment according to Iljaz/Shamliyan/SIGN/CASP]<br>Conflict of interest [stated vs. not stated]<br>Funding (public, non-profit, commercial)<br>Strengths (+) and weaknesses (-))
		**Response:**<br>Household response waves 7–9: 81–84 %<br>COVID-19 wave (of those who took part in wave 9): 48.6 %<br>**Lost to follow-up:**<br>Wave 7 to COVID-19 wave: 59.6 %	severe illness if they contract COVID-19) advised to begin 'shielding' – they should not leave their house for any reason, necessary food/ supplies will be provided by Local Authorities where needed<br>**March 23, 2020:**<br>- hole population not permitted to leave home except for very limited purposes (to buy food; to exercise once per day; for any medical need; to care for a vulnerable person; to travel to/from essential work)<br>- all gatherings of more than two people in public banned<br>- lockdown measures to be reviewed every 3 weeks<br>**March 27, 2020:**<br>- public advised to only use open spaces near own house for exercise, and to stay at least 2metres apart from other households while outdoors<br>**April:**<br>- no significant change to lockdown measures		(see tables below)	

Results (continued):

**Age group interaction with period** (Pre-COVID-19 is ref, 65+ is ref)		
18–24	1.06 (0.77–1.46)	
25–44	1.64 (1.35–1.98)	
45–64	1.16 (0.98–1.37)	
**Gender interaction with period** (Pre-COVID-19 is ref, male is ref)		
Female		1.49 (1.31–1.69)

Table S6: Alcohol frequency (drinking 4+ days per week)

	**Model 1**<br>RR (95 % CI)	**Model 2**<br>RR (95 % CI)
During Covid-19	1.06 (0.96–1.17)	1.23 (1.11–1.35)
**Age group interaction with period** (Pre-COVID-19 is ref, 65+ is ref)		
18–24	2.90 (1.63–5.15)	
25–44	1.91 (1.66–2.20)	
45–64	1.25 (1.15–1.35)	
**Gender interaction with period** (Pre-COVID-19 is ref, male is ref)		
Female		1.34 (1.23–1.47)

Table S7: Heavy drinking (5+ drinks on a typical day when drinking)

	**Model 1**<br>RR (95 % CI)	**Model 2**<br>RR (95 % CI)
During Covid-19	0.60 (0.42–0.86)	0.46 (0.38–0.55)
**Age group interaction with period** (Pre-COVID-19 is ref, 65+ is ref)		
18–24	0.37 (0.22–0.63)	
25–44	0.68 (0.47–0.99)	
45–64	0.86 (0.60–1.23)	
**Gender interaction with period** (Pre-COVID-19 is ref, male is ref)		

<table>
<tr>
<th>Reference
(First au-
thor, publi-
cation year)</th>
<th>General
(Study design
Study name
Country of study
Time of study
No. of waves
Follow-up duration
(e.g., mean, range))</th>
<th>Population
(Description
Inclusion criteria
invited
baseline
follow-up
Age at baseline
% females
Response
Lost to follow-up)</th>
<th>Exposure
(Description & assessment)</th>
<th>Outcome
(Description & assess-
ment)</th>
<th>Results
(Description of all topically relevant results)</th>
<th>Comments
(study quality [overall as-
sessment according to
Iljaz/Shamliyan/SIGN/CASP]
Conflict of interest [stated
vs. not stated]
Funding (public, non-profit,
commercial)
Strengths (+) and weak-
nesses (-))</th>
</tr>
<tr>
<td></td>
<td></td>
<td></td>
<td></td>
<td></td>
<td>
<table>
<tr><td>Female</td><td></td><td>0.70 (0.56–0.88)</td></tr>
</table>

Table S8: Current smoking
<table>
<tr><td></td><td>Model 1
RR (95 % CI)</td><td>Model 2
RR (95 % CI)</td></tr>
<tr><td>During
Covid-19</td><td>0.80 (0.69–0.93)</td><td>0.88 (0.78–0.98)</td></tr>
<tr><td colspan="3">Age group interaction with period (Pre-COVID-19 is ref, 65+ is ref)</td></tr>
<tr><td>18–24</td><td>1.23 (0.95–1.60)</td><td></td></tr>
<tr><td>25–44</td><td>1.08 (0.91–1.28)</td><td></td></tr>
<tr><td>45–64</td><td>1.14 (0.99–1.32)</td><td></td></tr>
<tr><td colspan="3">Gender interaction with period (Pre-COVID-19 is ref, male is ref)</td></tr>
<tr><td>Female</td><td></td><td>1.03 (0.93–1.16)</td></tr>
</table>

Table S9: Regular e-cigarette use
<table>
<tr><td></td><td>Model 1
RR (95 % CI)</td><td>Model 2
RR (95 % CI)</td></tr>
<tr><td>During
Covid-19</td><td>0.68 (0.46–1.01)</td><td>0.61 (0.43–0.86)</td></tr>
<tr><td colspan="3">Age group interaction with period (Pre-COVID-19 is ref, 65+ is ref)</td></tr>
<tr><td>18–24</td><td>1.01 (0.40–2.58)</td><td></td></tr>
<tr><td>25–44</td><td>0.92 (0.64–1.31)</td><td></td></tr>
<tr><td>45–64</td><td>1.00 (0.71–1.39)</td><td></td></tr>
<tr><td colspan="3">Gender interaction with period (Pre-COVID-19 is ref, male is ref)</td></tr>
<tr><td>Female</td><td></td><td>1.20 (0.91–1.58)</td></tr>
</table>
Multi-level Poisson regression
Model 1: adjusted for year, age group, gender, ethnicity, period and period x age group interaction
Model 2: adjusted for year, age group, gender, ethnicity, period and period x gender interaction
</td>
<td></td>
</tr>
<tr>
<td>Okely et al., 2020</td>
<td>Study design:
Cohort study
Study name:</td>
<td>Description:
Community-dwelling older adults (born in 1936)</td>
<td>COVID-19 lockdown (that lasted 34 days at the beginning of data collection)</td>
<td>Physical activity:
Self-reported single question (“What level of physical activity do you</td>
<td>Table 1: Change in physical activity between 2017–2019 and COVID-19 lockdown in 2020</td>
<td>Overall study quality:
High risk
Conflict of interest
Stated (none declared)</td>
</tr>
</table>

<table>
<tr><th>Reference
(First author, publication year)</th><th>General
(Study design
Study name
Country of study
Time of study
No. of waves
Follow-up duration
(e.g., mean, range))</th><th>Population
(Description
Inclusion criteria
invited
baseline
follow-up
Age at baseline
% females
Response
Lost to follow-up)</th><th>Exposure
(Description & assessment)</th><th>Outcome
(Description & assessment)</th><th>Results
(Description of all topically relevant results)</th><th>Comments
(study quality [overall assessment according to Iljaz/Shamliyan/SIGN/CASP]
Conflict of interest [stated vs. not stated]
Funding (public, non-profit, commercial)
Strengths (+) and weaknesses (-))</th></tr>
<tr><td></td><td>Lothian Birth Cohort 1936 (LBC1936) study
Country of study:
Scotland
Time of study:
Baseline (T1): 2017–2019 (wave 5) of the LBC1936 study
Follow-up (T2): May 27–June 8, 2020
No. of waves:
n = 2
Follow-up duration:
~2 years</td><td>Inclusion criteria:
n.r.
# invited at follow-up:
n = 454
# baseline:
n.r.
# follow-up:
n = 137
Age at baseline:
M: 84 years
% females:
48.2 %
Response:
30.2 %
Lost to follow-up:
n.r.</td><td></td><td>mainly do?") with a 6-point scale (ranging from score 1: "moving only in connection with necessary (household) chores" to score 6 "keep fit/heavy exercise or competitive sport several times per week".)</td><td>
<table>
<tr><th></th><th>Baseline
(n (%))</th><th>Follow-up
(n (%))</th><th>p</th></tr>
<tr><td colspan="4">Physical activity (6-point scale)[1]</td></tr>
<tr><td>Only household chores</td><td>14
(10.2 %)</td><td>26
(19.0 %)</td><td rowspan="6">0.012</td></tr>
<tr><td>Outdoor activities 1–2x/ week</td><td>28
(20.4 %)</td><td>23
(16.8 %)</td></tr>
<tr><td>Outdoor activities > 2x/ week</td><td>67
(48.9 %)</td><td>74
(54.0 %)</td></tr>
<tr><td>Moderate exercise 1–2x/ week</td><td>19
(13.9 %)</td><td>4 (2.9 %)</td></tr>
<tr><td>Moderate exercise > 2x/ week</td><td>6 (4.4 %)</td><td>10 (7.3 %)</td></tr>
<tr><td>Keep-fit/heavy exercise several times/ week</td><td>3 (2.2 %)</td><td>0 (0.0 %)</td></tr>
<tr><td colspan="4">Physical activity (2-point scale)[2]</td></tr>
<tr><td>Minimal physical activity (score 1)</td><td>14
(10.2 %)</td><td>26
(19.0 %)</td><td rowspan="2">0.038</td></tr>
<tr><td>Any higher category of physical activity (scores 2–6)</td><td>123 (89.8)</td><td>111 (81.0)</td></tr>
</table>
[1]vage

[2]McNemar's test (with continuity correction)</td><td>Funding:
Public funds

+ ethical approval from the Multi-Centre Research Ethics Committee for Scotland, the Lothian Research Ethics Committee, and the Scotland A Research Ethics Committee
+ complete sampling (from all eligible participants of the LBC1936 study (further representative sampling of the original study)
+ using Wilcoxon signed rank test and McNemar's test to investigate differences
+ consideration of the confounder "age" (homogeneous group)
+ temporal investigation

- outcome measurement with a self-developed, self-reported single question
- no consideration of the confounder "sex" in statistical analysis
- some changes may have been caused by the effects of ageing rather than lockdown measures, however, results from subsidiary analysis, testing for changes in physical activity between earlier waves of the LBC1936 study, suggest</td></tr>
</table>

<table>
<tr><th>Reference
(First author, publication year)</th><th>General
(Study design
Study name
Country of study
Time of study
No. of waves
Follow-up duration
(e.g., mean, range))</th><th>Population
(Description
Inclusion criteria
invited
baseline
follow-up
Age at baseline
% females
Response
Lost to follow-up)</th><th>Exposure
(Description & assessment)</th><th>Outcome
(Description & assessment)</th><th>Results
(Description of all topically relevant results)</th><th>Comments
(study quality [overall assessment according to Iljaz/Shamliyan/SIGN/CASP]
Conflict of interest [stated vs. not stated]
Funding (public, non-profit, commercial)
Strengths (+) and weaknesses (-))</th></tr>
<tr><td></td><td></td><td></td><td></td><td></td><td></td><td>that age-related changes are typically smaller than those documented in the study
- drop-out analysis: difference between excluded and included participants: included individuals had a higher IQ at age 1, were better in cognition (cognitive ability, less severe memory problems), had more years of education, a higher occupational social class, a higher intellect, and a higher emotional stability</td></tr>
<tr><td>Radwan et al., 2021</td><td>Study design:
Cross-sectional study
Study name:
n.a.
Country of study:
United Arab Emirates
Time of study:
May 5–18, 2020
No. of waves:
n = 1
Follow-up duration:
n.a.</td><td>Description:
Adults
Inclusion criteria:
Residents of the United Arab Emirates, aged ≥ 18 years
# invited:
n = 13,500
# baseline:
n = 2,135
# included in analysis
n = 2,060
# follow-up:
n.a.
Age at baseline:
18–30 years: 31.7 %,
31–40 years: 38.4 %,
> 40 years: 29.9 %
% females:
75.1 %
Response:
15.8 %
Lost to follow-up:</td><td>COVID-19 lockdown:
- on March 22, 2020, the United Arab Emirates authorities applied both strict infection control and partial lockdown</td><td>(Change in) dietary intake:
Self-reported question
(Change in) weight:
Self-reported question
(Change in) physical activity:
Self-reported question
(Change in) smoking:
Self-reported question</td><td>Table 2: Descriptive statistics for changes in lifestyle behaviors
<table>
<tr><th></th><th>Prevalence (n (%)</th></tr>
<tr><td>Physical activity</td><td></td></tr>
<tr><td>Increase</td><td>340 (16.5)</td></tr>
<tr><td>Decrease</td><td>618 (30.0)</td></tr>
<tr><td>Same</td><td>1,102 (53.5)</td></tr>
<tr><td>Food intake</td><td></td></tr>
<tr><td>Increase</td><td>655 (31.8)</td></tr>
<tr><td>Decrease</td><td>344 (16.7)</td></tr>
<tr><td>Same</td><td>1,061 (51.5)</td></tr>
<tr><td>Weight</td><td></td></tr>
<tr><td>Increase</td><td>606 (29.4)</td></tr>
<tr><td>Decrease</td><td>476 (23.1)</td></tr>
<tr><td>Same</td><td>978 (47.5)</td></tr>
<tr><td>Smoking</td><td></td></tr>
<tr><td>Increase</td><td>50 (21.0)</td></tr>
<tr><td>Decrease</td><td>93 (39.1)</td></tr>
<tr><td>Same</td><td>95 (39.9)</td></tr>
</table>
Tables A1–A3, and A4: Logistic regressions describing the association between unhealthy lifestyle changes due to lockdown and sex/age</td><td>Overall study quality:
High risk
Conflict of interest
Stated (none declared)
Funding:
None

+ ethical approval from the Research and Ethics Committee at the University of Sharjah
+ questionnaire link was distributed to 13,500 adults via emails and mobile messages (list of email addresses and phone numbers was obtained from the database of the Supreme Council for Family Affairs, Health promotion Department, Sharjah and their network in the various Emirates of the United Arab Emirates)</td></tr>
</table>

<table>
<tr><th>Reference
(First author, publication year)</th><th>General
(Study design
Study name
Country of study
Time of study
No. of waves
Follow-up duration
(e.g., mean, range))</th><th>Population
(Description
Inclusion criteria
invited
baseline
follow-up
Age at baseline
% females
Response
Lost to follow-up)</th><th>Exposure
(Description & assessment)</th><th>Outcome
(Description & assessment)</th><th>Results
(Description of all topically relevant results)</th><th>Comments
(study quality [overall assessment according to Iljaz/Shamliyan/SIGN/CASP]
Conflict of interest [stated vs. not stated]
Funding (public, non-profit, commercial)
Strengths (+) and weaknesses (-))</th></tr>
<tr><td></td><td></td><td>n.a.</td><td></td><td></td><td><table>
<tr><th></th><th></th><th>OR (95 % CI)</th></tr>
<tr><td colspan="3">Increased food intake</td></tr>
<tr><td>Sex</td><td>Males</td><td>Reference</td></tr>
<tr><td></td><td>Females</td><td>1.89 (1.47–2.41)</td></tr>
<tr><td>Age</td><td>18–30 years</td><td>Reference</td></tr>
<tr><td></td><td>31–40 years</td><td>0.69 (0.53–0.89)</td></tr>
<tr><td></td><td>> 40 years</td><td>0.55 (0.41–0.73)</td></tr>
<tr><td colspan="3">Increased weight</td></tr>
<tr><td>Sex</td><td>Males</td><td>Reference</td></tr>
<tr><td></td><td>Females</td><td>1.89 (1.47–2.41)</td></tr>
<tr><td>Age</td><td>18–30 years</td><td>Reference</td></tr>
<tr><td></td><td>31–40 years</td><td>0.69 (0.53–0.89)</td></tr>
<tr><td></td><td>> 40 years</td><td>0.55 (0.41–0.73)</td></tr>
<tr><td colspan="3">Decreased physical activity</td></tr>
<tr><td>Sex</td><td>Males</td><td>Reference</td></tr>
<tr><td></td><td>Females</td><td>1.12 (0.88–1.43)</td></tr>
<tr><td>Age</td><td>18–30 years</td><td>Reference</td></tr>
<tr><td></td><td>31–40 years</td><td>1.24 (0.98–1.59)</td></tr>
<tr><td></td><td>> 40 years</td><td>1.61 (1.25–2.08)</td></tr>
<tr><td colspan="3">Increased smoking</td></tr>
<tr><td>Sex</td><td>Males</td><td>Reference</td></tr>
<tr><td></td><td>Females</td><td>0.27 (0.15–0.49)</td></tr>
<tr><td>Age</td><td>18–30 years</td><td>Reference</td></tr>
<tr><td></td><td>31–40 years</td><td>0.60 (0.31–1.15)</td></tr>
<tr><td></td><td>> 40 years</td><td>0.25 (0.11–0.57)</td></tr>
</table>
Adjusted for sex, age, nationality, type of living, number of children, BMI</td><td>+ statistical analysis of the confounders “age” and “sex

- low response
- outcome measurement with self-reported single questions
- self-developed questionnaire (test prior with a convenience sample)
- descriptive analysis of main outcomes
- due to study design no actual chronological investigation</td></tr>
<tr><td>Sasaki et al., 2021</td><td>Study design:
Cross-sectional study
Study name:
n.a.
Country of study:
Japan
Time of study:
August 2020
No. of waves:</td><td>Description:
Older adults (60–95 years)
Inclusion criteria:
n.r.
# invited:
n = 2,008
# baseline:
n =1,493
n = 999 (full data set)</td><td>COVID-19-related distancing restrictions</td><td>(Change in) physical activity:
International Physical Activity Questionnaire Short Form (IPAQ-SF)

Classification:
- low physical activity (LPA)</td><td>Table 2: Descriptive statistics for the change in physical activity since lockdown
<table>
<tr><th></th><th>Prevalence (%)</th></tr>
<tr><td colspan="2">Men</td></tr>
<tr><td>Maintained low physical activity</td><td>34.4</td></tr>
<tr><td>Decreased</td><td>4.6</td></tr>
<tr><td>Maintained moderate-to-vigorous physical activity</td><td>59.1</td></tr>
<tr><td>Increased</td><td>1.9</td></tr>
<tr><td colspan="2">Women</td></tr>
</table></td><td>Overall study quality:
High risk
Conflict of interest
Stated (none declared)
Funding:
None

+ ethical approval from the institutional ethics board for epidemiological studies of</td></tr>
</table>

<table>
<tr>
<th>Reference
(First author, publication year)</th>
<th>General
(Study design
Study name
Country of study
Time of study
No. of waves
Follow-up duration
(e.g., mean, range))</th>
<th>Population
(Description
Inclusion criteria
invited
baseline
follow-up
Age at baseline
% females
Response
Lost to follow-up)</th>
<th>Exposure
(Description & assessment)</th>
<th>Outcome
(Description & assessment)</th>
<th>Results
(Description of all topically relevant results)</th>
<th>Comments
(study quality [overall assessment according to Iljaz/Shamliyan/SIGN/CASP]
Conflict of interest [stated vs. not stated]
Funding (public, non-profit, commercial)
Strengths (+) and weaknesses (-))</th>
</tr>
<tr>
<td></td>
<td>n = 1
Follow-up duration:
n.a.</td>
<td># follow-up:
n.a.
Age at baseline:
M: 74.5 years (SD: 6.3)
% females:
53.8 %
Response:
74.3% (full data set: 49.8 %)
Lost to follow-up:
n.a.</td>
<td></td>
<td>- moderate-to-vigorous physical activity (MVPA)
- walking: 3.3 METs
- moderate-intensity activity: 4.0 METs
- vigorous activity: 8.0 METs

Sitting:
International Physical Activity Questionnaire Short Form (minutes/day)</td>
<td>
<table>
<tr><td>Maintained low physical activity</td><td>47.5</td></tr>
<tr><td>Decreased</td><td>6.0</td></tr>
<tr><td>Maintained moderate-to-vigorous physical activity</td><td>45.2</td></tr>
<tr><td>Increased</td><td>1.3</td></tr>
</table>
Table 2: Differences in physical activity before and after the onset of COVID-19 restrictions
<table>
<tr><th></th><th>Before restrictions (M (SD))</th><th>After restrictions (M (SD))</th><th>p</th></tr>
<tr><td colspan="4">Men</td></tr>
<tr><td>Vigorous intensity (MET)</td><td>1690.6 (2668.8)</td><td>1604.8 (2598.2)</td><td>0.035</td></tr>
<tr><td>Moderate intensity (MET)</td><td>1064.7 (1332.8)</td><td>1002.6 (1306.4)</td><td>0.0024</td></tr>
<tr><td>Walking (MET)</td><td>922.9 (1035.5)</td><td>877.4 (1028.9)</td><td>0.0054</td></tr>
<tr><td>Total physical activity (MET)</td><td>3678.2 (4163.1)</td><td>3484.8 (4112.3)</td><td>0.0024</td></tr>
<tr><td>Sitting time (minutes/ day)</td><td>273.4 (203.4)</td><td>287.7 (204.1)</td><td>< 0.001</td></tr>
<tr><td colspan="4">Women</td></tr>
<tr><td>Vigorous intensity (MET)</td><td>742.5 (1701.3)</td><td>717.5 (1738.0)</td><td>0.40</td></tr>
<tr><td>Moderate intensity (MET)</td><td>712.5 (1062.7)</td><td>644.4 (1005.1)</td><td>0.0022</td></tr>
<tr><td>Walking (MET)</td><td>717.2 (899.6)</td><td>647.2 (870.5)</td><td>< 0.001</td></tr>
</table>
</td>
<td>the Hokkaido Bunkyo University
+ stratified sampling method (random selection in terms of sex, age, residence, and social participation), based on the list held by Eniwa City Hall
+ high response
+ validated outcome measurement
+ using paired t-test or McNemar’s test to investigate differences
+ statistical analysis of the confounder “sex” (by stratification)

- no consideration of the confounder “age” in the statistical analysis
- due to study design no actual chronological investigation</td>
</tr>
</table>

<table>
<tr><th>Reference
(First author, publication year)</th><th>General
(Study design
Study name
Country of study
Time of study
No. of waves
Follow-up duration
(e.g., mean, range))</th><th>Population
(Description
Inclusion criteria
invited
baseline
follow-up
Age at baseline
% females
Response
Lost to follow-up)</th><th>Exposure
(Description & assessment)</th><th>Outcome
(Description & assessment)</th><th>Results
(Description of all topically relevant results)</th><th>Comments
(study quality [overall assessment according to Iljaz/Shamliyan/SIGN/CASP]
Conflict of interest [stated vs. not stated]
Funding (public, non-profit, commercial)
Strengths (+) and weaknesses (-))</th></tr>
<tr><td></td><td></td><td></td><td></td><td></td><td><table>
<tr><td>Total physical activity (MET)</td><td>2172.1 (2873.2)</td><td>2009.2 (2876.6)</td><td>< 0.001</td></tr>
<tr><td>Sitting time (minutes/ day)</td><td>243.7 (181.5)</td><td>267.8 (191.6)</td><td>< 0.001</td></tr>
</table>Paired t-test or McNemar's test</td><td></td></tr>
<tr><td colspan="7">CHILDREN AND ADOLESCENTS</td></tr>
<tr><td>McCormack et al., 2020</td><td>Study design:
Cross-sectional study
Study name:
n.a.
Country of study:
Canada
Time of study:
April 14–May 27, 2020
No. of waves:
n = 1
Follow-up duration:
n.r.</td><td>Description:
Children aged 5–17 years
Inclusion criteria:
n.r.
# invited:
n = 25,000
# baseline:
n = 1,124 (adults)
n = 345 (parents with at least one school-aged child)
n = 328 (children)
# follow-up:
n.a.
Age at baseline:
5–11 years: 80.5 %,
12–17 years: 19.5 %
% females:
45.1 %
Response:
4.5 % (adults)
1.3 % (parents with complete data)
Lost to follow-up:
n.a.</td><td>COVID-19 public health emergency response:
- forced closures of educational and day-care facilities, non-essential businesses, and private and public recreation facilities (e.g., playgrounds, outdoor exercise equipment, and sports courts and fields)
- individuals and families were advised to take personal steps to reduce virus transmission including practicing physical distancing (i.e., avoiding crowded places, avoiding common greetings, limiting contact with High risk individuals, and maintaining a distance of 2-metres from others), forgoing international travel, and self-quarantining if they experienced symptoms</td><td>(Change in) physical activity:
Parents-reported questions
(Change in) sedentary behaviours:
Parents-reported questions</td><td>Table 2: Descriptive statistics for the change physical activity and sedentary behaviors since COVID-19 public health emergency response<table>
<tr><th></th><th>Prevalence (%)</th></tr>
<tr><td colspan="2">Physically active in home</td></tr>
<tr><td>Increased</td><td>48.8</td></tr>
<tr><td>No change</td><td>32.9</td></tr>
<tr><td>Decreased</td><td>18.3</td></tr>
<tr><td colspan="2">Physically active outdoors</td></tr>
<tr><td>Increased</td><td>38.7</td></tr>
<tr><td>No change</td><td>22.3</td></tr>
<tr><td>Decreased</td><td>39.0</td></tr>
<tr><td colspan="2">Watching television</td></tr>
<tr><td>Increased</td><td>58.8</td></tr>
<tr><td>No change</td><td>38.4</td></tr>
<tr><td>Decreased</td><td>2.7</td></tr>
<tr><td colspan="2">Playing video games</td></tr>
<tr><td>Increased</td><td>56.4</td></tr>
<tr><td>No change</td><td>40.9</td></tr>
<tr><td>Decreased</td><td>2.7</td></tr>
<tr><td colspan="2">Using screen-based devices</td></tr>
<tr><td>Increased</td><td>75.9</td></tr>
<tr><td>No change</td><td>22.0</td></tr>
<tr><td>Decreased</td><td>2.1</td></tr>
<tr><td colspan="2">Playing at park</td></tr>
<tr><td>Increased</td><td>15.5</td></tr>
<tr><td>No change</td><td>31.7</td></tr>
</table></td><td>Overall study quality:
High risk
Conflict of interest
Stated (none declared)
Funding:
Public and non-profit funds

+ ethical approval from the University of Calgary Conjoint Human Research Ethics Board
+ simple random sample of 25,000 households with equal chance of selection across 14 adjacent communities, one adult per household
+ consideration of the confounder "age" (homogeneous group)

- low response
- parental-reported questions
- descriptive analysis only
- no consideration of the confounder "sex" in the statistical analysis</td></tr>
</table>

<table>
<tr>
<th>Reference
(First author, publication year)</th>
<th>General
(Study design
Study name
Country of study
Time of study
No. of waves
Follow-up duration
(e.g., mean, range))</th>
<th>Population
(Description
Inclusion criteria
invited
baseline
follow-up
Age at baseline
% females
Response
Lost to follow-up)</th>
<th>Exposure
(Description & assessment)</th>
<th>Outcome
(Description & assessment)</th>
<th>Results
(Description of all topically relevant results)</th>
<th>Comments
(study quality [overall assessment according to Iljaz/Shamliyan/SIGN/CASP]
Conflict of interest [stated vs. not stated]
Funding (public, non-profit, commercial)
Strengths (+) and weaknesses (-))</th>
</tr>
<tr>
<td></td>
<td></td>
<td></td>
<td></td>
<td></td>
<td><table>
<tr><td>Decreased</td><td>52.7</td></tr>
<tr><td colspan="2">Playing at other public places</td></tr>
<tr><td>Increased</td><td>9.5</td></tr>
<tr><td>No change</td><td>36.9</td></tr>
<tr><td>Decreased</td><td>53.7</td></tr>
</table></td>
<td>- due to study design no actual chronological investigation</td>
</tr>
<tr>
<td>Medrano et al., 2020</td>
<td>Study design:
Cohort study
Study name:
MUGI project
Country of study:
Spain
Time of study:
T1: September–December 2019
T2: March–April 2020
No. of waves:
n = 2
Follow-up duration:
Around 4–8 month</td>
<td>Description:
School children (pupils), aged 8–16 years
Inclusion criteria:
Exclusion of pupils with an intellectual or a physical disability
# invited:
n = 348
# baseline:
n = 291
# follow-up:
n = 113
Age at baseline:
M: 12.1 years (SD: 2.4)
% females:
47.8 %
Response:
83.6%
Lost to follow-up:
61.2 %</td>
<td>Home confinement during the COVID-19 pandemic:
- closuring schools and establishing mandatory home confinement for children (6 weeks of total locked down, where children were not allowed to leave their house at all, from March 14 to April 26, 2020)</td>
<td>Physical activity:
“The Youth Activity Profile” questionnaire (YAP), measured in minutes/day
Screen time:
“The Youth Activity Profile” questionnaire (YAP)
Adherence to Mediterranean diet:
Mediterranean Diet Quality Index for children and teenagers (KIDMED) questionnaire (low adherence: score <8/12)</td>
<td>Table 2: Changes in lifestyle habits before and during the COVID-19 confinement<table>
<tr><th></th><th>T1
(M SD))</th><th>T2
(M SD))</th><th>p</th></tr>
<tr><td colspan="4">Physical activity</td></tr>
<tr><td>Physical activity (minutes/day)</td><td>154 (40)</td><td>63 (39)</td><td>< 0.001</td></tr>
<tr><td colspan="4">Screen time</td></tr>
<tr><td>Screen time (hours/day)</td><td>4.3 (2.4)</td><td>6.1 (2.4)</td><td>< 0.001</td></tr>
<tr><td>TV time ≥2 hours/day (N, %)</td><td>3 (2.8)</td><td>14 (13.2)</td><td>0.005</td></tr>
<tr><td>Videogame time ≥2 hours/day (N, %)</td><td>6 (5.7)</td><td>7 (6.6)</td><td>0.775</td></tr>
<tr><td>Computer (no homework) ≥2 hours/day (N, %)</td><td>1 (0.9)</td><td>0 (0.0)</td><td>0.316</td></tr>
<tr><td>Total mobile-phone ≥2 hours/day (N, %)</td><td>4 (3.8)</td><td>20 (18.9)</td><td>0.001</td></tr>
<tr><td>Total screen time ≥2 ≥2 hours/day (N, %)</td><td>70 (66.0)</td><td>93 (87.7)</td><td>< 0.001</td></tr>
<tr><td colspan="4">Adherence to Mediterranean diet</td></tr>
<tr><td>KIDMED score</td><td>5.9 (1.8)</td><td>6.4 (1.5)</td><td>0.018</td></tr>
<tr><td>Low adherence (KIDMED score: < 8)</td><td>86 (81.1)</td><td>81 (76.4)</td><td>0.476</td></tr>
</table></td>
<td>Overall study quality:
Low risk
Conflict of interest
Stated (none declared)
Funding:
Public funds

+ ethical approval from the Ethics Committee of the Public University of Navarra
+ informed consent by parents or legal guardians
+ multiple-stage process (random selection of type of school (primary and secondary), region and funding type (public, state funded, private) of school, school-year, classroom)
+ outcome measurement with validated questionnaires filled out by pupils
+ using t test and Chi² test to investigate differences
+ statistical analysis of confounders “sex” and “age” (homogeneous group)
+ temporal investigation

- high lost to follow-up, but drop-out analysis (difference regarding physical activity between responders at T2</td>
</tr>
</table>

<table>
<tr>
<th>Reference
(First au-
thor, publi-
cation year)</th>
<th>General
(Study design
Study name
Country of study
Time of study
No. of waves
Follow-up duration
(e.g., mean, range))</th>
<th>Population
(Description
Inclusion criteria
invited
baseline
follow-up
Age at baseline
% females
Response
Lost to follow-up)</th>
<th>Exposure
(Description & assessment)</th>
<th>Outcome
(Description & assess-
ment)</th>
<th>Results
(Description of all topically relevant results)</th>
<th>Comments
(study quality [overall as-
sessment according to
Iljaz/Shamliyan/SIGN/CASP]
Conflict of interest [stated
vs. not stated]
Funding (public, non-profit,
commercial)
Strengths (+) and weak-
nesses (-))</th>
</tr>
<tr>
<td></td>
<td></td>
<td></td>
<td></td>
<td></td>
<td>t test for continues variables and Chi^2 test for categorical variables

Text: Prevalence of worsening of lifestyle behaviours
<table>
<tr><th>Worsening of …</th><th>Prevalence (%)</th></tr>
<tr><td>… physical activity</td><td>95.2</td></tr>
<tr><td>… screen time</td><td>69.8</td></tr>
<tr><td>… adherence to the Mediterranean diet</td><td>31.4</td></tr>
</table>

Figures 1 and 2: Changes of lifestyle behaviours according to sex, school grade, and economic status
<table>
<tr><th>Differences in …</th><th>p</th></tr>
<tr><td colspan="2">… sex (boys vs. girls)</td></tr>
<tr><td>Physical activity</td><td>0.255</td></tr>
<tr><td>Screen time</td><td>0.020 (boys > girls)</td></tr>
<tr><td>Adherence to the Mediterranean diet</td><td>0.925</td></tr>
<tr><td colspan="2">… school grade (primary vs. secondary school)</td></tr>
<tr><td>Physical activity</td><td>0.795</td></tr>
<tr><td>Screen time</td><td>0.694</td></tr>
<tr><td>Adherence to the Mediterranean diet</td><td>0.916</td></tr>
<tr><td colspan="2">… economic status (low/medium vs. high)</td></tr>
<tr><td>Physical activity</td><td>0.152</td></tr>
<tr><td>Screen time</td><td>0.825</td></tr>
<tr><td>Adherence to the Mediterranean diet</td><td>0.598</td></tr>
</table>
</td>
<td>and drop-outs, but not regarding screen time and adherence to Mediterranean diet)</td>
</tr>
<tr>
<td>Ozturk Eyimaya & Yalçin Irmak, 2020</td>
<td>Study design:
Cross-sectional study
Study name:
n.a.
Country of study:
Turkey
Time of study:
May 15–31, 2020
No. of waves:</td>
<td>Description:
Children aged 6–13 years studying in 3 state schools (grades 1–8)
Inclusion criteria:
n.r.
# invited:
n = 1,544
# baseline:</td>
<td>Lockdown:
- closure of schools on March 16, 2020
- temporary lockdown on children and young people under the age of 20 on April 3, 2020</td>
<td>(Change in) screen time:
Single question (reported by the parents)</td>
<td>Text: Descriptive statistics for the change in screen time since lockdown (ignoring the time the children spent on academic studies)
<table>
<tr><th></th><th>Prevalence (%)</th></tr>
<tr><td>Increase</td><td>71.7</td></tr>
<tr><td>Decrease</td><td>6.1</td></tr>
<tr><td>No change</td><td>23.2</td></tr>
</table>
</td>
<td>Overall study quality:
High risk
Conflict of interest:
Stated (none declared)
Funding:
Unclear

+ ethical approval from the Namik Kemal University Faculty of Medicine Non-</td>
</tr>
</table>

<table>
<tr>
<th>Reference
(First author, publication year)</th>
<th>General
(Study design
Study name
Country of study
Time of study
No. of waves
Follow-up duration
(e.g., mean, range))</th>
<th>Population
(Description
Inclusion criteria
invited
baseline
follow-up
Age at baseline
% females
Response
Lost to follow-up)</th>
<th>Exposure
(Description & assessment)</th>
<th>Outcome
(Description & assessment)</th>
<th>Results
(Description of all topically relevant results)</th>
<th>Comments
(study quality [overall assessment according to Iljaz/Shamliyan/SIGN/CASP]
Conflict of interest [stated vs. not stated]
Funding (public, non-profit, commercial)
Strengths (+) and weaknesses (-))</th>
</tr>
<tr>
<td></td>
<td>n = 1
Follow-up duration:
n.a.</td>
<td>n = 1,115
follow-up:
n.a.
Age at baseline:
M: 9.03 years (SD: 1.95, range: 6–9)
% females:
53.4 %
Response:
72.2 % (parent response)
Lost to follow-up:
n.a.</td>
<td></td>
<td></td>
<td></td>
<td>invasive Clinical Trials Ethics Committee
+ random selection of schools, complete sampling on pupil’s level
+ high response
+ consideration of the confounder “age” (homogeneous group)

- parent-reported outcome measurement with a single item
- descriptive analysis only
- no consideration of the confounder “sex” in the statistical analysis
- due to study design no actual chronological investigation</td>
</tr>
<tr>
<td>Schmidt et al., 2020</td>
<td>Study design:
Cohort study
Study name:
German Motorik-Modul (MoMo) cohort study
Country of study:
Germany
Time of study:
Baseline (wave 3): August 2018
Follow-up: April 20–May 1, 2020
No. of waves:
n = 2
Follow-up duration:
Around 20 months</td>
<td>Description:
Children and adolescents
Inclusion criteria:
n.r.
# invited:
n = 10,802
# baseline:
n = 2,722
# follow-up:
n = 1,711
Age at baseline:
M: 10.36 years (SD: 4.04, range: 4–17)
% females:
49.8 %
Response:
25.2 %
Lost to follow-up:</td>
<td>COVID-19 lockdown:
- on March 11, 2020, the federal states in Germany closed kindergartens, schools, sports clubs, gyms, and other leisure institutions relevant to children’s and adolescents organized physical activity
- government also imposed physical distancing measures and contact restrictions, allowing no more than two people from different households to meet in public space
- nonorganized sports activities, such as workouts at home, or jogging, and other forms of habitual physical activity besides sports, like going</td>
<td>Physical activity:
MoMo PA Questionnaire (28 items)
Screen time:
Self-reported screen-time behaviours (time spent watching television (TV), playing games on any device (Gaming), and using the internet for recreational use (Internet) separately for weekdays and weekends, index reflecting weekdays and weekend days at a 5:2 ratio was then calculated for each activity)</td>
<td>Table 1: Days active (days/week) for more than 60 min with moderate to vigorous intensity
<table>
<tr><th></th><th>Baseline (M (SD))</th><th>Follow-up (M (SD))</th><th>Difference (95 % CI, d)</th></tr>
<tr><td>Total sample</td><td>4.3 (1.8)</td><td>4.7 (2.0)</td><td>+0.4 (0.4–0.5), 0.22 (p < 0.01)</td></tr>
<tr><td>Boys</td><td>4.4 (1.8)</td><td>4.8 (2.0)</td><td>+0.4 (0.3–0.6), 0.20 (p < 0.01)</td></tr>
<tr><td>Girls</td><td>4.1 (1.8)</td><td>4.6 (1.9)</td><td>+0.5 (0.3–0.6), 0.24 (p < 0.01)</td></tr>
</table>
Table 1: Physical activity guideline adherence
<table>
<tr><th></th><th>Baseline (%)</th><th>Follow-up (%)</th><th>Difference (95 % CI)</th></tr>
<tr><td>Total sample</td><td>19.1</td><td>30.1</td><td>+11.1 (8.2–13.9), (p < 0.01)</td></tr>
</table></td>
<td>Overall study quality:
High risk
Conflict of interest:
Stated (none declared)
Funding:
Public funds

+ ethical approval from the the Charité Universitätsmedizin Berlin ethics committee, by the University of Konstanz, and the ethics committee of the Karlsruhe Institute of Technology
+ informed consent by parents
+ nationwide multi-stage sampling approach with two evaluation levels: 1.)</td>
</tr>
</table>

<table>
<tr>
<th>Reference
(First author, publication year)</th>
<th>General
(Study design
Study name
Country of study
Time of study
No. of waves
Follow-up duration
(e.g., mean, range))</th>
<th>Population
(Description
Inclusion criteria
invited
baseline
follow-up
Age at baseline
% females
Response
Lost to follow-up)</th>
<th>Exposure
(Description & assessment)</th>
<th>Outcome
(Description & assessment)</th>
<th>Results
(Description of all topically relevant results)</th>
<th>Comments
(study quality [overall assessment according to Iljaz/Shamliyan/SIGN/CASP]
Conflict of interest [stated vs. not stated]
Funding (public, non-profit, commercial)
Strengths (+) and weaknesses (-))</th>
</tr>
<tr>
<td></td>
<td></td>
<td>36.4 %</td>
<td>for a walk or playing outside remained allowed if done alone or with people from the same household</td>
<td></td>
<td>
<table>
<tr><td>Boys</td><td>21.4</td><td>34.0</td><td>+12.5 (8.2–16.6),
(p < 0.01)</td></tr>
<tr><td>Girls</td><td>16.4</td><td>26.3</td><td>+9.5 (4.5–13.3),
(p < 0.01)</td></tr>
</table>
Table 2: Total amount of (organized and non-organized) sports (minutes per day)
<table>
<tr><th></th><th>Baseline
(M (SD))</th><th>Follow-up
(M (SD))</th><th>Difference
(95 % CI, d)</th></tr>
<tr><td>Total sample</td><td>34.9 (26.0)</td><td>24.3 (36.2)</td><td>-10.8 (-12.6–(-9.0)), 0.29
(p < 0.01)</td></tr>
<tr><td>Boys</td><td>36.3 (26.8)</td><td>24.6 (38.8)</td><td>-11.8 (-14.5–(-9.1)), 0.30
(p < 0.01)</td></tr>
<tr><td>Girls</td><td>33.5 (25.1)</td><td>24.0 (33.4)</td><td>-9.7 (-12.1–(-7.3)), 0.28
(p < 0.01)</td></tr>
</table>
Table 3: Total amount of habitual activity (playing outside, walking and cycling, gardening, housework) (minutes per day)
<table>
<tr><th></th><th>Baseline
(M (SD))</th><th>Follow-up
(M (SD))</th><th>Difference
(95 % CI, d)</th></tr>
<tr><td>Total sample</td><td>107.3 (73.9)</td><td>143.5 (110.5)</td><td>+36.2 (31.3–41.2), 0.35
(p < 0.01)</td></tr>
<tr><td>Boys</td><td>114.6 (75.5)</td><td>149.8 (115.5)</td><td>+35.2 (27.9–42.6), 0.32
(p < 0.01)</td></tr>
<tr><td>Girls</td><td>99.8 (71.4)</td><td>137.0 (104.8)</td><td>+37.2 (30.6–42.8), 0.38
(p < 0.01)</td></tr>
</table>
Table 1: Screen time guideline adherence
</td>
<td>German communities, 2.) age-stratified sample of randomly selected children and adolescents
+ validated outcome measurement
+ statistical analysis of confounders “sex” (by stratification) and “age” (homogeneous group)
+ using ANOVA to investigate differences
+ temporal investigation

- low response and high lost to follow-up</td>
</tr>
</table>

<table>
<tr>
<th>Reference
(First author, publication year)</th>
<th>General
(Study design
Study name
Country of study
Time of study
No. of waves
Follow-up duration
(e.g., mean, range))</th>
<th>Population
(Description
Inclusion criteria
invited
baseline
follow-up
Age at baseline
% females
Response
Lost to follow-up)</th>
<th>Exposure
(Description & assessment)</th>
<th>Outcome
(Description & assessment)</th>
<th>Results
(Description of all topically relevant results)</th>
<th>Comments
(study quality [overall assessment according to Iljaz/Shamliyan/SIGN/CASP]
Conflict of interest [stated vs. not stated]
Funding (public, non-profit, commercial)
Strengths (+) and weaknesses (-))</th>
</tr>
<tr>
<td></td>
<td></td>
<td></td>
<td></td>
<td></td>
<td>
<table>
<tr><th></th><th>Baseline (%)</th><th>Follow-up (%)</th><th>Difference (95 % CI)</th></tr>
<tr><td>Total sample</td><td>60.9</td><td>37.6</td><td>-23.3 (-20.0–(-26.5)),
p < 0.01)</td></tr>
<tr><td>Boys</td><td>60.7</td><td>35.2</td><td>-25.5 (-20.8–(-30.0)),
(p < 0.01)</td></tr>
<tr><td>Girls</td><td>61.1</td><td>40.0</td><td>-21.1 (-16.4–(-25.7),
(p < 0.01)</td></tr>
</table>
Table 4: Total amount of recreational screen time (TV, gaming, recreational internet) (minutes per day)
<table>
<tr><th></th><th>Baseline (M (SD))</th><th>Follow-up (M (SD))</th><th>Difference (95 % CI, d)</th></tr>
<tr><td>Total sample</td><td>133.3 (123.1)</td><td>194.5 (141.3)</td><td>+61.2 (56.2–66.3), 0.58 (p < 0.01)</td></tr>
<tr><td>Boys</td><td>139.2 (130.4)</td><td>205.4 (146.8)</td><td>+66.2 (58.6–73.7), 0.59 (p < 0.01)</td></tr>
<tr><td>Girls</td><td>127.3 (115.0)</td><td>183.6 (134.6)</td><td>+56.3 (9.6–63.0), 0.57 (p < 0.01)</td></tr>
</table>
ANOVA for repeated measurements
</td>
<td></td>
</tr>
<tr>
<td>Tornaghi et al., 2020</td>
<td>Study design:
Cohort study
Study name:
n.a.
Country of study:
Italy
Time of study:
T1: January 27–30, 2020 (pre-lockdown)
T2: April 4–10, 2020 (during lockdown)</td>
<td>Description:
Adolescents (15–18 years)
Inclusion criteria:
n.r.
# invited:
n = 1,686 (personal communication
# baseline:
n = 1,568
# follow-up:
n = 1,568</td>
<td>COVID-19 lockdown (2 Decrees of the Italian government on March 11 and 22, 2020):
- abrogation of nonessential movement, including outdoor sports and motor activity, with the exception of activities practiced a 200 meters home-block area and provision of at least 1 meter of interhuman distance</td>
<td>Physical activity:
International Physical Activity Questionnaire:
- inactive:
< 700 minutes/week
- moderate: 700–2519 minutes/week
- intense:
≥ 2520 minutes/week</td>
<td>
Table 1: Prevalence of physical activity levels
<table>
<tr><th></th><th>Pre-lockdown (n (%))</th><th>During lockdown (n (%))</th><th>Post-lockdown (n (%))</th></tr>
<tr><td>Physically inactive</td><td>154 (17.8)</td><td>102 (25.8)</td><td>53 (18.5)</td></tr>
<tr><td>Moderate activity</td><td>573 (66.3)</td><td>214 (53.6)</td><td>177 (61.7)</td></tr>
<tr><td>Intense activity</td><td>137 (15.8)</td><td>79 (19.8)</td><td>57 (19.9)</td></tr>
</table>
Figure 1: Continuous levels of physical activity
</td>
<td>Overall study quality:
High risk
Conflict of interest:
Stated (none declared)
Funding:
None

+ ethical approval from the Department of Psychology of Development and Socialization Process Ethical</td>
</tr>
</table>

<table>
<tr>
<th>Reference
(First author, publication year)</th>
<th>General
(Study design
Study name
Country of study
Time of study
No. of waves
Follow-up duration
(e.g., mean, range))</th>
<th>Population
(Description
Inclusion criteria
invited
baseline
follow-up
Age at baseline
% females
Response
Lost to follow-up)</th>
<th>Exposure
(Description & assessment)</th>
<th>Outcome
(Description & assessment)</th>
<th>Results
(Description of all topically relevant results)</th>
<th>Comments
(study quality [overall assessment according to Iljaz/Shamliyan/SIGN/CASP]
Conflict of interest [stated vs. not stated]
Funding (public, non-profit, commercial)
Strengths (+) and weaknesses (-))</th>
</tr>
<tr>
<td></td>
<td>T3: May 4–10, 2020 (post lockdown)
No. of waves:
n = 3
Follow-up duration:
Around 4 months</td>
<td>Age at baseline:
n.r.
% females:
n.r.
Response:
93 % (personal communication
Lost to follow-up:
0 %</td>
<td></td>
<td></td>
<td><table>
<tr><th></th><th>Pre-lockdown
(M (SD))</th><th>During lockdown
(M (SD))</th><th>Post-lockdown
(M (SD))</th></tr>
<tr><td>Physical activity (minutes/ week)</td><td>1,676 (21)</td><td>n.r.</td><td>1,775 (34)</td></tr>
</table>
- statistically significant difference in physical activity measured as MET-min/week, absolute, or categorical physical activity levels (3x3 ANOVA): higher physical activity during and after lockdown than before

- a similar pattern of results was obtained for males and females, either for categorial or continuous physical activity levels</td>
<td>Committee of University “La Sapenzia”
+ complete sampling at participating schools
+ high response
+ no lost to follow-up
+ outcome measurement with a validated instrument
+ using ANOVA to investigate differences
+ statistical analysis of the confounders “age” (homogeneous group) and “sex” (stratification)
+ temporal investigation

- decrease in group sample sizes throughout the observed time points (personal communication)</td>
</tr>
<tr>
<td colspan="7">SPECIFIC HEALTHY POPULATIONS</td>
</tr>
<tr>
<td>Anyan et al., 2020, Ernsten & Havnen 2020</td>
<td>Study design:
Cross-sectional study
Study name:
n.a.
Country of study:
Norway
Time of study:
June 3–15, 2020
No. of waves:
n = 1
Follow-up duration:
n.a.</td>
<td>Description:
Physically active adults (members of one Norwegian fitness association)
Inclusion criteria:
n.r.
# invited:
n = 6,766
# baseline:
n = 1,314
# follow-up:
n.a.
Age at baseline:
M: 49 years (SD: 11.5, range: 18–81)
% females:
30.8 %</td>
<td>COVID-19 pandemic lockdown:
- Norwegian physical and social distancing regulations (March 12–June 15, 2020):</td>
<td>(Change in) physical activity:
Self-reported single question</td>
<td>Table 1: Descriptive statistics for the change in physical activity since lockdown
<table>
<tr><th></th><th>Prevalence (n (%))</th></tr>
<tr><td colspan="2">Total sample</td></tr>
<tr><td>Reduction</td><td>183 (14)</td></tr>
<tr><td>Increase</td><td>286 (22)</td></tr>
<tr><td>No change</td><td>845 (64)</td></tr>
<tr><td colspan="2">Females</td></tr>
<tr><td>Reduction</td><td>65 (16.0)</td></tr>
<tr><td>Increase</td><td>93 (23.0)</td></tr>
<tr><td>No change</td><td>247 (61.0)</td></tr>
<tr><td colspan="2">Males</td></tr>
<tr><td>Reduction</td><td>118 (13.1)</td></tr>
<tr><td>Increase</td><td>192 (21.2)</td></tr>
<tr><td>No change</td><td>594 (65.7)</td></tr>
</table></td>
<td>Overall study quality:
High risk
Conflict of interest:
Stated (none declared)
Funding:
Commercial funds

+ ethical approval from the Regional Committees for Medical and Health Research Ethics board and the Norwegian Centre for Research Data
+ complete sampling
+ consideration of the confounder “sex” (by stratification)</td>
</tr>
</table>

<table>
<tr><th>Reference
(First author, publication year)</th><th>General
(Study design
Study name
Country of study
Time of study
No. of waves
Follow-up duration
(e.g., mean, range))</th><th>Population
(Description
Inclusion criteria
invited
baseline
follow-up
Age at baseline
% females
Response
Lost to follow-up)</th><th>Exposure
(Description & assessment)</th><th>Outcome
(Description & assessment)</th><th>Results
(Description of all topically relevant results)</th><th>Comments
(study quality [overall assessment according to Iljaz/Shamliyan/SIGN/CASP]
Conflict of interest [stated vs. not stated]
Funding (public, non-profit, commercial)
Strengths (+) and weaknesses (-))</th></tr>
<tr><td></td><td></td><td>Response:
19.4 %
Lost to follow-up:
n.a.</td><td></td><td></td><td></td><td>- only members of a Norwegian fitness association were invited
- low response
- self-reported outcome measurement with a single item (self-developed)
- descriptive analysis only
- no consideration of the confounder “age” in the statistical analysis
- due to study design no actual chronological investigation</td></tr>
<tr><td>Barkley et al., 2020</td><td>Study design:
Cross-sectional study
Study name:
n.a.
Country of study:
United States
Time of study:
May 18–June 18, 2020
No. of waves:
n = 1
Follow-up duration:
n.a.</td><td>Description:
University students, faculty & staff (results not extracted)
Inclusion criteria:
n.r.
# invited:
n = 5,000 (out of 27,716 university students)
# baseline:
n = 184 (undergraduate students: n = 100, graduate students: n = 84)
# follow-up:
n.a.
Age at baseline:
Undergraduate students: M: 26.9 years (SD: 8.9)
Graduate students: M: 29.9 years (SD: 8.7)
% females:</td><td>Campus closure due to the COVID-19 pandemic (since March 11, 2020):
- cancellation of face-to-face classes
March 11, 2020)
- closure of campus, including all fitness facilities, students were sent home (March 20, 2020)
- governor’s “stay at home” order (March 22, 2020)</td><td>Physical activity:
Godin physical activity questionnaire:
- mild physical activity: activities that require “minimal effort”
- moderate physical activity: activities that are “not exhausting”
- strenuous physical activity: activities in which the participant’s “heart beats rapidly”
Sedentary behaviour:
International Physical Activity Questionnaire^
Weight:
Self-reported question

Retrospective assessment of levels prior to March 11, 2020 using the same instruments.</td><td>Table 1: Descriptive statistics for the change in Godin physical activity score since campus closure:
<table>
<tr><th></th><th>Pre-campus closure
(M (SD))</th><th>Post-campus closure
(M (SD))</th></tr>
<tr><td colspan="3">Mild physical activity</td></tr>
<tr><td>Undergraduate students</td><td>16.3 (22.6)</td><td>10.8 (12.9)</td></tr>
<tr><td>Graduate students</td><td>12.0 (22.4)</td><td>11.2 (11.7)</td></tr>
<tr><td colspan="3">Moderate physical activity</td></tr>
<tr><td>Undergraduate students</td><td>15.0 (15.7)</td><td>12.9 (12.4)</td></tr>
<tr><td>Graduate students</td><td>17.1 (36.9)</td><td>16.6 (19.7)</td></tr>
<tr><td colspan="3">Strenuous physical activity</td></tr>
<tr><td>Undergraduate students</td><td>16.0 (22.1)</td><td>14.0 (17.9)</td></tr>
<tr><td>Graduate students</td><td>19.1 (32.9)</td><td>21.0 (33.7)</td></tr>
<tr><td colspan="3">Total physical activity</td></tr>
<tr><td>Undergraduate students</td><td>47.2 (40.2)</td><td>37.7 (30.7)</td></tr>
</table></td><td>Overall study quality:
High risk
Conflict of interest:
Stated (none declared)
Funding:
None

+ ethical approval from the university Institutional Review Board
+ random sampling
+ measurement of the outcomes “physical activity” and “sedentary behavior” with validated instruments
+ using ANOVA to investigate differences
+ statistical analysis of confounders “sex” (interaction analysis) and “age” (homogeneous group)
+ moderator analysis of activity levels before face-to-face classes ceased.</td></tr>
</table>

<table>
<tr>
<th>Reference
(First author, publication year)</th>
<th>General
(Study design
Study name
Country of study
Time of study
No. of waves
Follow-up duration
(e.g., mean, range))</th>
<th>Population
(Description
Inclusion criteria
invited
baseline
follow-up
Age at baseline
% females
Response
Lost to follow-up)</th>
<th>Exposure
(Description & assessment)</th>
<th>Outcome
(Description & assessment)</th>
<th>Results
(Description of all topically relevant results)</th>
<th>Comments
(study quality [overall assessment according to Iljaz/Shamliyan/SIGN/CASP]
Conflict of interest [stated vs. not stated]
Funding (public, non-profit, commercial)
Strengths (+) and weaknesses (-))</th>
</tr>
<tr>
<td></td>
<td></td>
<td>73.2 % (of all participants incl. university staff)
Response:
3.7 % (personal communication)
Lost to follow-up:
n.a.</td>
<td></td>
<td></td>
<td>
<table>
<tr><td>Graduate students</td><td>48.2 (75.2)</td><td>48.7 (58.8)</td></tr>
</table>
- no statistically significant (p ≥ 0.16) main or interaction effects of time for moderate, vigorous, or total physical activity for the university role group by gender by time analyses
- statistically significant (p = 0.003) university role by time interaction for mild physical activity due to a significant (p = 0.015, d = 0.33) decrease in mild physical activity from pre- to post-cancellation in undergraduate students with no statistically significant (p ≥ 0.50, d ≤ 0.18) changes in mild physical activity for any of the other university role groups
- no additional main or interaction effects for differences in mild physical activity (p ≥ 0.17)

Table 2: Descriptive statistics for the change in sedentary behavior (minutes/week) since campus closure:
<table>
<tr><th></th><th>Pre-campus closure
(M (SD))</th><th>Post-campus closure
(M (SD))</th></tr>
<tr><td>Undergraduate students</td><td>3089.2 (1455.4)</td><td>3681.0 (1600.3)</td></tr>
<tr><td>Graduate students</td><td>3129.1 (1329.7)</td><td>3696.4 (1566.5)</td></tr>
</table>
- statistically significant (p = 0.003) main effect of time for sedentary behavior (participants significantly increased average weekly sitting from pre- to post-cancellation)
- no additional main or interaction effects for differences in sedentary behavior (p ≥ 0.18)

Table 2: Descriptive statistics for the change in bodyweight (pounds (lbs.)) since campus closure:
<table>
<tr><th></th><th>Pre-campus closure
(M (SD))</th><th>Post-campus closure
(M (SD))</th></tr>
</table>
</td>
<td>- monocentric study
- low response
- measurement of the outcome “weight” with a self-reported question
- exclusion of people not identifying as male or female
- retrospective assessment of activity levels, sedentary behaviour, and bodyweight before lockdown (recall bias)
- due to study design no actual chronological investigation</td>
</tr>
</table>

<table>
<tr>
<th>Reference
(First author, publication year)</th>
<th>General
(Study design
Study name
Country of study
Time of study
No. of waves
Follow-up duration
(e.g., mean, range))</th>
<th>Population
(Description
Inclusion criteria
invited
baseline
follow-up
Age at baseline
% females
Response
Lost to follow-up)</th>
<th>Exposure
(Description & assessment)</th>
<th>Outcome
(Description & assessment)</th>
<th>Results
(Description of all topically relevant results)</th>
<th>Comments
(study quality [overall assessment according to Iljaz/Shamliyan/SIGN/CASP]
Conflict of interest [stated vs. not stated]
Funding (public, non-profit, commercial)
Strengths (+) and weaknesses (-))</th>
</tr>
<tr>
<td></td>
<td></td>
<td></td>
<td></td>
<td></td>
<td><table>
<tr><td>Undergraduate students</td><td>175.4 (48.4)</td><td>176.8 (48.4)</td></tr>
<tr><td>Graduate students</td><td>163.7 (45.6)</td><td>164.5 (45.6)</td></tr>
</table>
- no statistically significant (p ≥ 0.16) main or interaction effects of time for bodyweight</td>
<td></td>
</tr>
<tr>
<td>Bourion-Bedes et al., 2021</td>
<td>Study design:
Cross-sectional study
Study name:
Feelings and Psychological Impact of the COVID-19 Epidemic among Students in the Grand Est Area (PIMS-CoV19) study
Country of study:
France
Time of study:
May 7–17, 2020
No. of waves:
n = 1
Follow-up duration:
n.a.</td>
<td>Description:
Students
Inclusion criteria:
n.r.
# invited:
n = around 50,000 (personal communication)
# baseline:
n = 3,936
# follow-up:
n.a.
Age at baseline:
M: 21.7 years (SD: 4.0)
% females:
70.6 %
Response:
Around 7.9 % (personal information)
Lost to follow-up:
n.a.</td>
<td>Lockdown due to the COVID-19 outbreak</td>
<td>(Change in) alcohol consumption:
Self-reported single question
(Change in) tobacco consumption:
Self-reported single question</td>
<td>Table 1: Descriptive statistics for the change in alcohol and tobacco consumption since lockdown
<table>
<tr><td></td><td>Prevalence (%)</td></tr>
<tr><td colspan="2">Alcohol consumption</td></tr>
<tr><td>None</td><td>34.2</td></tr>
<tr><td>No change</td><td>17.1</td></tr>
<tr><td>Increased</td><td>13.7</td></tr>
<tr><td>Reduced</td><td>35.0</td></tr>
<tr><td colspan="2">Tobacco consumption</td></tr>
<tr><td>None</td><td>83.5</td></tr>
<tr><td>No change</td><td>3.0</td></tr>
<tr><td>Increased</td><td>7.2</td></tr>
<tr><td>Reduced</td><td>6.3</td></tr>
</table></td>
<td>Overall study quality:
High risk
Conflict of interest:
Stated (none declared)
Funding:
None

+ complete sampling at 2 universities
+ consideration of the confounder “age” (homogeneous group)

- low response
- outcome measurement with a self-reported question
- no consideration of the confounder “sex” in the statistical analysis
- descriptive analysis only
- due to study design no actual chronological investigation</td>
</tr>
<tr>
<td>Di Sebastiano et al., 2020</td>
<td>Study design:
(Prospective) secondary data analysis
Study name:
n.a.
Country of study:
Canada
Time of study:</td>
<td>Description:
Adults (≥ 18 years) using a physical activity tracking ParticipACTION app (PAC app)
Inclusion criteria:
n.r.
invited:</td>
<td>Physical distancing protocols</td>
<td>Physical activity:
Measured using data from PAC app, that is compatible and draws data from 3 manufacturers of wearable devices and their associated apps (Garmin, Fitbit, and Apple Watch), and</td>
<td>Weekly physical activity: T0–T1
<table>
<tr><td></td><td>T0
(M (SE))</td><td>T1
(M (SE))</td><td>p</td></tr>
<tr><td>Moderate-to-vigorous physical activity (minutes)</td><td>194.2 (5.2)</td><td>176.7 (5.0)</td><td>< 0.001</td></tr>
</table></td>
<td>Overall study quality:
High risk
Conflict of interest:
Stated (2 authors are employed by the PAC app, 1 author is chair of the ParticipACTION Research Advisory Group, 1 author declares no conflict of interest)</td>
</tr>
</table>

<table>
<tr><th>Reference
(First author, publication year)</th><th>General
(Study design
Study name
Country of study
Time of study
No. of waves
Follow-up duration
(e.g., mean, range))</th><th>Population
(Description
Inclusion criteria
invited
baseline
follow-up
Age at baseline
% females
Response
Lost to follow-up)</th><th>Exposure
(Description & assessment)</th><th>Outcome
(Description & assessment)</th><th>Results
(Description of all topically relevant results)</th><th>Comments
(study quality [overall assessment according to Iljaz/Shamliyan/SIGN/CASP]
Conflict of interest [stated vs. not stated]
Funding (public, non-profit, commercial)
Strengths (+) and weaknesses (-))</th></tr>
<tr><td></td><td>February 10–April 19, 2020
T0: 4 weeks prior physical distancing protocols
T1: 1 weeks after the beginning of the physical distancing protocols
T2: 6 weeks after physical distancing protocols
No. of waves:
n = 3
Follow-up duration:
10 weeks</td><td>n = 23,173 (Canadians who logged on to the PAC app at least once)
# baseline:
n = 2.338 (PAC app user who had complete datasets)
# follow-up:
n.a.
Age at baseline:
18–24 years: 3.0 %,
25–34 years: 13.6 %,
35–44 years: 26.6 %,
45–54 years: 25.6 %,
55–64 years: 23.1 %,
≥ 65 years: 8.0 %, unknown: 0.2 %
% females:
90.2 %
% active users (of those registered with the app) providing data:
10.1 %
Lost to follow-up:
n.a. (only complete cases investigated)</td><td></td><td>2 activity tracking apps with no associated wearable devices (Apple Health and Google Fit)
- for wearable devices, physical activity intensity was defined based on the device specific definitions, which typically use heart rate or step cadence
- Moderate-to-vigorous physical activity (MVPA): step rate ≥ 100 steps/minute
- Light physical activity (LPA): < 100 steps/minute
- Steps (number of steps)</td><td><table><tr><td>Light physical activity (minutes)</td><td>1,000.5 (17.0)</td><td>874.1 (15.6)</td><td>< 0.001</td></tr><tr><td>Steps</td><td>48,625 (745)</td><td>43,395 (705)</td><td>< 0.001</td></tr></table>
Weekly physical activity: T0–T2<table><tr><th></th><th>T0
(M (SE))</th><th>T2
(M (SE))</th><th>p</th></tr><tr><td>Moderate-to-vigorous physical activity (minutes)</td><td>194.2 (5.2)</td><td>204.4 (5.4)</td><td>0.498</td></tr><tr><td>Light physical activity (minutes)</td><td>1,000.5 (17.0)</td><td>732.0 (14.3)</td><td>< 0.001</td></tr><tr><td>Steps</td><td>48,625 (745)</td><td>41,946 (763)</td><td>< 0.001</td></tr></table>Repeated measure ANOVA

- only selective significant differences for age groups
- no interaction observed by gender</td><td>Funding:
None

+ ethical approval from the Behavioral Research Ethics Board University of British Columbia
+ using ANOVA to investigate differences
+ statistical analysis of confounders “sex” and “age”
+ chronological investigation

- only data used from those app users who used the app every week throughout the 10 weeks of investigation
- selection bias due to volunteer bias (health-interested and technology-savvy users)
- app data may objectively measure walking levels (steps), but do not measure other types of physical activities (underestimation of the actual physical activity level of an individual)</td></tr>
<tr><td>Lechner et al., 2020</td><td>Study design:
Cross-sectional study
Study name:
n.a.
Country of study:
United States
Time of study:
March 26–31, 2020
No. of waves:
n = 1</td><td>Description:
Students using alcohol in the past 30 days
Inclusion criteria:
n.r.
# invited:
n = 33,280
# baseline:
n = 1,958
# follow-up:
n.a.</td><td>University closings (on March 11, 2020)</td><td>Alcohol consumption:
Timeline Follow-Back Interview administered to document alcohol use in the 2-weeks between March 4– 17, 2020

Retrospective assessment of levels prior to university closing using the same instruments</td><td>Text: Descriptive statistics for the change in alcohol consumption since university closing<table><tr><th></th><th>Week prior to university closing (4–10, March 2020)
(M (SD))</th><th>Week succeeding university closing (11–17, March 2020)
(M (SD))</th></tr></table></td><td>Overall study quality:
High risk
Conflict of interest:
Stated (none declared)
Funding:
Unclear

+ complete sampling
+ validated outcome measure</td></tr>
</table>

<table>
<tr>
<th>Reference
(First author, publication year)</th>
<th>General
(Study design
Study name
Country of study
Time of study
No. of waves
Follow-up duration
(e.g., mean, range))</th>
<th>Population
(Description
Inclusion criteria
invited
baseline
follow-up
Age at baseline
% females
Response
Lost to follow-up)</th>
<th>Exposure
(Description & assessment)</th>
<th>Outcome
(Description & assessment)</th>
<th>Results
(Description of all topically relevant results)</th>
<th>Comments
(study quality [overall assessment according to Iljaz/Shamliyan/SIGN/CASP]
Conflict of interest [stated vs. not stated]
Funding (public, non-profit, commercial)
Strengths (+) and weaknesses (-))</th>
</tr>
<tr>
<td></td>
<td>Follow-up duration:
n.a.</td>
<td>Age at baseline:
M: 24.94 years (SD: 7.65)
% females:
79.97 %
Response:
Students overall:
12.8 %
Lost to follow-up:
n.a.</td>
<td></td>
<td></td>
<td><table>
<tr><td>Number of weekly standard drinks</td><td>3.48 (5.45)</td><td>5.01 (6.86)</td></tr>
<tr><td>Number of drinking days</td><td>1.36 (1.55)</td><td>1.94 (1.84)</td></tr>
</table>
- Regression coefficient for time (main effect): b = 0.396 (95 % CI: 0.316 –423, p < 0.001) (statistically significant increase in alcohol consumption over time)</td>
<td>+ general estimating equation (GEE) regression analysis with negative binomial distribution adjusted for race/ethnicity, grade-level, biological sex
+ consideration of age (heterogeneous group)

- monocentric study
- low response
- high percentage of female students
- retrospective assessment of alcohol consumption before lockdown (recall bias)
- due to study design no actual chronological investigation</td>
</tr>
<tr>
<td>Özden & Kilic, 2021</td>
<td>Study design:
Cross-sectional study
Study name:
n.a.
Country of study:
Turkey
Time of study:
May 15–29, 2020
No. of waves:
n = 1
Follow-up duration:
n.a.</td>
<td>Description:
Nursing students
Inclusion criteria:
n.r.
# invited:
n = 1,400
# baseline:
n = 1,011
# follow-up:
n.a.
Age at baseline:
M: 19.97 years (SD: 3.11)
% females:
60 %
Response:
72.2 %
Lost to follow-up:
n.a.</td>
<td>Lockdown (social isolation) due to COVID-19 pandemic:
- closure of all schools and universities on March 16, 2020
- university students continued education with distance learning possibilities</td>
<td>(Change in) weight:
Self-reported question
Exercise:
Self-reported question (retrospective assessment of levels before COVID-19 outbreak using the same instruments)</td>
<td>Table 1: Descriptive statistics for the change in weight since lockdown
<table>
<tr><th></th><th>Prevalence (%)</th></tr>
<tr><td>Same weight</td><td>19.7</td></tr>
<tr><td>Weight gain</td><td>46.9</td></tr>
<tr><td>Weight loss</td><td>33.4</td></tr>
</table>
Table 1: Descriptive statistics for the change in regular exercise since lockdown
<table>
<tr><th></th><th>Before COVID-19 outbreak (%)</th><th>During lockdown (%)</th></tr>
<tr><td>Regular exercise every day</td><td>32.6</td><td>43.3</td></tr>
</table></td>
<td>Overall study quality:
High risk
Conflict of interest:
Stated (none declared)
Funding:
None

+ ethical approval from the he İnönü University Health Sciences Scientific Research and Publication Ethics Committee
+ complete sampling
+ high response
+ consideration of the confounder “age” (homogeneous group)

- monocentric study</td>
</tr>
</table>

<table>
<tr><th>Reference
(First author, publication year)</th><th>General
(Study design
Study name
Country of study
Time of study
No. of waves
Follow-up duration
(e.g., mean, range))</th><th>Population
(Description
Inclusion criteria
invited
baseline
follow-up
Age at baseline
% females
Response
Lost to follow-up)</th><th>Exposure
(Description & assessment)</th><th>Outcome
(Description & assessment)</th><th>Results
(Description of all topically relevant results)</th><th>Comments
(study quality [overall assessment according to Iljaz/Shamliyan/SIGN/CASP]
Conflict of interest [stated vs. not stated]
Funding (public, non-profit, commercial)
Strengths (+) and weaknesses (-))</th></tr>
<tr><td></td><td></td><td></td><td></td><td></td><td></td><td>- outcome measurement with a self-reported question
- no consideration of the confounder “sex” in the statistical analysis
- descriptive analysis only
- retrospective assessment of exercise levels before lockdown (recall bias)
- due to study design no actual chronological investigation</td></tr>
<tr><td>Savage et al., 2020</td><td>Study design:
Cohort study
Study name:
Student Health Study
Country of study:
United Kingdom
Time of study:
Pre-COVID-19:
T1: October 14, 2019
T2: January 28, 2020
During COVID-19:
T3: March 20, 2020 (1. week of lockdown)
T4: April 27, 2020 (5. week of lockdown)
No. of waves:
n = 4
Follow-up duration:
6–8 month</td><td>Description:
University students
Inclusion criteria:
n.r.
# invited:
n = 9,472
# baseline:
n = 1,477
# follow-up:
n = 214 (those who completed ≥3 assessments, including T1 and T4)
Age at baseline:
M: 28.0 years (final sample)
% females:
72.0 % (final sample)
Response:
15.6 %
Lost to follow-up:
85.5 %</td><td>Lockdown:
- requirement to stay at home as much as possible
- people were allowed only to leave home once per day for exercise</td><td>Physical activity:
Exercise Vital Sign (EVS) questionnaire to assess moderate to vigorous physical activity levels (MVPA)
Sedentary behaviour:
1 self-reported question</td><td>Figure 1: Changes in sedentary behaviour and physical activity
<table><tr><th></th><th>p</th><th>Cohens´ d</th></tr><tr><td colspan="3">Sedentary behaviour</td></tr><tr><td>Time spent in sedentary behaviour on a typical day in the last month</td><td>< 0.0001*</td><td>0.78</td></tr><tr><td colspan="3">Physical activity</td></tr><tr><td>MVPA</td><td>< 0.01**</td><td>0.12</td></tr></table>Repeated-measures ANCOVA
*no interactions between gender and changes in sedentary behaviour were found
**significant gender interaction, reflecting that MVPA was higher in males at T1, but not at T4 (p < 0.05)</td><td>Overall study quality:
High risk
Conflict of interest:
Stated (none declared)
Funding:
None

+ ethical approval from the Science and Technology College Research Ethics Committee at Nottingham Trent University
+ complete sampling
+ measuring physical activity with a validated questionnaire
+ using ANCOVA to investigate differences
+ consideration of the confounders “age” (homogeneous group) and “sex” (interaction analysis)
+ temporal investigation

- monocentric study
- low response and high lost to follow-up</td></tr>
</table>

<table>
<tr><th>Reference
(First author, publication year)</th><th>General
(Study design
Study name
Country of study
Time of study
No. of waves
Follow-up duration
(e.g., mean, range))</th><th>Population
(Description
Inclusion criteria
invited
baseline
follow-up
Age at baseline
% females
Response
Lost to follow-up)</th><th>Exposure
(Description & assessment)</th><th>Outcome
(Description & assessment)</th><th>Results
(Description of all topically relevant results)</th><th>Comments
(study quality [overall assessment according to Iljaz/Shamliyan/SIGN/CASP]
Conflict of interest [stated vs. not stated]
Funding (public, non-profit, commercial)
Strengths (+) and weaknesses (-))</th></tr>
<tr><td></td><td></td><td></td><td></td><td></td><td></td><td>- measuring sedentary behaviour with a single-question</td></tr>
<tr><td>To et al., 2021</td><td>Study design:
(Prospective) secondary data analysis
Study name:
10,000 Steps program
Country of study:
Australia
Time of study:
January 1, 2018-June 30, 2020
T1 (lockdown): March 2, 2020 (7 and 30 days before and after)
T2 (lockdown relaxation): May 8, 2020 (7 and 30 days before and after)
No. of waves:
n = 2 (continuous data collection)
Follow-up duration:
10 weeks</td><td>Description:
Adults, who are registered as members of the 10,000 Steps program
Inclusion criteria:
people living in Australia, aged 18 to 80 years, with at least 7 days of logged data, days with logged steps that were fewer than 1,000 steps per day) or more than 40,000 steps were considered invalid and excluded
# invited:
n = 463,000 (total number of members who registered an account with the program website)
# baseline:
n = 60,560 (data from registered members of the program between January 1, 2018, and June 30, 2020)
# follow-up:
n.a.
Age at baseline:
18–45 years: 59.7 %, 46–80 years: 40.3 %
% females:
67.0. %</td><td>Lockdown (start on March 2 2020):
- social distancing guidelines
- closure of nonessential businesses, such as gyms, indoor sports facilities, and clubs
- people were allowed to be outside only for exercise or other essential needs
- restaurants and cafes only offered takeaway and delivery services
Relaxation of restrictions (start on May 8, 2020)</td><td>Physical activity:
Number of steps logged per day (entered manually by the members using pedometers or synced automatically with activity trackers connected to the program)</td><td>Table 2: 7-day average of steps per day before and after each event
<table>
<tr><th></th><th>Before (steps/ day)</th><th>After (steps/ day)</th><th>p</th></tr>
<tr><td colspan="4">Begin of lockdown</td></tr>
<tr><td>All</td><td>9,500</td><td>9,175</td><td>< 0.001</td></tr>
<tr><td>Male</td><td>9,716</td><td>9,270</td><td>< 0.001</td></tr>
<tr><td>Female</td><td>9,391</td><td>9,130</td><td>< 0.001</td></tr>
<tr><td>18–45 years</td><td>9,113</td><td>8,739</td><td>< 0.001</td></tr>
<tr><td>46–80 years</td><td>10,001</td><td>9,739</td><td>< 0.001</td></tr>
<tr><td colspan="4">Relaxation of restrictions</td></tr>
<tr><td>All</td><td>9,637</td><td>9,767</td><td>< 0.001</td></tr>
<tr><td>Male</td><td>9,887</td><td>10,035</td><td>0,004</td></tr>
<tr><td>Female</td><td>9,489</td><td>9,606</td><td>< 0.001</td></tr>
<tr><td>18–45 years</td><td>9,181</td><td>9,335</td><td>< 0.001</td></tr>
<tr><td>46–80 years</td><td>10,322</td><td>10,415</td><td>0.02</td></tr>
</table>
Wilcoxon signed-rank tests

Table 2: 30-day average of steps per day before and after each event
<table>
<tr><th></th><th>Before (steps/ day)</th><th>After (steps/ day)</th><th>p</th></tr>
<tr><td colspan="4">Begin of lockdown</td></tr>
<tr><td>All</td><td>9,684</td><td>9,199</td><td>< 0.001</td></tr>
<tr><td>Male</td><td>9,938</td><td>9,381</td><td>< 0.001</td></tr>
<tr><td>Female</td><td>9,555</td><td>9,112</td><td>< 0.001</td></tr>
<tr><td>18–45 years</td><td>9,367</td><td>8,758</td><td>< 0.001</td></tr>
<tr><td>46–80 years</td><td>10,096</td><td>9,773</td><td>< 0.001</td></tr>
<tr><td colspan="4">Relaxation of restrictions</td></tr>
<tr><td>All</td><td>9,477</td><td>9,833</td><td>< 0.001</td></tr>
<tr><td>Male</td><td>9,723</td><td>10,137</td><td>< 0.001</td></tr>
</table></td><td>Overall study quality:
High risk
Conflict of interest:
Stated (none declared)
Funding:
Public funds

+ using Wilcoxon signed-rank test to investigate differences
+ statistical analysis stratified by confounders “sex” and “age” (by stratification)
+ chronological investigation

- selection bias due to volunteer bias (health-interested and technology-savvy users)
- only data used from those app users who used the app every week throughout the 10 weeks of investigation
- app data may objectively measure walking levels (steps), but do not measure other types of physical activities (underestimation of the actual physical activity level of an individual)</td></tr>
</table>

<table>
<tr>
<th>Reference
(First author, publication year)</th>
<th>General
(Study design
Study name
Country of study
Time of study
No. of waves
Follow-up duration
(e.g., mean, range))</th>
<th>Population
(Description
Inclusion criteria
invited
baseline
follow-up
Age at baseline
% females
Response
Lost to follow-up)</th>
<th>Exposure
(Description & assessment)</th>
<th>Outcome
(Description & assessment)</th>
<th>Results
(Description of all topically relevant results)</th>
<th>Comments
(study quality [overall assessment according to Iljaz/Shamliyan/SIGN/CASP]
Conflict of interest [stated vs. not stated]
Funding (public, non-profit, commercial)
Strengths (+) and weaknesses (-))</th>
</tr>
<tr>
<td></td>
<td></td>
<td>% active users (of those registered with the app) providing data:
13.1 %
Lost to follow-up:
n.a</td>
<td></td>
<td></td>
<td>
<table>
<tr><td>Female</td><td>9,330</td><td>9,649</td><td>< 0.001</td></tr>
<tr><td>18–45 years</td><td>9,060</td><td>9,426</td><td>< 0.001</td></tr>
<tr><td>46–80 years</td><td>10,106</td><td>10,446</td><td>< 0.001</td></tr>
</table>
Wilcoxon signed-rank tests
* effects were not different for genders and age groups
</td>
<td></td>
</tr>
<tr>
<td>Wang et al., 2020</td>
<td>Study design:
Cohort study
Study name:
Step Study
Country of study:
China
Time of study:
T0: corresponding period in 2019
T1: 30 days before January 21, 2020 (reference period)
T2: 30 days after January 21, 2020 (epidemic period)
(January 21, 2020: date of the first COVID-19 case diagnosed in Changsha)
No. of waves:
n = 3
Follow-up duration:
Around 1 year</td>
<td>Description:
Middle-aged and older adults
Inclusion criteria:
Residents aged ≥ 40 years, owning a personal smartphone and having a WeChat account, exclusion of residents with severe mental illness
# invited:
n = 7,262 (September 2018–January 2020)
# baseline:
n = 4,145
# follow-up:
n = 3,544
Age at baseline:
M: 51.6 years (SD: 8.9)
% females:
34.6 %
Response:
57.1 %
Lost to follow-up:
15.0 %</td>
<td>Physical distancing measures</td>
<td>Walking activity:
- Daily steps collected via a smartphone linked to WeChat (participants were required to wear their smartphone for≥ 10 hours on a given day)
- Prevalence of low daily steps (≤ 1500 steps/day)
- Prevalence of frequent low daily steps cases (≤ 1500 steps/day for ≥ 14 days)</td>
<td>
Table 2: Associations of time period with daily step count
<table>
<tr><th rowspan="2">Time period</th><th colspan="2">2019</th><th colspan="2">2020</th></tr>
<tr><th>M (SD)</th><th>MD (95 CI)</th><th>M (SD)</th><th>MD (95 CI)</th></tr>
<tr><td>Reference</td><td>7872 (4842)</td><td>0 (reference)</td><td>8097 (4793)</td><td>0 (reference)</td></tr>
<tr><td>Epidemic</td><td>7472 (4979)</td><td>-413 (-501–(-325))</td><td>5440 (4571)</td><td>-2672 (-2763–(-2582))</td></tr>
</table>
Table 3: Associations of time period with prevalence of low daily steps
<table>
<tr><th rowspan="2">Time period</th><th colspan="2">2019</th><th colspan="2">2020</th></tr>
<tr><th>Person-day, n (%)</th><th>PR (95 CI)</th><th>Person-day, n (%)</th><th>PR (95 CI)</th></tr>
<tr><td>Reference</td><td>1006 (3.3)</td><td>1.0 (reference)</td><td>2287 (3.0)</td><td>1.0 (reference)</td></tr>
<tr><td>Epidemic</td><td>1488 (5.1)</td><td>1.6 (1.4–1.7)</td><td>12,951 (18.5)</td><td>6.2 (5.8–6.7)</td></tr>
</table>
Table 4: Prevalence of frequent low daily steps cases (≤ 1500 steps/day for ≥ 14 days)
<table>
<tr><th>Frequent low daily steps cases</th><th>T1
n (%)</th><th>T2
n (%)</th><th>p</th></tr>
<tr><td>Total sample</td><td>12 (0.4)</td><td>196 (7.4)</td><td>n.a.</td></tr>
<tr><td colspan="3">Sex</td><td></td></tr>
</table>
</td>
<td>Overall study quality:
High risk
Conflict of interest:
Stated (none declared)
Funding:
Public funds

+ ethical approval from Ethics Committee of Xiangya Hospital, Central South University
+ complete sampling
+ long-term monitoring of daily step count trends
+ using a statistical test to investigate differences
+ stratification for confounders “sex” and “age” in statistical analysis
+ temporal investigation

- only those who had their annual physical check-up and who used WeChat were invited
- app data may objectively measure walking levels (steps), but do not measure other types of physical activities (underestimation of the actual physical activity level of an individual)</td>
</tr>
</table>

<table>
<thead>
<tr>
<th>Reference
(First author, publication year)</th>
<th>General
(Study design
Study name
Country of study
Time of study
No. of waves
Follow-up duration
(e.g., mean, range))</th>
<th>Population
(Description
Inclusion criteria
invited
baseline
follow-up
Age at baseline
% females
Response
Lost to follow-up)</th>
<th>Exposure
(Description & assessment)</th>
<th>Outcome
(Description & assessment)</th>
<th>Results
(Description of all topically relevant results)</th>
<th>Comments
(study quality [overall assessment according to Iljaz/Shamliyan/SIGN/CASP]
Conflict of interest [stated vs. not stated]
Funding (public, non-profit, commercial)
Strengths (+) and weaknesses (-))</th>
</tr>
</thead>
<tbody>
<tr>
<td></td>
<td></td>
<td></td>
<td></td>
<td></td>
<td>
<table>
<tr><td>Males</td><td>7 (0.4)</td><td>75 (4.2)</td><td rowspan="2">< 0.001*</td></tr>
<tr><td>Females</td><td>5 (0.5)</td><td>121 (14.0)</td></tr>
<tr><td colspan="4">Age</td></tr>
<tr><td>40–49 years</td><td>1 (0.1)</td><td>84 (5.7)</td><td rowspan="4">0.001**</td></tr>
<tr><td>50–59 years</td><td>8 (0.8)</td><td>69 (7.9)</td></tr>
<tr><td>60–69 years</td><td>2 (0.7)</td><td>32 (12.5)</td></tr>
<tr><td>≥ 70 years</td><td>1 (1.4)</td><td>11 (19.3)</td></tr>
</table>
*interaction between change in prevalence of frequent low daily steps and sex: prevalence of frequent low daily steps increased more among women
*interaction between change in prevalence of frequent low daily steps and age: prevalence of frequent low daily steps increased more among older people
</td>
<td>- previous studies found that iOS and Android devices have different accuracies in capturing the daily step count under free-living conditions (misclassification of daily step counts)</td>
</tr>
<tr>
<td>White et al., 2021</td>
<td>Study design:
Cross-sectional study
Study name:
3-campus Alcohol and Marijuana Study (original study)
Country of study:
United States
Time of study:
n.r.
No. of waves:
n = 1
Follow-up duration:
n.a.</td>
<td>Description:
College students, who reported drinking alcohol pre- and post-campus closure
Inclusion criteria:
Restriction to first-year students and sophomores at wave 1 assuming that they were still matriculated (as juniors and seniors) in spring 2020
# invited:
n = 473 (those who indicated at the end of wave 2 of the original study that they would like to be contacted for future studies)
# baseline:
n = 297
# follow-up:
n.a.
Age at baseline:</td>
<td>Campus closure because of COVID-19</td>
<td>Drinking:
Daily Drinking Questionnaire with reference to a typical week before campus closed and a typical week since campus closed

Retrospective assessment of levels pre-closure using the same instruments</td>
<td>Text: Overall change of drinking from pre-closure to post-closure
<table>
<tr><th></th><th>Pre-closure (M)</th><th>Post-closure (M)</th><th>p</th><th>d</th></tr>
<tr><td>Drinking frequency (in days)</td><td>3.0</td><td>3.2</td><td><0.05</td><td>0.12</td></tr>
<tr><td>Weekly quantity (drinks/ week)</td><td>11.5</td><td>9.9</td><td><0.01</td><td>0.15</td></tr>
<tr><td>Maximum number of drinks in one day</td><td>4.9</td><td>3.3</td><td><0.001</td><td>0.47</td></tr>
</table>
Paired sampled t-tests</td>
<td>Overall study quality:
High risk
Conflict of interest:
Unclear
Funding:
Public funds

+ ethical approval from the Brown University Institutional Review Board
+ random selection of students from 3 universities in the original study (responses: 30.4–35.7 %)
+ high response
+ non-responder analysis (no significant differences in terms of demographic characteristics) or past-3-months alcohol or cannabis use frequency collected at wave 1)
+ validated questionnaire for outcome measurement
+ using paired t-tests to investigate differences</td>
</tr>
</tbody>
</table>

<table>
<tr><th>Reference
(First author, publication year)</th><th>General
(Study design
Study name
Country of study
Time of study
No. of waves
Follow-up duration
(e.g., mean, range))</th><th>Population
(Description
Inclusion criteria
invited
baseline
follow-up
Age at baseline
% females
Response
Lost to follow-up)</th><th>Exposure
(Description & assessment)</th><th>Outcome
(Description & assessment)</th><th>Results
(Description of all topically relevant results)</th><th>Comments
(study quality [overall assessment according to Iljaz/Shamliyan/SIGN/CASP]
Conflict of interest [stated vs. not stated]
Funding (public, non-profit, commercial)
Strengths (+) and weaknesses (-))</th></tr>
<tr><td></td><td></td><td>M: 21.2 years (SD: 0.82)
% females:
62 %
Response:
66 %
Lost to follow-up:
n.a.</td><td></td><td></td><td></td><td>+ consideration of confounder “age” (homogeneous group)

- no statistical analysis of confounder “sex” in statistical analysis
- retrospective assessment of drinking levels before lockdown (recall bias)
- due to study design no actual chronological investigation</td></tr>
<tr><td>Wickersham et al., 2021</td><td>Study design:
(Prospective) secondary data analysis
Study name:
n.a.
Country of study:
United Kingdom
Time of study:
Start: commencement of lockdown: March 23, 2020
Initial period of lockdown: March 23–May 10, 2020
(7 weeks)
Easing of restrictions: May 11–June 14, 2020
(5 weeks)
No. of waves:
Continuous data collection
Follow-up duration:
12 weeks</td><td>Description:
Students who had enrolled in the remote measurement technolocy (RMT) King’s Move Physical Activity (PA) tracker app
Inclusion criteria:
Having physical activity data available for the study period
invited:
Students using app: n = 1,001 (personal communication)
Eligible sample size: n = 770
# baseline:
n = 736
# follow-up:
n.a.
Age at baseline:
Median: 22 years (IQR: 20–25)
% females:
72.8 %</td><td>COVID-19 lockdown (announced on March 23, 2020):
- closure of services, including fitness centres, hospitality, leisure, and educational institutions
- people should only go outside for one form of exercise per day or to make essential shopping trips
- closure of all university campuses
- easing of restrictions on May 11, 2020: individuals were able to return to work (where they could not work from home), spend time outdoors as often as they would like, and meet one other person from outside their own household</td><td>Physical activity:
Measured using data from app, which collects and aggregates physical activity data from Google Fit and Apple Health fitness tracker apps and operates a rewards-based system.
Physical activity considered:
- steps walked per week
- miles run per week</td><td>Figure 1 and Table 2: Trajectories of steps walked per week:
- gradual increase in steps walked per week over time on average (quadratic effect of week and no interaction terms, adjusted for gender, ethnicity, and age)

Table 2 (excerpt): Slope of outcome on week in the reference group:<table><tr><th>Steps/week</th><th>IRR (95 % CI)</th><th>p</th></tr><tr><td>Linear effect</td><td>1.00 (0.97–1.03)</td><td>0.984</td></tr><tr><td>Quadratic effect</td><td>1.00 (1.00–1.01)</td><td>0.047</td></tr></table>
Figure 2 and Table 3: Trajectories of miles run per week:
- females tended to show a decrease in the number of miles run per week during the observation period, whereas males did not (linear effect of week, and an interaction term between gender and week)

Table 3 (excerpt): Slope of outcome on week in the reference group<table><tr><th>Steps/week</th><th>IRR (95% CI)</th><th>p</th></tr><tr><td>Linear effect</td><td>1.01 (0.99–1.03)</td><td>0.394</td></tr><tr><td>Gender*Week Interaction</td><td></td><td></td></tr></table></td><td>Overall study quality:
High risk
Conflict of interest:
Statement that most authors are employed at King´s College London
Funding:
Public funds

+ ethical approval from the King’s College London Research Ethics Committee
+ app was offered to all students of the university in March 2020 (complete sampling)
+ excluded users were similar to the analytical sample in terms of gender, ethnicity and age, although a slightly higher proportion of those included in running trajectory models were male and from White ethnic groups</td></tr>
</table>

**Reference** (First author, publication year)	**General** (Study design Study name Country of study Time of study No. of waves Follow-up duration (e.g., mean, range))	**Population** (Description Inclusion criteria # invited # baseline # follow-up Age at baseline % females Response Lost to follow-up)	**Exposure** (Description & assessment)	**Outcome** (Description & assessment)	**Results** (Description of all topically relevant results)	**Comments** (study quality [overall assessment according to Iljaz/Shamliyan/SIGN/CASP] Conflict of interest [stated vs. not stated] Funding (public, non-profit, commercial) Strengths (+) and weaknesses (-))
		**% active users (of those registered with the app) providing data:**<br>Students using app: 73.5 % (personal communication)<br>Of all students at the university: 2.2 %<br>**Lost to follow-up:**<br>n.a.			Female | 0.96 (0.94–0.99) | 0.007	+ using negative binomial mixed models to investigate differences<br>+ statistical analysis of confounders "sex" (stratification) and "age" (homogenous group)<br>+ chronological investigation of the trajectory after lockdown and easing of restrictions<br><br>- monocentric study<br>- selection bias due to volunteer bias (health-interested and technology-savvy users)<br>- app data may objectively measure walking levels (steps), but do not measure other types of physical activities (underestimation of the actual physical activity level of an individual)<br>- the app was launched just prior to the UK nationwide lockdown, and it was not possible to ascertain whether physical activity was reduced by lockdown, and if changes observed after lockdown were an artifact of increasing awareness of the app
Yamada et al., 2020	**Study design:**<br>(Prospective) secondary data analysis<br>**Study name:**<br>n.a.<br>**Country of study:**	**Description:**<br>Physically independent residents, living in a continuing care retirement community<br>**Inclusion criteria:**	**Social/physical distancing and self-isolation:**<br>- continuing care retirement community announced the cancellation of all upcoming in-facility events/exhibitions and	**Walking:**<br>Walking distance (in meters/day) within the continuing care retirement community based on behaviour logs from	- after the continuing care retirement community announcement (February 24, 2020) until the state of emergency declaration (April 7, 2020), walking distance gradually decreased at a rate of 0.5% [5.4 meters/day (95 % CI: -10.4–(-0.4))]	**Overall study quality:**<br>High risk<br>**Conflict of interest:**<br>Unclear<br>**Funding:**<br>Unclear

**Reference** (First author, publication year)	**General** (Study design Study name Country of study Time of study No. of waves Follow-up duration (e.g., mean, range))	**Population** (Description Inclusion criteria # invited # baseline # follow-up Age at baseline % females Response Lost to follow-up)	**Exposure** (Description & assessment)	**Outcome** (Description & assessment)	**Results** (Description of all topically relevant results)	**Comments** (study quality [overall assessment according to Iljaz/Shamliyan/SIGN/CASP] Conflict of interest [stated vs. not stated] Funding (public, non-profit, commercial) Strengths (+) and weaknesses (-))
	Japan<br>**Time of study:**<br>January 1–May 25, 2020<br>**No. of waves:**<br>Ccontinuous data collection<br>**Follow-up duration:**<br>around 5 months	n.r.<br>**# invited:**<br>*n* = 296 (personal communication)<br>**# baseline:**<br>*n* = 114<br>**# follow-up:**<br>*n* = 114<br>**Age at baseline:**<br>Range: 67–92 years<br>**% females:**<br>70.4 %<br>**Response:**<br>38.5 % (personal communication)<br>**Lost to follow-up:**<br>0 %	the closure of some common facilities as a precaution measure (February 24, 2020)<br>- state of emergency was declared by the prime minister, asking people to stay at home (April 7, 2020)	beacon transmitter (a senior-friendly internet-of-things device for tracking movement), which were carried by the residents daily	- the state of emergency declaration had a further significant acute impact on the daily walking distance by 20.3% decrease [-186.8 meters (95 %CI: -333.0–(-40.6))]	+ complete sampling<br>+ no lost to follow-up<br>+ using regression analyses to investigate differences<br>+ temporal investigation<br><br>- monocentric study<br>- low response<br>- movement tracking may objectively measure walking distance, but do not measure other types of physical activities (underestimation of the actual physical activity level of an individual)<br>- no consideration of the confounders “age” and “sex” in the statistical analysis<br>- no information, whether an ethical approval existed

Abbreviations: *ANOVA* analysis of variance, *ANCOVA* analysis of covariance, *CI* confidence interval, *d* Cohens´ d, *HDL-C* high-density lipoprotein-cholesterol, *IQR* interquartile range, *IRR* Incident Rate Ratios, *LDL-C* low-density lipoprotein-cholesterol, *M* mean, *MD* mean difference, *MET* metabolic equivalent of task, *n/N* number of, *n.r.* not reported, *ref* reference, *PR* prevalence ratio, *RR* relative risk, *SD* standard deviation, *T* time
